# Supplementary material for: What factors are associated with reporting lacking interest in sex and how do these vary by gender? Findings from the third British national survey of sexual attitudes and lifestyles
Source: BMJ Open. 2017 Sep 13;7(9):e016942. doi: 10.1136/bmjopen-2017-016942 (PMC5722087; doi:10.1136/bmjopen-2017-016942)
Supplement: Author's manuscript [file bmjopen-2017-016942.draft_revisions.pdf]

BMJ Open is committed to open peer review. As part of this commitment we make the peer review history of every article we publish publicly available.

When an article is published we post the peer reviewers' comments and the authors' responses online. We also post the versions of the paper that were used during peer review. These are the versions that the peer review comments apply to.

The versions of the paper that follow are the versions that were submitted during the peer review process. They are not the versions of record or the final published versions. They should not be cited or distributed as the published version of this manuscript.

BMJ Open is an open access journal and the full, final, typeset and author-corrected version of record of the manuscript is available on our site with no access controls, subscription charges or pay-per-view fees (http://bmjopen.bmj.com).

If you have any questions on BMJ Open's open peer review process please email info.bmjopen@bmj.com

BMJ Open

Factors associated with reporting lacking interest in sex and their interaction with gender: Findings from the third British National Survey of Sexual Attitudes and Lifestyles

Journal:	*BMJ Open*
Manuscript ID	bmjopen-2017-016942
Article Type:	Research
Date Submitted by the Author:	20-Mar-2017

Complete List of Authors:	Graham, Cynthia; University of Southampton, Psychology<br>Mercer, Catherine; University College London, Research Department of Infection and Population Health<br>Tanton, Clare; University College London, Research Department of Infection & Population Health<br>Jones, Kyle; University College London, Research Department of Infection and Population Health<br>Johnson, Anne; University College London, Research Department of Infection and Population Health<br>Wellings, Kaye; London School of Hygiene and Tropical Medicine,<br>Mitchell, Kirstin; London School of Hygiene and Tropical Medicine

**Primary Subject Heading**:	Sexual health
Secondary Subject Heading:	Epidemiology
Keywords:	SEXUAL MEDICINE, EPIDEMIOLOGY, Sexual and gender disorders < PSYCHIATRY

SCHOLARONE™
Manuscripts

For peer review only

Factors associated with reporting lacking interest in sex and their interaction with gender: Findings from the third British National Survey of Sexual Attitudes and Lifestyles

Graham, C. A.,[1] Mercer, C. H.,[2] Tanton, C. [2] Jones, K. G.,[2] Johnson, A. M.,[2] Wellings, K.,[3] Mitchell, K. R. [3,4]

[1]Centre for Sexual Health Research, Department of Psychology, University of Southampton, Southampton, U.K.
[2]Centre for Sexual Health and HIV Research, Research Department of Infection & Population Health, University College London, London, U.K.
[3]Centre for Sexual and Reproductive Health Research, Department of Social and Environmental Health Research, London School of Hygiene and Tropical Medicine, London, U.K.
[4]MRC/CSO Social and Public Health Sciences Unit, University of Glasgow, 200 Renfield Street, Glasgow G2 3QB, U.K.

Corresponding author:
Cynthia A. Graham
Department of Psychology
Faculty of Social, Human, and Mathematical Sciences
Shackleton Building (B44)
University of Southampton
Highfield, Southampton
SO17 1BJ UK
tel: 023 8059 3091
e-mail: C.A.Graham@soton.ac.uk

Word count: 3,296

For peer review only

ABSTRACT

Objectives: To investigate factors associated with reporting lacking interest in sex, and how these vary by gender.

Setting: British general population.

Design: Complex survey analyses of data collected for a cross-sectional probability sample survey, undertaken 2010-12, specifically logistic regression to calculate age-adjusted odds ratios (AOR) to identify associated factors.

Participants: 4,839 men and 6,669 women aged 16-74 years who reported >=1 sexual partner (opposite-sex or same-sex) in the past year for the third National Survey of Sexual Attitudes and Lifestyles [Natsal-3].

Main outcome measure: Lacking interest in sex for >=3 months in the past year.

Results: Overall, 15.0% (13.9-16.2) of men and 34.2% (32.8-35.5) of women reported lacking interest in sex. This was associated with age and physical and mental health for both men and women, including self-reported general health and current depression. Lacking interest in sex was more prevalent among men and women reporting STI diagnoses (ever), non-volitional sex (ever), and holding sexual attitudes related to normative expectations about sex. Some gender similarities in associated relationship and family-related factors were evident, including partner having had sexual difficulties in the last year (M: AOR:1.41 [1.07-1.86]; W: AOR:1.60 [1.32-1.94]), not feeling emotionally close to partner during sex (M: 3.74 [1.76-7.93]; W: 4.80 [2.99-7.69], and ease of talking about sex (M: 1.53 [1.23-1.90] W: 2.06 [1.77-2.39]). Among women only, lack of interest in sex was higher among those in a relationship of >1 year in duration and those not sharing the same level of interest (4.57 [3.87-5.38]) or preferences (2.91 [2.22-3.83]) with a partner.

Conclusions: Both gender similarities and differences were found in factors associated with lacking interest in sex, with the most marked differences in relation to some relationship variables. Findings highlight the need to assess, and if appropriate, treat lacking interest in sex in a holistic and relationship-specific way.

For peer review only

ARTICLE SUMMARY

Strengths and limitations of this study

- This study used nationally representative data to investigate factors associated with reporting lacking interest in sex, and how these vary by gender, in the British population.
- The study included detailed assessment of a range of relationship context and attitudinal variables seldom included in previous population-based surveys.
- Information about lacking interest in sex was assessed with a single item, asking participants whether they had lacked interest in having sex for a period of three months or more in the past year. Those who reported this were also asked whether they experienced associated distress.
- The cross-sectional data do not allow us to establish the causal direction of associations between lacking interest in sex and factors.

For peer review only

In Britain's third National Survey of Sexual Attitudes and Lifestyles (Natsal-3) lacking interest in sex was the most common sexual difficulty reported by both men and women (1). It was more frequently reported by women than by men; 34.2% of women compared with 14.9% of men stated that they had lacked interest in sex for 3 months or more in the past year. When duration and symptom severity criteria are considered (i.e., that symptoms last six months or more and occur "very often" or "always") these prevalence estimates are much lower (2), but the gender difference is maintained.

Researchers have paid more attention to problems of low sexual interest in women than in men (3-5). Among men the predominant focus has been on erectile functioning and on physiological causes of lacking interest in sex such as hormonal status, rather than on psychosocial determinants. This lack of attention to male problems is reflected in recent revisions to the Diagnostic and Statistical Manual (DSM-5) classification of sexual disorders (6) which involved major changes to sexual arousal and desire disorder categories in women, but no substantive changes for male disorders.

Most but not all studies involving men have reported an association between low sexual interest and increasing age (for review, see 7). However, there are conflicting findings on the association with physical and mental health (8,9). Limited research suggests that psychosocial and relationship factors may also be associated with low sexual desire in men (8, 10-12).

Among women, factors that have been consistently associated with lacking interest in sex are relationship problems, relationship quality, and partner's sexual functioning (13-17), poor physical health (18), and negative mood states/depression

For peer review only

(13, 18,19). There are inconsistent findings on the association between low sexual interest and both age and menopausal status (14,18). Few large-scale surveys have examined possible links between lacking interest in sex and sexual attitudes and lifestyles.

Studies have, for the most part, used small, clinical samples of patients seeking treatment for low sexual desire problems. The potential for bias in such studies is revealed in previously reported findings from Natsal-3 that only 14.4% of men and 16.6% of women with sexual function problems had sought help or advice (1). The few large-scale probability-based surveys involving both men and women have focused on associations between low sexual desire and sociodemographic factors. In summary, the evidence on the factors associated with men's and women's reports of low sexual desire is drawn largely from non-representative samples, is somewhat equivocal and, in men, sparse.

The research questions addressed in this paper are: What sociodemographic, relationship, sexual behaviour, and sexual attitudinal factors are associated with lacking interest in sex in sexually active men and women?; and to what extent do these factors vary by gender?

METHOD

Participants and Procedure

Natsal-3 is a probability sample survey of 15,162 men and women aged 16-74 years in Britain, interviewed between September 2010 and August 2012. A multi-stage, clustered, and stratified probability sample design was used and participants were interviewed using a combination of computer-assisted personal interviews (CAPI), and computer-assisted self-interviews (CASI). After weighting to adjust for

For peer review only

unequal probabilities of selection and to match the British population in terms of age, gender and geographical region, the Natsal-3 sample was broadly representative, on key variables, of the British population as described by the 2011 Census (20).

The estimated response rate was 57.7%, and the estimated cooperation rate (the number of interviews completed from eligible addresses for which contact was made) was 65.8% (of all eligible addressed contacted) (21). Fuller details of the survey methodology and sample characteristics are published elsewhere (20, 21) and for demographic characteristics of the sample, see (21). Participants provided oral informed consent for interviews and the survey was approved by the NRES Committee South-Central – Oxford A (Ref.: 10/H0604/27).

Outcome Measures

In the CASI, participants who reported at least one sexual partner in the past year (hereon 'sexually active participants') were asked whether they had lacked interest in having sex for a period of three months or more in the past year. We used selected items from the Natsal-SF, a newly developed and validated measure of sexual function comprising questions about problems with sexual response, relational aspects of sexual function, and self-appraisal of sex life (22, 23).

Statistical Analysis

All analyses were done using the complex survey functions of STATA (version 14; StataCorp LP, College Station, Texas) to account for the weighting, clustering, and stratification of the data. We used multivariable logistic regression to calculate age-adjusted odds ratios (AOR) to examine the associations between reports of lacking interest in sex lasting three months or longer in the past year, and sociodemographic, health, relationship, sexual behaviour, and sexual attitude variables. For each variable,

For peer review only

we also tested the interaction between gender, to see if the magnitude of the associations between the above factors and reports of lacking interest in sex was the same for men and women. We conducted a sensitivity analysis for the outcome variable reporting lack of interest in sex lasting three months or longer *and* distress about this symptom to assess whether similar associations were found. We also examined the association between reporting lacking interest in sex and the other sexual function problems asked about in Natsal-3, using AORs.

RESULTS

Overall, 15.0 (95% CI 13.9-16.2) of sexually active men and 34.2% (95% CI 32.8-35.5) of sexually active women reported lacking interest in sex for three months or longer in the year prior to interview. Table 1 presents the associations between lacking interest in sex and sociodemographic, health, relationship, sexual behaviour, and sexual attitudinal variables for men and women.

Age was significantly associated with lacking interest in sex. Prevalence increased with age, being lowest among younger participants (16-24 years; M: 11.5%; W: 24.8%) and peaking in men aged 35-44 years (17.2%) and in women aged 55-64 years (38.8%). Regarding demographic variables, after adjusting for age, lack of interest was associated with leaving school at 16 (men only; AOR: 1.31), being unemployed (men only; AOR: 1.44), and less frequent religious practice (women only; AOR: 0.79).

After adjusting for age, there were associations between all physical and mental health variables assessed and lacking interest in sex. Individuals in poorer health (AORs: M: 3.29; W: 1.93), those who had difficulty walking upstairs (AORs: M: 1.8; W: 1.15), those with a longstanding medical condition (AORs: M 1.76; W: 1.35),

For peer review only

and those who had screened positive for current depression (AORs: M: 2.95; W: 2.79) or who had been treated for depression in the past year (AORs: M: 2.82; W: 2.32) were more likely to report lacking interest in sex. The magnitude of these associations was similar for men and women. Menopausal status in women and circumcision in men were not associated with the likelihood of lacking sexual interest.

Regarding sexual behaviour, among both men and women, lack of interest was associated with frequency of sexual activity (defined as vaginal, oral or anal intercourse) in the four weeks prior to interview; 12.4% of men, and 33.8% of women who reported having engaged in at least 3 sexual acts reported lack of interest, vs. 20.7% of men and 42.9% of women who reported no sexual activity. Associations with recent masturbation differed by gender; lack of interest in sex was *more* common among men who reported having recently masturbated but *less* common among women who did so. Women with two or more partners in the past year were less likely to report low sexual interest than those with only one partner (AOR: 0.70) but this association was not seen among men.

Associations were found between lacking interest in sex and several relationship contextual variables and for many of these variables associations were stronger for women than for men. For both men and women, lack of interest was associated with relationship status, although the association was stronger among women than men. Compared to those living with a partner, men and women in a steady relationship but not living together, and women not in a steady relationship were less likely to report lacking interest in sex. Longer duration of most recent sexual relationship was significantly associated with lacking interest in sex only among women, increasing with longer relationship duration. Having been pregnant in the last

For peer review only

year was associated with lacking sexual interest as was having one or more young child(ren).

Among both men and women, there was an association between ease of communication and lacking interest in sex. Those who found it “always easy to talk about sex” with their partner were less likely to report low interest. Lack of interest was more likely among those whose partner had sexual difficulties in the last year, and those who reported a lower assessment of happiness with the relationship, and not feeling emotionally close to partner during sex. Among women but not men, not sharing the same level of sexual interest with a partner, and not sharing the same sexual likes and dislikes, were also associated.

Lack of interest in sex was significantly associated with a range of sexual health indicators, including sexual competence at first sex and previous STI diagnosis. The strength and direction of associations was similar for men and women, except for reporting another sexual function problem, which was significant for two or more problems in men, but one or more problems in women.

Regarding attitudinal variables, both men and women who endorsed statements that “people are under pressure to have sex” and “people want less sex as they age” were more likely to report lacking interest in sex over the past year. The only attitudinal variable that showed a significant interaction with gender was the one related to men having a “naturally higher sex drive than women.” Men who agreed with this statement were *less* likely than those who disagreed to lack interest in sex, while the reverse was true among women.

Table 2 (online supplementary file) presents the associations between lacking interest in sex and being distressed about this (as a measure/marker of severity), and

For peer review only

the above sociodemographic, health, and sexual relationship/behaviour variables. While prevalence was lower, the associations and the interactions with gender were generally similar; however, some of the previous gender-specific associations with sociodemographic variables (e.g., Index of Multiple Deprivation and education) were no longer significant when the outcome variable was reported low sexual interest *and* associated distress.

Regarding the association between reporting lacking interest in sex and the other sexual function problems asked about in Natsal-3, the strongest associations were for lacking enjoyment in sex (AORs=9.78 and 8.95 for men and women, respectively), followed by feeling no excitement or arousal during sex (AORs=9.21 and 9.16 for men and women, respectively) (see Table 3).

DISCUSSION

We identified a broad range of factors, including some that have not been explored in previous large-scale surveys, that were associated with men's and women's reports of lacking interest in sex in a representative British population-based survey. Our findings, discussed below, revealed some gender similarities as well as some interesting gender differences. The strongest evidence for gender differences was for the relationship context variables, where associations with lacking interest in sex were much stronger for women than for men.

Interpretation of findings in context of previous research

Our finding relating to differences by age is consistent with some, but not all, results from previous research which has yielded generally inconsistent findings. Some studies have, like ours, shown a higher prevalence of sexual interest problems in older than in younger women (24-26). Others have found no association between age and

For peer review only

low sexual interest complaints (14, 27) and yet more have shown lack of sexual interest to be more common among younger women (18). Whereas we found a marginal relationship with age in men, some studies (though not all e.g., 28) have found a stronger relationship (12, 29). It is possible that the varied findings might in part be a result of varied definitions of low sexual interest or samples with different age ranges.

The finding in this analysis, that having young children appears to increase the likelihood of reporting lack of sexual interest for women, but not for men, remains unchanged since the previous Natsal-2 survey (30). This may be due to fatigue associated with a primary caring role (31), the fact that daily stress appears to affect sexual functioning in women more than men (32), or possibly a shift in focus of attention attendant on bringing up small children.

The finding of a link between lacking interest in sex and lacking enjoyment in sex and/or feeling no excitement or arousal during sex is not surprising and has been shown in previous studies (3). The strong associations between lack of interest in sex and physical and mental health indicators, which we observed for both men and women, is not entirely consistent with findings from other studies. While this link has been persuasively shown for women (13, 18, 19), in men, the evidence is more equivocal. In a study of men attending an outpatient clinic for sexual problems, psychological symptoms such as anxiety and depression were more predictive of low sexual desire than hormonal or other physical markers (11). In contrast, DeRogatis et al. (9), in their study of men with erectile dysfunction, observed no differences in depressive symptoms, concurrent illness, or medication use between men with and without symptoms of low sexual desire.

For peer review only

The gender differences in associations between masturbation and a lack of sexual interest are interesting and have been explored in few previous population-based studies. Our observation that lack of interest was *more* commonly reported by men who had recently masturbated, but *less* commonly reported by women who had done so may reflect a tendency among women for self-pleasuring to be, not a substitute for partnered sex but instead a part of a broader repertoire of sexual fulfilment; this possibility is worthy of further exploration. In contrast, for men frequency of masturbation reflects reduced frequency of partnered sex (33). However, it is worth noting that in the U.S. National Health and Social Life Survey, lifetime number of sexual partners and masturbation practices were unrelated to the likelihood of sexual desire difficulties for either men or women (34).

Our observation that duration of most recent sexual relationship showed a strong association with lacking interest in sex in women is consistent with previous studies (15, 17). There has been little comparable research among men with which to corroborate the absence of such an association among men in our analysis.

Our data confirm the importance of the relational context in individuals' level of sexual interest. The strong associations between relationship and partner factors and sexual interest are consistent with those shown in many previous studies relating to women (13-17) and with a much smaller literature in men (35,36). In particular, sexual dysfunction in a male partner has previously been associated with women's levels of sexual desire (15, 37, 38), and sexual desire discrepancy in couples has been linked to lower reported relationship satisfaction and more couple conflict (39).

The strong links found between several key sexual health outcomes and lack of interest in sex are interesting; among both men and women, reporting an STI

For peer review only - http://bmjopen.bmj.com/site/about/guidelines.xhtml

diagnosis, and non-volitional sex were associated with reporting lack of interest in sex. Our finding that lacking "sexual competence" at first sexual intercourse was linked with subsequent lack of interest in sex among women but not men, may reflect a greater salience of contextual aspects of first sex for women. More women than men report being pressured by a partner on the first occasion of heterosexual intercourse, and to have subsequently experienced regret about first sexual experiences (40). These findings suggest that for women, early sexual experiences may shape future sexual encounters/relationships to a greater extent than for men.

To our knowledge no previous studies have assessed the association between attitudes toward sexual matters and lack of interest in sex. Endorsing the assumption that "people want less sex as they age" was associated with lack of interest in both genders. It might be that this belief contributes to a decline in interest, or – equally plausible – that those who lack interest adopt this attitude to avoid viewing their experience as problematic. Interestingly, men who endorsed the view that "men have a higher sex drive than women" were significantly *less* likely to report lacking interest in sex, whereas women who agreed with this statement were *more* likely to do so. If people responded to this statement with reference to their own relationship, these findings may be seen as making intuitive sense. The results suggest that endorsing stereotypical gender-norms related to sex may adversely affect women more than men.

Strengths and Limitations

Strengths of our study include the use of national probability sample survey data involving both men and women across a wide age range (20, 21). With a few exceptions (e.g., 12, 14, 28, 41), most surveys on sexual desire problems have sampled

For peer review only

either men *or* women, precluding direct comparisons within the same sample. Another strength was the detailed and holistic examination of relationship context and attitudinal variables, which few previous studies have reported. Response rates for Natsal-3 were also similar to those of other major social surveys in Britain (42) and higher than many previous surveys of sexual problems (34, 43).

Limitations include the cross-sectional nature of the data, which mean that we are unable to infer temporality and causality. We only used a single item to assess lacking interest in sex, although we additionally took account of whether those who reported this also reported that it caused them distress, as a way of trying to capture more problematic lack of interest. It is important to acknowledge, however, that these data do not necessarily correspond to clinical diagnoses. This sensitivity analysis enabled us to demonstrate that similar associations exist regardless of whether or not distress was reported.

Implications for Research and Practice

Our findings underscore the importance of the relational context in understanding low sexual interest in both men and women. For women in particular, the experience of sexual interest appears strongly linked with their perceptions of the quality of their relationships, their communication with partners, and their expectations/attitudes about sex. In the context of the recent FDA approval of flibanserin, the first drug to treat low sexual desire in women (44), these findings are relevant to the current debate about whether striving for a pharmaceutical solution to women’s sexual desire problems is an appropriate and feasible goal (45, 46). Some authors have suggested that women with complaints of low sexual interest might

For peer review only

benefit most from integrative approaches that accord with a biopsychosocial model (47).

Our findings on the strong association between open sexual communication (i.e., "finding it always easy to talk about sex") and a reduced likelihood of reporting lack of interest in sex, particularly for women, emphasise the importance of providing a broad sexual and relationships education, rather than limiting attention only to adverse consequences of sex and how to prevent them. Similarly, the important role of early sexual experiences, and sexual "competence," especially for women, in shaping later experiences of sexual desire supports the need for comprehensive sex education.

In a clinical context, our findings emphasise the importance of health care professionals assessing psychological and interpersonal variables in individuals presenting with complaints of low sexual interest (48). In couple therapy, it is important that therapists have an awareness of the differences between men and women in the factors associated with low sexual interest. Lastly, our findings support previous research on the critical role of physical and mental health in understanding low sexual interest problems experienced by men and women (11, 18).

Conclusions

This study extends our understanding of the factors associated with lack of interest in sex in men and women, the gender similarities and differences, and highlights the need to assess and treat sexual desire problems in a holistic and relationship- as well as gender-specific way.

For peer review only

Competing interests statement

AMJ has been a Governor of the Wellcome Trust since 2011. The remaining authors have nothing to disclose. Completed disclosure of interests form available to view online as supporting information.

Contribution to authorship

The paper was conceived by CAG, CHM, AMJ, KW, and KRM. CAG wrote the first draft, with further contributions from all authors. Statistical analyses were undertaken by CHM, CT, and KGJ. CHM, AMJ (Principal Investigator) and KW, initial applicants on Natsal-3, wrote the study protocol and obtained funding. Natsal-3 questionnaire design, ethics applications, and piloting were undertaken by CHM, CT, AMJ, KW, and KRM. Data management was undertaken by NatCen Social Research, UCL and LSHTM. All authors contributed to data interpretation, reviewed successive drafts and approved the final version of the manuscript.

Details of ethics approval

Natsal-3 was approved by the NRES Committee South Central-Oxford A (Ref: 10/H0604/27) on 12 July 2010. Participants provided oral informed consent for interviews.

Funding statement

Natsal-3 was supported by grants from the U.K. Medical Research Council (G0701757) and the Wellcome Trust (084840), with support from the Economic and Social Research Council and the Department of Health. Since September 2015. KRM has been core funded by the UK Medical Research Council through the MRC/CSO Social & Public Health Sciences Unit, University of Glasgow [MC_UU_12017-11].

Data sharing statement

The Natsal-3 dataset is publicly available from the UK Data Service: https://discover.ukdataservice.ac.uk/; SN: 7799; persistent identifier: 10.5255/UKDA-SN-77991-1.

For peer review only

REFERENCES

1. Mitchell KR, Mercer CH, Ploubidis GB, et al. Sexual function in Britain: Findings from the third National Survey of Sexual Attitudes and Lifestyles (Natsal-3). *Lancet* 2013;382:1817-1829. doi 10.1016/S0140-6736(13)62366-1 [published Online First: 26 November 2013].

2. Mitchell KR, Jones KG, Wellings K, et al. Estimating the prevalence of sexual function problems: The impact of morbidity criteria. *J Sex Res* 2016;53:55-967. doi 10.1080/00224499.2015.1089214 [published Online First: 25 November 2015].

3. Brotto LA. The DSM diagnostic criteria for hypoactive sexual desire disorder in women. *Arch Sex Behav* 2010a; 39:221-239. doi 10.1007/s10508-009-9543-1 [published Online First: 24 September 2009].

4. Carvalho J, Nobre P. Biopsychosocial determinants of men's sexual desire: testing an integrative model. *J Sex Med* 2011;8:754-763. doi 10.1111/j.1743-6109.2010.02156.x [published Online First: 22 December 2010].

5. Štulhofer A, Carvalheira AA, Træen B. Is responsive sexual desire for partnered sex problematic among men? Insights from a two-country study. *Sex Relation Ther* 2013;28:246-258. doi 10.1080/14681994.2012.756137 [published Online First: 21 December 2012].

6. American Psychiatric Association. *Diagnostic and statistical manual of mental disorders*, 5th. ed. Arlington, VA: Author 2013.

7. Brotto LA. The DSM diagnostic criteria for hypoactive sexual desire disorder in men. *J Sex Med* 2010b;7:2015-2030. doi 10.1111/j.1743-6109.2010.01860.x [published Online First: 1 June 2010].

8. Corona G, Lee DM, Forti G, et al. Age-related changes in general and sexual health in middle-aged and older men: Results from the European Male Ageing Study (EMAS). *J Sex Med* 2010;7:1362-1380. doi 10.1111/j.1743-6109.2009.01601.x [published Online First: 19 November 2009].

9. DeRogatis L, Rosen RC, Goldstein I, et al. Characterization of hypoactive sexual desire disorder (HSDD) in men. *J Sex Med* 2012;9:812-820. doi 10.1111/j.1743-6109.2011.02592.x [published Online First: 12 January 2012].

10. Carvalheira A, Træen B, Štulhofer A. Correlates of men's sexual interest: a cross-cultural study. *J Sex Med* 2014;11:154-164. doi 10.1111/jsm.12345 [published Online First: 28 October 2013].

11. Corona G, Petrone L, Mannucci E, et al. The impotent couple: low desire. *Int J Androl* 2005;28:46-52. doi 10.1111/j.1365-2605.2005.00594.x [published Online First: 18 October. 2005].

12. DeLamater JD, Sill M. Sexual desire in later life. *J Sex Res* 2005;42:138-149. doi 10.1080/00224490509552267 [published Online First: 11 January 2010].

13. Dennerstein L, Koochaki P, Barton I, et al. Hypoactive sexual desire disorder in menopausal women: a survey of Western European women. *J Sex Med* 2006;3:212-222. doi 10.1111/j.1743-6109.2006.00215.x [published Online First: 20 February 2006].

14. Hayes RD, Dennerstein L, Bennett CM, et al. Risk factors for female sexual dysfunction in the general population: Exploring factors associated with low sexual function and sexual distress. *J Sex Med* 2008;5:1681-1693. doi 10.1111/j.1743-6109.2008.00838.x [published Online First: 16 December 2015].

For peer review only

15. McCabe MP, Goldhammer DL. Demographic and psychological factors related to sexual desire among heterosexual women in a relationship. *J Sex Res* 2012;49:78-87. doi 10.1080/00224499.2011.569975 [published Online First: 28 April 2011].

16. Öberg K, Sjögren Fugl-Meyer K. On Swedish women's distressing sexual dysfunctions: some concomitant conditions and life satisfaction. *J Sex Med* 2005;2:169-180. doi 10.1111/j.1743-6109.2005.20226.x [published Online First: 17 February 2005].

17. Witting K, Santtila P, Varjonen M, et al. Female sexual dysfunction, sexual distress, and compatibility with partner. *J Sex Med* 2008;5:2587-2599. doi 10.1111/j.1743-6109.2008.00984.x [published Online First: 27 August 2008]

18. Shifren JL, Monz BU, Russo PA, et al. Sexual problems and distress in United States women. *Obstet Gynecol* 2008;112:970-978. doi 10.1097/AOG.0b013e3181898cdb

19. Johannes CB, Clayton AH, Odom DM, et al. Distressing sexual problems in United States women revisited: prevalence after accounting for depression. *J Clin Psychiatry* 2009;70:1698-1706. doi 10.4088/JCP.09m05390gry

20. Erens B, Phelps A, Clifton S, et al. Methodology of the third British National Survey of Sexual Attitudes and Lifestyles (Natsal-3). *Sex Transm Infect* 2014;90:84-89. doi 10.1136/sextrans-2013-051359 [published Online First: 25 November 2013].

21. Mercer CH, Tanton C, Prah P, et al. Changes in sexual attitudes and lifestyles in Britain through the life course and over time: findings from the National Surveys of Sexual Attitudes and Lifestyles (Natsal). *Lancet* 2013;382:1781-1794.

For peer review only

doi 10.1016/S0140-6736(13)62035-8 [published Online First: 26 November 2013].

22. Jones KG, Mitchell KR, Ploubidis GB, et al. The Natsal-SF measure of sexual function: Comparison of three scoring methods. *J Sex Res* 2015;52:640-646. doi 10.1080/00224499.2014.985813 [published Online First: 10 February 2015].

23. Mitchell KR, Ploubidis GB, Datta J, et al. The Natsal-SF: a validated measure of sexual function for use in community surveys. *Eur J Epidemiol* 2012;27:409-18. doi 10.1007/s10654-012-9697-3 [published Online First: 19 June 2012].

24. Abdo CHN, Oliveira WM, Moreira ED, et al. Prevalence of sexual dysfunctions and correlated conditions in a sample of Brazilian women—results of the Brazilian study on sexual behavior (BSSB). *Int J Impot Res* 2004;16:160-166. doi 10.1038/sj.ijir.3901198 [published Online First: 12 February 2004].

25. Hayes RD, Dennerstein L, Bennett CM, et al. Relationship between hypoactive sexual desire disorder and aging. *Fertil Steril* 2007;87:107-112. doi 10.1016/j.fertnstert.2006.05.071 [published Online First: 1 November 2006].

26. Peixoto MM, Nobre P. Prevalence and sociodemographic predictors of sexual problems in Portugal: A population-based study with women aged 18 to 79 years. *J Sex Marital Ther* 2015;41:169-180. doi 10.1080/0092623X.2013.842195 [published Online First: 23 December 2013].

27. Rosen RC, Shifren JL, Monz BU, et al. Correlates of sexually-related personal distress in women with low sexual desire. *J Sex Med* 2009;6:1549-1560. doi 10.1111/j.1743-6109.2009.01252.x [published Online First: 30 March 2009].

28. Laumann EO, Glasser DB, Neves RCS, et al. A population-based survey of sexual activity, sexual problems and associated help-seeking behavior patterns in

For peer review only

mature adults in the United States of America. *Int J Impot Res* 2009;21:171-178. doi 10.1038/ijir.2009.7 [published Online First: 26 February 2009].

29. Eplov L, Giraldi A, Davidsen M, et al. Sexual desire in a nationally representative Danish population. *J Sex Med* 2007;4:47-56. doi 10.1111/j.1743-6109.2006.00396.x [published Online First: 3 January 2007].

30. Mercer CH, Fenton KA, Johnson AM, et al. Who reports sexual function problems? Empirical evidence from Britain's 2000 National Survey of Sexual Attitudes and Lifestyles. *Sex Transm Infect* 2005;81:394-399. doi 10.1136/sti.2005.0151 49 [published Online First: 30 September 2005].

31. Park A, Bryson C, Clery E, et al. *British social attitudes 30*. London: NatCen 2013.

32. Bodenmann G, Ledermann T, Blattner D, et al. Associations among everyday stress, critical life events, and sexual problems. *J of Nerv Ment Dis* 2006;194:494-501. doi 10.1097/01.nmd.0000228504.15569.b6

33. Gerressu M, Mercer CH, Graham CA, et al. Prevalence of masturbation of associated factors in a British national probability survey. *Arch Sex Behav* 2008;37:266-278. doi 10.1007/s10508-006-9123-6 [published Online First: 27 February 2007].

34. Laumann EO, Paik A, Rosen RC. Sexual dysfunctions in the United States: Prevalence and predictors. *JAMA* 1999;281:537-544. doi 10.1001/jama.281.6.537

35. Janssen E, McBride KR, Yarber W, et al. Factors that influence sexual arousal in men: A focus group study. *Arch Sex Behav* 2008;37:252-265. doi 10.1007/s10508-007-9245-5 [published Online First: 27 November 2007].

For peer review only

36. Murray S, Milhausen RR, Graham CA, et al. A qualitative exploration of factors that affect sexual desire among men aged 30 to 65 in long-term relationships. *J Sex Res* 2016;doi 10.1080/00224499.2016.1168352 doi 10.1080/00224499.2016.1168352 [published Online First: 2 May 2016].

37. Çayan S, Bozlu M, Canpolat B, et al. The assessment of sexual functions in women with male partners complaining of erectile dysfunction: does treatment of male sexual dysfunction improve female partner's sexual functions? *J Sex Marital Ther* 2004;30:333-341. doi 10.1080/00926230490465091 [published Online First: 12 August 2010].

38. Fisher WA, Rosen RC, Eardley I, et al. Experience of female partners of men with erectile dysfunction: The female experience of men's attitudes to life events and sexuality (FEMALES) study. *J Sex Med* 2005;2:675-668. doi 10.1111/j.1743-6109.2005.00118.x [published Online First: 16 August 2005].

39. Willoughby BJ, Farero AM, Busby DM. Exploring the effects of sexual desire discrepancy among married couples. *Arch Sex Behav* 2014;43:551-562. doi 10.1007/s10508-013-0181-2 [published Online First: 18 September 2013].

40. Hawes ZC, Wellings K, Stephenson J. First heterosexual intercourse in the United Kingdom: A review of the literature. *J Sex Res* 2010;47:137-152. doi 10.1080/00224490903509399 [published Online First: 30 March 2010].

41. Laumann EO, Nicolosi A, Glasser DB, et al. Sexual problems among women and men aged 40–80 years: Prevalence and correlates identified in the Global Study of Sexual Attitudes and Behaviors. *Int J Impot Res* 2005;17:39-57. doi 10.1038/sj.ijir.3901250 [published Online First: 24 June 2004].

42. Park A, Clery E, Curtice J, et al. *British social attitudes: The 28th report*. London,

For peer review only

England: NatCen 2012.

43. Christensen BS, Grønbæk M, Osler M, et al. Sexual dysfunctions and difficulties in Denmark: Prevalence and associated sociodemographic factors. *Arch Sex Behav* 2011;40:121-132. doi 10.1007/s10508-010-9599-y [published Online First: 19 February 2010].

44. Woloshin S, Schwartz LM. US Food and Drug Administration approval of flibanserin: Even the Score does not add up. *JAMA Intern Med* 2016; doi: 10.1001/jamainternmed.2016.0073 [published Online First: 29 February 2016].

45. Graham CA, Boynton PM, Gould K. Women's sexual desire: Challenging narratives of dysfunction. *Eur Psychol*, in press.

46. Hart G, Wellings K. Sexual behaviour and its medicalisation: In sickness and in health. BMJ 2002;324:896-900. doi 10.1136/bmj.324.7342.896 [published Online First: 13 April 2002].

47. Frühauf S, Gerger H, Schmidt HM, et al. Efficacy of psychological interventions for sexual dysfunction: a systematic review and meta-analysis. *Arch Sex Behav* 2013;42:915-933. doi 10.1136/bmj.324.7342.896 [published Online First: 13 April 2002].

48. Brotto LA, Atallah CS, Johnson-Agbakwu C, et al. Psychological and interpersonal dimensions of sexual function and dysfunction. *J Sex Med* 2016;13:538-571. doi 10.1016/j.jsxm.2016.01.019 [published Online First: 25 March 2016].

49. Payne RA, Abel GA. UK indices of multiple deprivation-a way to make comparisons across constituent countries easier. *Health Stat Q* 2012;53:22-37. Retrieved from https://search.proquest.com/docview/1054966975?accountid=13963

For peer review only

Table 1: Factors associated with lacking interest in having sex for at least 3 months in the past year in sexually active men and women

	Men						Women						
	Denom. (unwt, wt)	%	(95%CI)	Age-adjusted OR	(95%CI)	p-value	*Denom. (unwt, wt)*	%	(95%CI)	Age-adjusted OR	(95%CI)	p-value	*p-value for interaction with sex*[a]
**All**	*4839, 5973*	**15.0%**	**(13.9-16.2)**				*6669, 5755*	**34.2%**	**(32.8-35.5)**				
**Socio-demographics**													
**Age group**						0.0471						<0.0001	*0.6733*
16-24	*1279, 936*	11.5%	(9.4-14.0)	1	-		*1662, 923*	24.8%	(22.5-27.1)	1	-		
25-34	*1376, 1238*	14.6%	(12.7-16.6)	1.32	(1.00-1.73)		*2236, 1246*	31.9%	(29.8-34.1)	1.42	(1.22-1.66)		
35-44	*719, 1298*	17.2%	(14.5-20.4)	1.61	(1.19-2.18)		*1050, 1290*	36.8%	(33.7-40.1)	1.77	(1.48-2.13)		
45-54	*630, 1186*	15.3%	(12.5-18.7)	1.40	(1.01-1.95)		*871, 1186*	37.9%	(34.5-41.5)	1.86	(1.53-2.25)		
55-64	*512, 849*	16.5%	(13.4-20.2)	1.53	(1.10-2.13)		*569, 755*	38.8%	(34.5-43.2)	1.92	(1.55-2.39)		
65-74	*323, 467*	13.9%	(10.4-18.3)	1.22	(0.81-1.82)		*281, 355*	34.2%	(28.4-40.5)	1.58	(1.18-2.12)		
**Index of multiple deprivation (quintiles)**[b]						0.093						0.0316	*0.0111*
1 (least deprived)	*977, 1279*	13.9%	(11.6-16.6)	1	-		*1248, 1208*	35.7%	(32.6-38.9)	1	-		
2	*962, 1264*	13.0%	(10.8-15.6)	0.93	(0.69-1.25)		*1290, 1208*	33.6%	(30.6-36.7)	0.92	(0.76-1.13)		
3	*942, 1169*	18.0%	(15.2-21.2)	1.38	(1.04-1.85)		*1299, 1116*	30.1%	(27.2-33.2)	0.81	(0.66-0.99)		
4	*967, 1184*	15.3%	(12.8-18.3)	1.15	(0.86-1.55)		*1384, 1137*	35.9%	(33.0-39.0)	1.08	(0.89-1.30)		
5 (most deprived)	*991, 1077*	15.1%	(12.7-17.8)	1.14	(0.85-1.52)		*1448, 1086*	35.3%	(32.4-38.3)	1.06	(0.87-1.28)		
**Education level**[c]						0.0083						0.2453	0.2914
Left school aged 17+	*2862, 3464*	13.5%	(12.1-15.1)	1	-		*4150, 3406*	32.7%	(31.0-34.5)	1	-		
Left school at 16	*1873, 2437*	17.2%	(15.3-19.4)	1.31	(1.07-1.60)		*2409, 2287*	36.6%	(34.4-38.9)	1.08	(0.95-1.23)		
**Employment status**						0.0086						0.0003	*0.0766*
Employed	*3211, 4254*	14.7%	(13.3-16.1)	1	-		*3871, 3517*	34.6%	(32.9-36.4)	1	-		
Full-time education	*542, 431*	12.6%	(8.8-17.5)	0.98	(0.64-1.51)		*693, 423*	22.5%	(19.0-26.4)	0.70	(0.55-0.89)		
Unemployed	*707, 723*	19.6%	(16.3-23.4)	1.44	(1.12-1.86)		*1681, 1282*	36.1%	(33.4-39.0)	1.11	(0.96-1.28)		
Retired	*375, 562*	13.6%	(10.4-17.7)	0.75	(0.52-1.09)		*415, 524*	35.8%	(31.0-40.9)	0.75	(0.57-0.99)		
**Practises religion at least once a month**						0.1687						0.0082	*0.9966*
No	*4283, 5179*	15.3%	(14.1-16.6)	1	-		*5659, 4754*	34.8%	(33.3-36.3)	1	-		
Yes	*521, 748*	12.9%	(10.0-16.4)	0.81	(0.60-1.09)		*956, 945*	30.7%	(27.5-34.2)	0.79	(0.67-0.94)		

For peer review only

Table 1 cont.

	Men						Women						
	Denom. (unwt, wt)	%	(95%CI)	Age-adjusted OR	(95%CI)	p-value	Denom. (unwt, wt)	%	(95%CI)	Age-adjusted OR	(95%CI)	p-value	p-value for interaction with sex[a]
**Health**													
**Self-reported general health**						<0.0001						<0.0001	0.1890
Very good/Good	4123, 5055	13.4%	(12.2-14.6)	1	-		5683, 4851	32.3%	(30.9-33.8)	1	-		
Fair	580, 745	21.9%	(18.3-25.8)	1.8	(1.41-2.30)		780, 709	42.2%	(38.2-46.3)	1.45	(1.21-1.75)		
Bad/very bad	135, 171	33.9%	(25.3-43.6)	3.29	(2.14-5.06)		206, 195	49.9%	(42.2-57.7)	1.93	(1.40-2.67)		
**Difficulty walking up stairs because of a health problem**						<0.0001						0.0497	0.1179
No difficulty	4475, 5460	14.1%	(12.9-15.3)	1	-		6062, 5107	33.3%	(31.8-34.7)	1	-		
Some difficulty	278, 393	23.0%	(18.1-28.8)	1.8	(1.30-2.49)		450, 482	39.2%	(34.4-44.2)	1.15	(0.92-1.43)		
Much difficulty/unable to do this	86, 120	30.9%	(20.9-43.0)	2.68	(1.57-4.57)		157, 166	47.0%	(38.0-56.1)	1.55	(1.06-2.25)		
**Longstanding illness or disability**						<0.0001						<0.0001	0.1348
No	3585, 4259	12.8%	(11.6-14.2)	1	-		4843, 4026	31.6%	(30.0-33.2)	1	-		
Yes	1253, 1713	20.5%	(18.1-23.1)	1.76	(1.44-2.16)		1825, 1729	40.1%	(37.5-42.8)	1.35	(1.17-1.55)		
**Number of comorbid conditions[d]**						<0.0001						<0.0001	0.7951
0	3453, 3994	12.8%	(11.5-14.1)	1	-		4357, 3536	29.9%	(28.2-31.5)	1	-		
1	939, 1329	18.9%	(16.2-21.9)	1.64	(1.30-2.06)		1555, 1416	38.6%	(35.9-41.5)	1.42	(1.23-1.64)		
>=2	446, 650	21.0%	(17.0-25.6)	1.91	(1.41-2.60)		755, 802	45.1%	(41.2-49.1)	1.75	(1.45-2.13)		
**Depressive symptoms[e]**						<0.0001						<0.0001	0.6249
No	4383, 5471	13.5%	(12.4-14.8)	1	-		5885, 5149	31.7%	(30.2-33.1)	1	-		
Yes	449, 495	31.3%	(26.4-36.7)	2.95	(2.26-3.85)		780, 602	55.2%	(51.0-59.5)	2.79	(2.32-3.37)		
**Treated for depression in the past year**						<0.0001						<0.0001	0.2447
No	4524, 5630	14.0%	(12.9-15.2)	1	-		5770, 5040	31.7%	(30.2-33.2)	1	-		
Yes	313, 342	31.5%	(25.7-38.0)	2.82	(2.08-3.83)		897, 713	51.4%	(47.6-55.2)	2.32	(1.96-2.75)		

Table 1 cont.

For peer review only

	Men						Women						
	Denom. (unwt, wt)	%	(95%CI)	Age-adjusted OR	(95%CI)	p-value	*Denom. (unwt, wt)*	%	(95%CI)	Age-adjusted OR	(95%CI)	p-value	*p-value for interaction with sex[a]*
**Menopausal status**												0.9326	
Not menopausal							*5485, 4187*	32.3%	(30.9-33.8)	1	-		
Menopausal							*1167, 1548*	38.9%	(36.0-41.9)	0.99	(0.79-1.24)		
**Circumcised**						0.5951							
No	*3909, 4728*	15.1%	(13.8-16.4)	1	-								
Yes	*857, 1166*	14.5%	(12.0-17.4)	0.94	(0.73-1.20)								
**<u>Sexual behaviour</u>**													
**Number of occasions of sex, past 4 weeks**						<0.0001						<0.0001	*0.4778*
0	*1013, 1163*	20.7%	(17.8-23.8)	1	-		*1408, 1245*	42.9%	(39.9-45.9)	1	-		
1-2	*1160, 1566*	18.7%	(16.2-21.5)	0.89	(0.69-1.14)		*1481, 1373*	39.6%	(36.7-42.5)	0.89	(0.75-1.05)		
3-4	*870, 1168*	12.4%	(10.1-15.1)	0.54	(0.41-0.73)		*1240, 1130*	33.8%	(30.7-37.0)	0.7	(0.58-0.85)		
5+	*1617, 1869*	9.2%	(7.8-11.0)	0.39	(0.30-0.51)		*2078, 1655*	22.6%	(20.5-24.8)	0.41	(0.34-0.49)		
**Masturbation, past 4 weeks**						0.0458						0.0038	*0.0005*
No	*1297, 1828*	13.7%	(11.8-15.8)	1	-		*4032, 3612*	36.0%	(34.3-37.7)	1	-		
Yes	*3531, 4132*	15.6%	(14.2-17.0)	1.24	(1.00-1.52)		*2615, 2114*	30.8%	(28.7-33.0)	0.83	(0.73-0.94)		
**No. of sexual partners, past year[f]**						0.5348						0.0038	*0.0183*
1	*3573, 4824*	15.0%	(13.7-16.3)	1	-		*5440, 5012*	35.3%	(33.8-36.8)	1	-		
2	*539, 513*	16.2%	(12.9-20.3)	1.14	(0.86-1.52)		*570, 364*	28.2%	(23.9-32.8)	0.80	(0.63-1.01)		
3+	*718, 627*	13.6%	(11.1-16.6)	0.94	(0.72-1.22)		*642, 366*	24.8%	(21.0-29.0)	0.70	(0.56-0.88)		
**Paid for sex, past year**						0.7167							
No	*4774, 5896*	15.0%	(13.9-16.2)	1	-								
Yes	*64, 75*	13.4%	(6.8-24.7)	0.87	(0.41-1.84)								
**Ever taken drugs to assist sexual performance**						0.0175						0.0666	*0.8967*
No	*4188, 5180*	14.4%	(13.2-15.7)	1	-		*6478, 5624*	34.0%	(32.6-35.4)	1	-		
Yes	*636, 776*	19.0%	(15.7-22.8)	1.36	(1.06-1.76)		*184, 124*	40.0%	(32.0-48.5)	1.39	(0.98-1.96)		

Table 1 cont.

For peer review only

	Men						Women						
	Denom. (unwt, wt)	%	(95%CI)	Age-adjusted OR	(95%CI)	p-value	*Denom. (unwt, wt)*	%	(95%CI)	Age-adjusted OR	(95%CI)	p-value	*p-value for interaction with sex*[a]
**Relationship context**													
**Relationship status**						0.0383						<0.0001	*0.0001*
Living with partner	*2708, 4266*	15.5%	(14.1-17.1)	1	-		*3967, 4168*	37.9%	(36.3-39.7)	1	-		
In a steady relationship, not living together	*947, 760*	12.0%	(9.6-14.8)	0.76	(0.57-1.00)		*1360, 790*	22.6%	(20.2-25.2)	0.51	(0.43-0.60)		
Not in a steady relationship, but previously cohabited	*446, 388*	18.2%	(14.6-22.5)	1.22	(0.91-1.62)		*752, 462*	28.9%	(25.4-32.8)	0.68	(0.56-0.83)		
Not in a steady relationship, never cohabited	*727, 551*	12.4%	(9.9-15.5)	0.8	(0.58-1.09)		*580, 330*	21.3%	(17.6-25.5)	0.49	(0.38-0.63)		
**Duration of most recent sexual relationship**						0.494						<0.0001	*<0.0001*
1 year or less	*1462, 1260*	13.0%	(11.0-15.3)	1	-		*1597, 998*	21.5%	(19.1-24.1)	1	-		
Between 1 and 5 years	*1247, 1227*	15.3%	(13.2-17.7)	1.21	(0.94-1.55)		*1758, 1148*	28.5%	(26.1-31.0)	1.45	(1.20-1.76)		
Between 5 and 15 years	*1065, 1484*	14.9%	(12.6-17.5)	1.14	(0.86-1.50)		*1774, 1458*	39.8%	(37.2-42.4)	2.37	(1.96-2.86)		
Over 15 years	*1004, 1904*	16.1%	(13.9-18.7)	1.19	(0.87-1.63)		*1445, 2036*	40.0%	(37.3-42.7)	2.31	(1.84-2.91)		
**Always easy to talk about sex with partners**[g]						0.0001						<0.0001	*0.0182*
Yes	*1695, 1899*	11.5%	(9.7-13.5)	1	-		*1746, 1451*	22.6%	(20.4-25.1)	1	-		
No/other	*3122, 4048*	16.7%	(15.3-18.2)	1.53	(1.23-1.90)		*4907, 4289*	38.0%	(36.4-39.6)	2.06	(1.77-2.39)		
**Happy with relationship**[h]						<0.0001						<0.0001	*0.8679*
Yes	*1951, 2791*	12.6%	(11.0-14.4)	1	-		*2736, 2601*	31.5%	(29.5-33.6)	1	-		
Other	*995, 1430*	21.0%	(18.4-23.9)	1.85	(1.47-2.32)		*1640, 1617*	45.4%	(42.7-48.1)	1.79	(1.55-2.08)		
**Participant does not share same level of interest in sex as partner**						0.2339						<0.0001	*<0.0001*
No/other	*2270, 3233*	15.0%	(13.4-16.7)	1	-		*3211, 3064*	27.2%	(25.4-29.0)	1	-		
Yes	*676, 988*	17.1%	(14.2-20.4)	1.17	(0.90-1.51)		*1166, 1155*	62.5%	(59.2-65.7)	4.57	(3.87-5.38)		

Table 1 cont.

For peer review only

	Men						Women						
	Denom. (unwt, wt)	%	(95%CI)	Age-adjusted OR	(95%CI)	p-value	*Denom. (unwt, wt)*	%	(95%CI)	Age-adjusted OR	(95%CI)	p-value	*p-value for interaction with sex[a]*
**Participant does not share same sexual likes & dislikes as partner**						0.4188						<0.0001	*<0.0001*
No/other	*2650, 3803*	15.3%	(13.8-16.9)	1	-		*4079, 3908*	34.9%	(33.3-36.6)	1	-		
Yes	*296, 418*	17.3%	(13.0-22.5)	1.16	(0.81-1.66)		*297, 310*	61.0%	(54.6-67.2)	2.91	(2.22-3.83)		
**Partner experienced sexual difficulties in past year**						0.0136						<0.0001	*0.4140*
No/other	*2431, 3454*	14.6%	(13.1-16.2)	1	-		*3726, 3498*	34.8%	(33.1-36.6)	1	-		
Yes	*513, 763*	19.4%	(15.8-23.6)	1.41	(1.07-1.86)		*649, 719*	46.8%	(42.5-51.1)	1.60	(1.32-1.94)		
**Does not feel emotionally close to partner when have sex**						0.0006						<0.0001	*0.5972*
No/other	*2904, 4165*	15.1%	(13.7-16.6)	1	-		*4263, 4108*	35.9%	(34.3-37.6)	1	-		
Yes	*42, 56*	39.9%	(23.6-58.8)	3.74	(1.76-7.93)		*112, 109*	73.0%	(62.8-81.3)	4.80	(2.99-7.69)		
**<u>Lifestyle</u>**													
**1+ child(ren) aged <5 in household**						0.9088						<0.0001	*0.0216*
No, none	*4100, 5015*	15.2%	(13.9-16.5)	1	-		*4997, 4671*	33.1%	(31.6-34.6)	1	-		
Yes, 1+	*727, 941*	14.5%	(11.9-17.6)	0.98	(0.76-1.28)		*1664, 1074*	38.6%	(36.0-41.4)	1.55	(1.34-1.79)		
**Pregnant in the last year**												0.0114	
No							*4227, 4122*	36.2%	(34.6-37.9)	1	-		
Yes							*437, 273*	41.7%	(36.6-47.1)	1.36	(1.07-1.72)		
**Used hormonal contraceptive, past year**												0.05	
No							*3759, 3838*	34.8%	(33.1-36.5)	1	-		
Yes							*2806, 1831*	33.0%	(30.9-35.1)	1.15	(1.00-1.33)		
**<u>Sexual health indicators</u>**													
**Ever diagnosed with a sexually transmitted infection**						<0.0001						0.0004	*0.0651*
No (or only thrush)	*4147, 5127*	14.0%	(12.8-15.3)	1	-		*5455, 4861*	33.4%	(31.9-34.9)	1	-		
Yes (excluding thrush)	*677, 830*	21.4%	(18.1-25.0)	1.67	(1.33-2.10)		*1206, 888*	38.2%	(35.1-41.5)	1.32	(1.13-1.54)		

Table 1 cont.

For peer review only

For peer review only - http://bmjopen.bmj.com/site/about/guidelines.xhtml

	Men						Women						
	Denom. (unwt, wt)	%	(95%CI)	Age-adjusted OR	(95%CI)	p-value	*Denom. (unwt, wt)*	%	(95%CI)	Age-adjusted OR	(95%CI)	p-value	*p-value for interaction with sex*[a]
**Ever experienced non-volitional sex**						0.0010						<0.0001	*0.3164*
No	*4705, 5824*	14.7%	(13.6-16.0)	1	-		*5815, 5055*	32.8%	(31.4-34.2)	1	-		
Yes/Don't know	*133, 148*	26.1%	(18.9-34.9)	2.07	(1.34-3.18)		*848, 695*	44.3%	(40.5-48.3)	1.66	(1.40-1.97)		
**Sexual competence at first sex**[i]						0.0706						<0.0001	*0.1797*
Not competent	*2407, 3037*	16.2%	(14.6-17.9)	1	-		*3438, 2927*	37.6%	(35.7-39.5)	1	-		
Competent	*2302, 2784*	13.7%	(12.1-15.4)	0.84	(0.69-1.01)		*3097, 2716*	30.3%	(28.4-32.3)	0.73	(0.65-0.83)		
**Number of other sexual response problems experienced**[j]						<0.0001						<0.0001	*0.0015*
0	*3208, 3945*	11.7%	(10.5-13.1)	1	-		*4377, 3759*	25.3%	(23.8-26.9)	1	-		
1	*1061, 1350*	10.9%	(9.0-13.2)	0.91	(0.71-1.17)		*1217, 1087*	34.8%	(31.7-38.0)	1.55	(1.32-1.82)		
2+	*570, 678*	42.5%	(37.9-47.2)	5.58	(4.41-7.04)		*1075, 909*	69.8%	(66.5-72.9)	6.91	(5.82-8.21)		
**<u>Attitudes</u>**													
**People are under pressure to have sex**						0.0115						0.0001	*0.7970*
Else	*1799, 2264*	13.1%	(11.4-15.0)	1	-		*1851, 1570*	29.3%	(26.8-31.9)	1	-		
Strongly agree/agree	*3038, 3707*	16.2%	(14.7-17.8)	1.29	(1.06-1.57)		*4817, 4185*	36.0%	(34.4-37.6)	1.34	(1.16-1.54)		
**People want less sex as they age**						<0.0001						<0.0001	*0.9443*
Else	*2943, 3472*	11.4%	(10.2-12.8)	1	-		*4044, 3278*	27.8%	(26.2-29.4)	1	-		
Strongly agree/agree	*1894, 2499*	20.0%	(18.0-22.2)	1.93	(1.61-2.32)		*2624, 2477*	42.6%	(40.4-44.8)	1.85	(1.63-2.10)		
**Men have a naturally higher sex drive than women**						<0.0001						<0.0001	*<0.0001*
Else	*2788, 3441*	18.0%	(16.4-19.7)	1	-		*3351, 2830*	26.0%	(24.3-27.8)	1	-		
Strongly agree/agree	*2049, 2530*	10.9%	(9.4-12.6)	0.56	(0.46-0.68)		*3317, 2925*	42.0%	(40.0-44.1)	2.04	(1.80-2.31)		
**Too much sex in the media**						0.7069						0.1807	*0.4835*
Else	*1986, 2296*	14.6%	(12.8-16.6)	1	-		*2091, 1618*	31.7%	(29.3-34.2)	1	-		
Strongly agree/agree	*2851, 3675*	15.3%	(13.8-16.9)	1.04	(0.85-1.26)		*4577, 4137*	35.1%	(33.5-36.8)	1.10	(0.96-1.26)		

Denominator is those aged 16-74 years with at least one partner in the past year. Unwt, unweighted; wt, weighted

For peer review only

[a] P-value for interaction to determine whether the magnitude of association between each variable and lack of interest in sex differs between men and women

[b] IMD is a multidimensional measure of area (neighbourhood)-level deprivation based on the participant's postcode. IMD scores for England, Scotland and Wales were adjusted before being combined and assigned to quintiles, using a method by Payne and Abel [49].

[c] Participants aged ≥17 years.

[d] Includes arthritis, heart attack, coronary heart disease, angina, other forms of heart disease, hypertension, stroke, diabetes, broken hip or pelvis, bone or hip replacement ever, backache lasting longer than 3 months, any other muscle or bone disease lasting longer than 3 months, depression, cancer and any thyroid condition treated in the past year.

[e] Participants were asked whether they had often been bothered by feeling down, depressed, or hopeless in the past 2 weeks, and whether they had often been bothered by little interest or pleasure in doing things in the past 2 weeks, using a validated two-question patient health questionnaire (PHQ-2).

[f] Opposite and/or same-sex partners

[g] Other means easy with a husband or wife or regular partner, but difficult with a new partner; easy with a new partner, but difficul with a husband or wife or regular partner; difficult with any partner, it depends, sometimes easy, and sometimes difficult.

[h] Participants were asked to rate how happy they were in their relationship from 1 (very happy) to 7 (very unhappy); responses of 1 or 2 were regarded as denoting participants who were happy with their relationship.

[i] A constructed variable to measure readiness, combining consensuality, autonomy of decision making, timing and use of effective contraception

[j] Sexual response problems (for at least 3 months in past year): lacked enjoyment in sex, felt anxious during sex, felt physical pain as a result of sex, felt no excitement or arousal during sex, difficulty in reaching climax, reached a climax more quickly than you would like, trouble getting or keep an erection (men), uncomfortably dry vagina (women)

For peer review only

Table 3: Associations between lack of interest in having sex for at least 3 months in the past year and other sexual response problems lasting 3 months or more in the past year, by sex

	Men							Women						
	Didn't lack interest in sex		Lacked interest in sex			(95%CI)	p-value	Didn't lack interest in sex		Lacked interest in sex			(95%CI)	p-value
Denominators (unwt, wt)	4126, 5077		713, 897		AOR[a]			4540, 3790		2129, 1965		AOR[a]		
**Lacked enjoyment in having sex**							<0.0001							<0.0001
No	97.7%	(97.1-98.1)	81.5%	(78.2-84.4)	1	-		95.9%	(95.1-96.5)	72.5%	(70.2-74.7)	1	-	
Yes	2.3%	(1.9-2.9)	18.5%	(15.6-21.8)	9.78	(7.11-13.46)		4.1%	(3.5-4.9)	27.5%	(25.3-29.8)	8.95	(7.28-11.01)	
**Felt anxious during sex**							<0.0001							<0.0001
No	96.1%	(95.5-96.7)	85.8%	(82.6-88.5)	1	-		97.3%	(96.7-97.7)	89.9%	(88.4-91.3)	1	-	
Yes	3.9%	(3.3-4.5)	14.2%	(11.5-17.4)	4.16	(3.08-5.62)		2.7%	(2.3-3.3)	10.1%	(8.7-11.6)	4.4	(3.43-5.65)	
**Felt physical pain as a result of sex**							0.0213							<0.0001
No	98.4%	(97.9-98.8)	97.1%	(95.6-98.1)	1	-		95.7%	(95.0-96.3)	86.5%	(84.6-88.1)	1	-	
Yes	1.6%	(1.2-2.1)	2.9%	(1.9-4.4)	1.87	(1.10-3.19)		4.3%	(3.7-5.0)	13.5%	(11.9-15.4)	3.55	(2.83-4.45)	
**Felt no excitement or arousal during sex**							<0.0001							<0.0001
No	98.5%	(98.0-98.9)	87.7%	(85.0-90.0)	1	-		97.5%	(96.9-97.9)	80.9%	(79.0-82.7)	1	-	
Yes	1.5%	(1.1-2.0)	12.3%	(10.0-15.0)	9.21	(6.33-13.40)		2.5%	(2.1-3.1)	19.1%	(17.3-21.0)	9.16	(7.16-11.70)	
**Difficulty in reaching climax**							<0.0001							<0.0001
No	92.7%	(91.7-93.5)	80.5%	(76.6-83.8)	1	-		88.3%	(87.2-89.3)	74.9%	(72.7-76.9)	1	-	
Yes	7.3%	(6.5-8.3)	19.5%	(16.2-23.4)	3.08	(2.37-3.99)		11.7%	(10.7-12.8)	25.1%	(23.1-27.3)	2.6	(2.23-3.03)	
**Reached climax more quickly than you would like**							0.0198							0.3658
No	85.6%	(84.3-86.9)	82.0%	(78.7-85.0)	1	-		97.8%	(97.2-98.2)	97.5%	(96.7-98.1)	1	-	
Yes	14.4%	(13.1-15.7)	18.0%	(15.0-21.3)	1.32	(1.05-1.68)		2.2%	(1.8-2.8)	2.5%	(1.9-3.3)	1.18	(0.82-1.69)	
**Trouble getting or keeping an erection**							<0.0001							
no	88.5%	(87.3-89.6)	79.4%	(75.9-82.6)	1	-								
yes	11.5%	(10.4-12.7)	20.6%	(17.4-24.1)	1.97	(1.55-2.51)								
**Uncomfortably dry vagina**														<0.0001
no								90.7%	(89.5-91.7)	80.1%	(77.9-82.1)	1	-	
yes								9.3%	(8.3-10.5)	19.9%	(17.9-22.1)	2.28	(1.89-2.76)	

Denominator is those aged 16-74 years with at least one partner in the past year. Unwt, unweighted; wt, weighted

[a] AOR comparing those lacking interest to those not

For peer review only

Table 2: Factors associated with lacking interest in having sex for at least 3 months in the past year and being distressed about it in sexually active men and women

	Men						Women						
	Denom. (unwt, wt)	%	(95%CI)	Age-adjusted OR	(95%CI)	p-value	*Denom. (unwt, wt)*	%	(95%CI)	Age-adjusted OR	(95%CI)	p-value	*p-value for interaction with sex[a]*
**All**	*4839, 5973*	**8.2%**	**(7.4-9.1)**				*6669, 5755*	**20.8%**	**(19.6-22.0)**				
**Socio-demographics**													
**Age group**						0.0011						<0.0001	*0.8971*
16-24	*1279, 936*	4.8%	(3.7-6.4)	1	-		*1662, 923*	15.2%	(13.4-17.3)	1	-		
25-34	*1376, 1238*	8.0%	(6.7-9.5)	1.7	(1.19-2.41)		*2236, 1246*	20.9%	(19.0-22.8)	1.47	(1.22-1.76)		
35-44	*719, 1298*	9.6%	(7.5-12.3)	2.09	(1.40-3.13)		*1050, 1290*	22.9%	(20.3-25.7)	1.65	(1.34-2.04)		
45-54	*630, 1186*	9.7%	(7.4-12.6)	2.11	(1.38-3.22)		*871, 1186*	23.3%	(20.4-26.6)	1.69	(1.35-2.13)		
55-64	*512, 849*	9.4%	(7.0-12.6)	2.04	(1.30-3.21)		*569, 755*	21.8%	(18.3-25.8)	1.55	(1.20-2.01)		
65-74	*323, 467*	5.5%	(3.4-8.6)	1.13	(0.65-1.99)		*281, 355*	16.5%	(12.4-21.7)	1.10	(0.76-1.59)		
**Index of multiple deprivation (quintiles)[b]**						0.8339						0.0938	*0.4592*
1 (least deprived)	*977, 1279*	8.1%	(6.2-10.4)	1	-		*1248, 1208*	23.3%	(20.7-26.1)	1	-		
2	*962, 1264*	7.4%	(5.7-9.6)	0.92	(0.62-1.36)		*1290, 1208*	20.8%	(18.2-23.5)	0.87	(0.69-1.09)		
3	*942, 1169*	8.3%	(6.4-10.6)	1.05	(0.71-1.55)		*1299, 1116*	19.6%	(17.1-22.4)	0.82	(0.65-1.03)		
4	*967, 1184*	8.8%	(6.9-11.1)	1.14	(0.78-1.66)		*1384, 1137*	21.9%	(19.3-24.7)	0.95	(0.76-1.18)		
5 (most deprived)	*991, 1077*	8.6%	(6.7-10.9)	1.12	(0.75-1.65)		*1448, 1086*	18.2%	(15.9-20.6)	0.75	(0.60-0.94)		
**Education level[c]**						0.4958						0.7324	0.4496
Left school aged 17+	*2862, 3464*	7.9%	(6.8-9.2)	1	-		*4150, 3406*	20.8%	(19.4-22.4)	1	-		
Left school at 16	*1873, 2437*	8.8%	(7.5-10.4)	1.09	(0.85-1.41)		*2409, 2287*	21.1%	(19.3-23.1)	0.97	(0.83-1.14)		
**Employment status**						0.0001						0.0003	*0.1244*
Employed	*3211, 4254*	8.3%	(7.3-9.5)	1	-		*3871, 3517*	21.6%	(20.1-23.2)	1	-		
Full-time education	*542, 431*	4.9%	(2.8-8.6)	0.74	(0.38-1.44)		*693, 423*	14.8%	(11.8-18.4)	0.75	(0.56-1.01)		
Unemployed	*707, 723*	12.1%	(9.5-15.3)	1.56	(1.14-2.13)		*1681, 1282*	22.3%	(19.9-24.9)	1.07	(0.90-1.27)		
Retired	*375, 562*	4.9%	(3.1-7.6)	0.41	(0.23-0.71)		*415, 524*	16.8%	(13.4-20.8)	0.57	(0.41-0.79)		
**Practises religion at least once a month**						0.1638						0.0167	*0.8143*
No	*4283, 5179*	8.5%	(7.5-9.5)	1	-		*5659, 4754*	21.5%	(20.2-22.9)	1	-		

For peer review only

Yes	521, 748	6.4%	(4.4-9.4)	0.73	(0.48-1.13)		956, 945	18.0%	(15.3-20.9)	0.78	(0.63-0.96)		

Table 2 cont.

	Men						Women						
	Denom. (unwt, wt)	%	(95%CI)	Age-adjusted OR	(95%CI)	p-value	*Denom. (unwt, wt)*	%	(95%CI)	Age-adjusted OR	(95%CI)	p-value	*p-value for interaction with sex[a]*
**Health**													
**Self-reported general health**						<0.0001						<0.0001	*0.0969*
Very good/Good	*4123, 5055*	7.0%	(6.1-7.9)	1	-		*5683, 4851*	19.2%	(18.0-20.5)	1	-		
Fair	*580, 745*	13.3%	(10.5-16.8)	2.04	(1.50-2.78)		*780, 709*	27.9%	(24.3-31.9)	1.60	(1.30-1.97)		
Bad/very bad	*135, 171*	22.6%	(15.3-32.1)	3.85	(2.31-6.40)		*206, 195*	33.4%	(26.3-41.4)	2.05	(1.45-2.91)		
**Difficulty walking up stairs because of a health problem**						0.0001						0.0085	*0.1553*
No difficulty	*4475, 5460*	7.6%	(6.7-8.6)	1	-		*6062, 5107*	20.1%	(18.9-21.4)	1	-		
Some difficulty	*278, 393*	12.3%	(8.8-17.0)	1.67	(1.11-2.52)		*450, 482*	24.1%	(20.0-28.7)	1.21	(0.93-1.59)		
Much difficulty/unable to do this	*86, 120*	22.2%	(13.5-34.2)	3.36	(1.79-6.32)		*157, 166*	32.3%	(24.3-41.5)	1.81	(1.21-2.70)		
**Longstanding illness or disability**						<0.0001						<0.0001	*0.0345*
No	*3585, 4259*	6.5%	(5.6-7.5)	1	-		*4843, 4026*	18.7%	(17.4-20.0)	1	-		
Yes	*1253, 1713*	12.5%	(10.6-14.8)	2.09	(1.60-2.74)		*1825, 1729*	25.7%	(23.4-28.2)	1.48	(1.27-1.74)		
**Number of comorbid conditions[d]**						<0.0001						<0.0001	*0.5779*
0	*3453, 3994*	6.4%	(5.5-7.5)	1	-		*4357, 3536*	17.3%	(15.9-18.7)	1	-		
1	*939, 1329*	11.0%	(9.0-13.4)	1.88	(1.37-2.57)		*1555, 1416*	24.1%	(21.7-26.7)	1.54	(1.30-1.83)		
>=2	*446, 650*	13.3%	(10.1-17.4)	2.40	(1.61-3.59)		*755, 802*	30.5%	(26.8-34.4)	2.16	(1.74-2.69)		
**Depressive symptoms[e]**						<0.0001						<0.0001	*0.0370*
No	*4383, 5471*	6.8%	(6.0-7.7)	1	-		*5885, 5149*	18.6%	(17.4-19.8)	1	-		
Yes	*449, 495*	23.7%	(19.3-28.9)	4.36	(3.20-5.94)		*780, 602*	39.6%	(35.4-44.0)	2.94	(2.41-3.59)		
**Treated for depression in the past year**						<0.0001						<0.0001	0.0371
No	*4524, 5630*	7.3%	(6.5-8.2)	1	-		*5770, 5040*	18.5%	(17.3-19.8)	1	-		
Yes	*313, 342*	23.0%	(17.9-29.1)	3.81	(2.71-5.36)		*897, 713*	36.4%	(32.9-40.2)	2.54	(2.12-3.03)		

For peer review only

Table 2 cont.

	Men						Women						
	Denom. (unwt, wt)	%	(95%CI)	Age-adjusted OR	(95%CI)	p-value	*Denom. (unwt, wt)*	%	(95%CI)	Age-adjusted OR	(95%CI)	p-value	*p-value for interaction with sex[a]*
**Menopausal status**												0.9656	
Not menopausal							*5485, 4187*	20.2%	(18.9-21.5)	1	-		
Menopausal							*1167, 1548*	22.5%	(20.0-25.2)	1.01	(0.76-1.32)		
**Circumcised**						0.4097							
No	*3909, 4728*	8.3%	(7.4-9.4)	1	-								
Yes	*857, 1166*	7.5%	(5.7-9.9)	0.87	(0.62-1.22)								
**Sexual behaviour**													
**Number of occasions of sex, past 4 weeks**						<0.0001						<0.0001	*0.5496*
0	*1013, 1163*	10.3%	(8.3-12.7)	1	-		*1408, 1245*	23.2%	(20.7-26.0)	1	-		
1-2	*1160, 1566*	10.5%	(8.6-12.8)	1.02	(0.74-1.42)		*1481, 1373*	24.2%	(21.8-26.9)	1.06	(0.87-1.30)		
3-4	*870, 1168*	7.4%	(5.6-9.8)	0.71	(0.48-1.04)		*1240, 1130*	21.3%	(18.7-24.2)	0.91	(0.73-1.13)		
5+	*1617, 1869*	5.0%	(3.9-6.4)	0.46	(0.33-0.66)		*2078, 1655*	14.7%	(12.9-16.7)	0.58	(0.47-0.72)		
**Masturbation, past 4 weeks**						0.0164						0.7265	*0.0309*
No	*1297, 1828*	6.9%	(5.5-8.6)	1	-		*4032, 3612*	21.1%	(19.6-22.6)	1	-		
Yes	*3531, 4132*	8.8%	(7.7-9.9)	1.42	(1.07-1.88)		*2615, 2114*	20.3%	(18.4-22.2)	0.97	(0.84-1.13)		
**No. of sexual partners, past year[f]**						0.2466						0.0016	*0.4744*
1	*3573, 4824*	8.5%	(7.5-9.6)	1	-		*5440, 5012*	21.6%	(20.3-22.9)	1	-		
2	*539, 513*	6.3%	(4.3-9.1)	0.75	(0.49-1.14)		*570, 364*	16.7%	(13.3-20.6)	0.75	(0.57-0.99)		
3+	*718, 627*	6.8%	(5.1-9.0)	0.82	(0.59-1.15)		*642, 366*	14.1%	(11.0-17.7)	0.62	(0.46-0.83)		
**Paid for sex, past year**						0.4865							
No	*4774, 5896*	8.2%	(7.4-9.2)	1	-								
Yes	*64, 75*	5.6%	(1.8-16.4)	0.66	(0.20-2.15)								
**Ever taken drugs to assist sexual performance**						0.0022						0.1055	*0.5305*
No	*4188, 5180*	7.6%	(6.7-8.6)	1	-		*6478, 5624*	20.6%	(19.5-21.8)	1	-		
Yes	*636, 776*	12.1%	(9.5-15.4)	1.63	(1.19-2.23)		*184, 124*	25.9%	(19.2-33.9)	1.38	(0.93-2.05)		

For peer review only

Table 2 cont.

	Men						Women						
	Denom. (unwt, wt)	%	(95%CI)	Age-adjusted OR	(95%CI)	p-value	*Denom. (unwt, wt)*	%	(95%CI)	Age-adjusted OR	(95%CI)	p-value	*p-value for interaction with sex[a]*
**Relationship context**													
**Relationship status**						0.03						<0.0001	*0.0307*
Living with partner	*2708, 4266*	8.8%	(7.7-10.1)	1	-		*3967, 4168*	23.4%	(21.9-24.9)	1	-		
In a steady relationship, not living together	*947, 760*	6.9%	(5.3-9.0)	0.78	(0.56-1.09)		*1360, 790*	15.4%	(13.4-17.7)	0.59	(0.49-0.71)		
Not in a steady relationship, but previously cohabited	*446, 388*	8.8%	(6.2-12.2)	1.00	(0.67-1.48)		*752, 462*	13.6%	(11.1-16.6)	0.51	(0.40-0.66)		
Not in a steady relationship, never cohabited	*727, 551*	4.7%	(3.3-6.8)	0.52	(0.34-0.81)		*580, 330*	11.0%	(8.2-14.5)	0.39	(0.28-0.55)		
**Duration of most recent sexual relationship**						0.0143						<0.0001	*0.0719*
1 year or less	*1462, 1260*	5.5%	(4.3-7.1)	1	-		*1597, 998*	11.2%	(9.4-13.2)	1	-		
Between 1 and 5 years	*1247, 1227*	9.0%	(7.3-11.0)	1.67	(1.18-2.36)		*1758, 1148*	18.5%	(16.5-20.7)	1.81	(1.44-2.29)		
Between 5 and 15 years	*1065, 1484*	9.3%	(7.5-11.6)	1.68	(1.17-2.43)		*1774, 1458*	25.2%	(23.0-27.6)	2.81	(2.23-3.55)		
Over 15 years	*1004, 1904*	8.8%	(7.1-10.8)	1.47	(0.97-2.22)		*1445, 2036*	23.8%	(21.5-26.2)	2.83	(2.13-3.75)		
**Always easy to talk about sex with partners[g]**						0						<0.0001	*0.4854*
Yes	*1695, 1899*	4.8%	(3.8-6.0)	1	-		*1746, 1451*	11.4%	(9.7-13.2)	1	-		
No/other	*3122, 4048*	9.8%	(8.7-11.1)	2.15	(1.62-2.87)		*4907, 4289*	23.9%	(22.5-25.3)	2.43	(2.02-2.93)		
**Happy with relationship[h]**						<0.000 1						<0.0001	*0.9717*
Yes	*1951, 2791*	7.1%	(5.9-8.6)	1	-		*2736, 2601*	18.6%	(16.9-20.4)	1	-		
Other	*995, 1430*	13.3%	(11.2-15.8)	2.01	(1.51-2.66)		*1640, 1617*	31.4%	(28.8-34.0)	2.00	(1.69-2.37)		
**Participant does not share same level of interest in sex as partner**							0.0311						<0.0001
No/other	*2270, 3233*	8.5%	(7.2-10.0)	1	-		*3211, 3064*	15.0%	(13.6-16.4)	1	-		
Yes	*676, 988*	11.6%	(9.2-14.4)	1.41	(1.03-1.92)		*1166, 1155*	46.2%	(42.9-49.6)	4.91	(4.13-5.83)		

Table 2 cont.

	Men						Women						
	Denom. (unwt, wt)	**%**	**(95%CI)**	**Age-adjusted OR**	**(95%CI)**	**p-value**	*Denom. (unwt, wt)*	**%**	**(95%CI)**	**Age-adjusted OR**	**(95%CI)**	**p-value**	***p-value for interaction with sex*** [a]
**Participant does not share same sexual likes & dislikes as partner**						0.0975						<0.0001	*0.0212*
No/other	*2650, 3803*	8.9%	(7.7-10.2)	1	-		*4079, 3908*	22.1%	(20.6-23.6)	1	-		
Yes	*296, 418*	12.2%	(8.6-17.0)	1.43	(0.94-2.18)		*297, 310*	41.9%	(35.6-48.6)	2.55	(1.93-3.37)		
**Partner experienced sexual difficulties in past year**						0.0027						<0.0001	*0.6889*
No/other	*2431, 3454*	8.3%	(7.2-9.6)	1	-		*3726, 3498*	22.1%	(20.6-23.7)	1	-		
Yes	*513, 763*	13.2%	(10.2-17.0)	1.68	(1.20-2.35)		*649, 719*	30.4%	(26.5-34.6)	1.58	(1.27-1.95)		
**Does not feel emotionally close to partner when have sex**						0.0225						<0.0001	*0.8228*
No/other	*2904, 4165*	9.1%	(7.9-10.3)	1	-		*4263, 4108*	22.9%	(21.5-24.4)	1	-		
Yes	*42, 56*	21.0%	(10.2-38.3)	2.69	(1.15-6.29)		*112, 109*	47.0%	(36.4-57.8)	2.98	(1.92-4.63)		
**Lifestyle**													
**1+ child(ren) aged <5 in household**						0.1047						0.0004	*0.0042*
No, none	*4100, 5015*	8.6%	(7.6-9.6)	1	-		*4997, 4671*	20.2%	(18.9-21.5)	1	-		
Yes, 1+	*727, 941*	6.3%	(4.6-8.5)	0.75	(0.52-1.06)		*1664, 1074*	23.5%	(21.2-25.9)	1.34	(1.14-1.58)		
**Pregnant in the last year**												0.5927	
No							*4227, 4122*	21.8%	(20.4-23.4)	1	-		
Yes							*437, 273*	20.7%	(16.6-25.6)	0.92	(0.69-1.24)		
**Used hormonal contraceptive, past year**												0.1141	
No							*3759, 3838*	20.7%	(19.2-22.3)	1	-		
Yes							*2806, 1831*	20.9%	(19.1-22.7)	1.14	(0.97-1.35)		
**Sexual health indicators**													
**Ever diagnosed with a sexually transmitted infection**						<0.0001						0.0002	*0.0291*
No (or only thrush)	*4148, 5128*	7.3%	(6.5-8.3)	1	-		*5455, 4861*	20.0%	(18.7-21.3)	1	-		
Yes (excluding thrush)	*677, 830*	13.7%	(11.0-17.0)	2.02	(1.51-2.70)		*1206, 888*	25.1%	(22.3-28.1)	1.39	(1.16-1.65)		

Table 2 cont.

For peer review only

	Men						Women						
	Denom. (unwt, wt)	%	(95%CI)	Age-adjusted OR	(95%CI)	p-value	*Denom. (unwt, wt)*	%	(95%CI)	Age-adjusted OR	(95%CI)	p-value	*p-value for interaction with sex[a]*
**Ever experienced non-volitional sex**						<0.0001						<0.0001	*0.1143*
No	*4706, 5825*	7.9%	(7.1-8.9)	1	-		*5815, 5055*	19.4%	(18.2-20.7)	1	-		
Yes/Don't know	*133, 148*	19.4%	(13.1-27.7)	2.83	(1.74-4.59)		*848, 695*	30.9%	(27.3-34.6)	1.86	(1.55-2.25)		
**Sexual competence at first sex[i]**						0.4876						<0.0001	*0.0787*
Not competent	*2408, 3039*	8.7%	(7.5-10.0)	1	-		*3438, 2927*	23.6%	(21.9-25.3)	1	-		
Competent	*2302, 2784*	7.8%	(6.6-9.2)	0.91	(0.71-1.18)		*3097, 2716*	17.7%	(16.1-19.3)	0.70	(0.61-0.81)		
**Number of other sexual response problems experienced[j]**						<0.0001						<0.0001	*0.0262*
0	*3209, 3947*	5.3%	(4.4-6.3)	1	-		*4377, 3759*	12.9%	(11.7-14.1)	1	-		
1	*1061, 1350*	6.1%	(4.7-7.8)	1.14	(0.81-1.59)		*1217, 1087*	21.7%	(19.0-24.6)	1.86	(1.53-2.26)		
2+	*570, 678*	29.7%	(25.4-34.4)	7.57	(5.68-10.10)		*1075, 909*	52.4%	(48.9-56.0)	7.48	(6.25-8.94)		
**Attitudes**													
**People are under pressure to have sex**						0.1437						<0.0001	*0.2192*
Else	*1799, 2264*	7.4%	(6.0-9.0)	1	-		*1851, 1570*	16.4%	(14.5-18.5)	1	-		
Strongly agree/agree	*3038, 3707*	8.7%	(7.6-9.9)	1.21	(0.94-1.57)		*4817, 4185*	22.4%	(21.0-23.9)	1.47	(1.24-1.74)		
**People want less sex as they age**						0.0005						<0.0001	*0.8045*
Else	*2943, 3472*	6.7%	(5.7-7.8)	1	-		*4044, 3278*	17.1%	(15.8-18.6)	1	-		
Strongly agree/agree	*1894, 2499*	10.3%	(8.8-12.1)	1.58	(1.22-2.04)		*2624, 2477*	25.6%	(23.7-27.6)	1.64	(1.43-1.90)		
**Men have a naturally higher sex drive than women**							<0.0001						<0.0001
Else	*2788, 3441*	10.2%	(8.9-11.5)	1	-		*3351, 2830*	15.9%	(14.4-17.4)	1	-		
Strongly agree/agree	*2049, 2530*	5.5%	(4.4-6.9)	0.52	(0.39-0.68)		*3317, 2925*	25.5%	(23.8-27.4)	1.81	(1.56-2.09)		
**Too much sex in the media**						0.3477						0.0693	*0.8856*
Else	*1986, 2296*	7.5%	(6.3-9.0)	1	-		*2091, 1618*	18.8%	(16.8-20.9)	1	-		
Strongly agree/agree	*2851, 3675*	8.6%	(7.5-9.9)	1.13	(0.88-1.46)		*4577, 4137*	21.6%	(20.2-23.0)	1.16	(0.99-1.36)		

Denominator is those aged 16-74 years with at least one partner in the past year. Unwt, unweighted; wt, weighted

[a] P-value for interaction to determine whether the magnitude of association between each variable and lack of interest in sex differs between men and women

For peer review only

[b] IMD is a multidimensional measure of area (neighbourhood)-level deprivation based on the participant's postcode. IMD scores for England, Scotland and Wales were adjusted before being combined and assigned to quintiles, using a method by Payne and Abel [49].

[c] Participants aged ≥17 years.

[d] Includes arthritis, heart attack, coronary heart disease, angina, other forms of heart disease, hypertension, stroke, diabetes, broken hip or pelvis, bone or hip replacement ever, backache lasting longer than 3 months, any other muscle or bone disease lasting longer than 3 months, depression, cancer and any thyroid condition treated in the past year.

[e] Participants were asked whether they had often been bothered by feeling down, depressed, or hopeless in the past 2 weeks, and whether they had often been bothered by little interest or pleasure in doing things in the past 2 weeks, using a validated two-question patient health questionnaire (PHQ-2).

[f] Opposite and/or same-sex partners

[g] Other means easy with a husband or wife or regular partner, but difficult with a new partner; easy with a new partner, but difficul with a husband or wife or regular partner; difficult with any partner, it depends, sometimes easy, and sometimes difficult.

[h] Participants were asked to rate how happy they were in their relationship from 1 (very happy) to 7 (very unhappy); responses of 1 or 2 were regarded as denoting participants who were happy with their relationship.

[i] A constructed variable to measure readiness, combining consensuality, autonomy of decision making, timing and use of effective contraception

[j] Sexual response problems (for at least 3 months in past year): lacked enjoyment in sex, felt anxious during sex, felt physical pain as a result of sex, felt no excitement or arousal during sex, difficulty in reaching climax, reached a climax more quickly than you would like, trouble getting or keep an erection (men), uncomfortably dry vagina (women)

For peer review only

BMJ Open

What factors are associated with reporting lacking interest in sex and how do these vary by gender?: Findings from the third British National Survey of Sexual Attitudes and Lifestyles

Journal:	*BMJ Open*
Manuscript ID	bmjopen-2017-016942.R1
Article Type:	Research
Date Submitted by the Author:	16-Jun-2017
Complete List of Authors:	Graham, Cynthia; University of Southampton, Psychology<br>Mercer, Catherine; University College London, Research Department of Infection and Population Health<br>Tanton, Clare; University College London, Research Department of Infection & Population Health<br>Jones, Kyle; University College London, Research Department of Infection and Population Health<br>Johnson, Anne; University College London, Research Department of Infection and Population Health<br>Wellings, Kaye; London School of Hygiene and Tropical Medicine,<br>Mitchell, Kirstin; London School of Hygiene and Tropical Medicine
**Primary Subject Heading**:	Sexual health
Secondary Subject Heading:	Epidemiology
Keywords:	SEXUAL MEDICINE, EPIDEMIOLOGY, Sexual and gender disorders < PSYCHIATRY

SCHOLARONE™
Manuscripts

What factors are associated with reporting lacking interest in sex and how do these vary by gender?: Findings from the third British National Survey of Sexual Attitudes and Lifestyles

Graham, C. A.,[1] Mercer, C. H.,[2] Tanton, C. [2] Jones, K. G.,[2] Johnson, A. M.,[2] Wellings, K.,[3] Mitchell, K. R.[4]

[1]Centre for Sexual Health Research, Department of Psychology, University of Southampton, Southampton, U.K.
[2]Centre for Sexual Health and HIV Research, Research Department of Infection & Population Health, University College London, London, U.K.
[3]Centre for Sexual and Reproductive Health Research, Department of Social and Environmental Health Research, London School of Hygiene and Tropical Medicine, London, U.K.
[4]MRC/CSO Social and Public Health Sciences Unit, University of Glasgow, 200 Renfield Street, Glasgow G2 3QB, U.K.

Corresponding author:
Cynthia A. Graham
Department of Psychology
Faculty of Social, Human, and Mathematical Sciences
Shackleton Building (B44)
University of Southampton
Highfield, Southampton
SO17 1BJ UK
tel: 023 8059 3091
e-mail: C.A.Graham@soton.ac.uk

Word count: 3,900

For peer review only

ABSTRACT

Objectives: To investigate factors associated with reporting lacking interest in sex, and how these vary by gender.

Setting: British general population.

Design: Complex survey analyses of data collected for a cross-sectional probability sample survey, undertaken 2010-12, specifically logistic regression to calculate age-adjusted odds ratios (AOR) to identify associated factors.

Participants: 4,839 men and 6,669 women aged 16-74 years who reported >=1 sexual partner (opposite-sex or same-sex) in the past year for the third National Survey of Sexual Attitudes and Lifestyles [Natsal-3].

Main outcome measure: Lacking interest in sex for >=3 months in the past year.

Results: Overall, 15.0% (13.9-16.2) of men and 34.2% (32.8-35.5) of women reported lacking interest in sex. This was associated with age and physical and mental health for both men and women, including self-reported general health and current depression. Lacking interest in sex was more prevalent among men and women reporting STI diagnoses (ever), non-volitional sex (ever), and holding sexual attitudes related to normative expectations about sex. Some gender similarities in associated relationship and family-related factors were evident, including partner having had sexual difficulties in the last year (M: AOR:1.41 [1.07-1.86]; W: AOR:1.60 [1.32-1.94]), not feeling emotionally close to partner during sex (M: 3.74 [1.76-7.93]; W: 4.80 [2.99-7.69], and ease of talking about sex (M: 1.53 [1.23-1.90] W: 2.06 [1.77-2.39]). Among women only, lack of interest in sex was higher among those in a relationship of >1 year in duration and those not sharing the same level of interest (4.57 [3.87-5.38]) or preferences (2.91 [2.22-3.83]) with a partner.

Conclusions: Both gender similarities and differences were found in factors associated with lacking interest in sex, with the most marked differences in relation to some relationship variables. Findings highlight the need to assess, and if appropriate, treat lacking interest in sex in a holistic and relationship-specific way.

For peer review only

ARTICLE SUMMARY

Strengths and limitations of this study

- This study used nationally representative data to investigate factors associated with reporting lacking interest in sex, and how these vary by gender, in the British population.
- Few previous population-based studies have obtained data on low sexual interest from men and women and made direct comparisons between them.
- The study included detailed assessment of a range of relationship context and attitudinal variables seldom included in previous population-based surveys.
- Information about lacking interest in sex was assessed with a single item, asking participants whether they had lacked interest in having sex for a period of three months or more in the past year. Those who reported this were also asked whether they experienced associated distress.
- The cross-sectional data do not allow us to establish the causal direction of associations between lacking interest in sex and variables of interest.

For peer review only

In Britain's third National Survey of Sexual Attitudes and Lifestyles (Natsal-3) lacking interest in sex was the most common sexual difficulty reported by both men and women (1). Lacking interest in sex for 3 months or more in the past year was twice as common in women compared to men. When duration and symptom severity criteria are considered (i.e., that symptoms last six months or more and occur "very often" or "always") these prevalence estimates are much lower (2), but the gender difference is maintained.

Researchers have paid more attention to problems of low sexual interest in women than in men (3-5). Among men the predominant focus has been on erectile functioning and on physiological causes of lacking interest in sex such as hormonal status, rather than on psychosocial determinants. This lack of attention to male problems is reflected in recent revisions to the Diagnostic and Statistical Manual (DSM-5) classification of sexual disorders (6) which involved major changes to sexual arousal and desire disorder categories in women, but no substantive changes for male disorders.

Most but not all studies involving men have reported an association between low sexual interest and increasing age (for review, see 7). However, there are conflicting findings on the association with physical and mental health (8,9). Limited research suggests that psychosocial and relationship factors may also be associated with low sexual desire in men (8, 10-12).

Among women, factors that have been consistently associated with lacking interest in sex are relationship problems, relationship quality, and partner's sexual functioning (13-17), poor physical health (18), and negative mood states/depression (13, 18,19). There are inconsistent findings on the association between low sexual

For peer review only

interest and both age and menopausal status (14,18). Few large-scale surveys have examined possible links between lacking interest in sex and either sexual attitudes or sexual behaviour. In the second wave of the British National Survey of Sexual Attitudes and Lifestyles (Natsal-2), among women, lacking interest in sex was associated with lower frequency of sex and attitudes according sex low priority (20).

Studies have, for the most part, used small, clinical samples of patients seeking treatment for low sexual desire problems. The potential for bias in such studies is revealed in previously reported findings from Natsal-3 that only around a third of men and women with one or more sexual function problems meeting DSM 5 morbidity criteria had sought professional help in the last year. (2). The few large-scale probability-based surveys involving both men and women have focused on associations between low sexual desire and sociodemographic factors.

In summary, the evidence on the factors associated with men's and women's reports of low sexual desire is drawn largely from non-representative samples, is somewhat equivocal and, in men, sparse. Given that most previous research has involved non-representative samples, it is important to explore how correlates might differ in a population-based sample. Understanding the correlates of lacking interest in sex is key to informing therapeutic options for this group.

The research questions addressed in this paper are: (1) What sociodemographic, relationship, sexual behaviour, and sexual attitudinal factors are associated with lacking interest in sex in sexually active men and women?; (2) To what extent do these factors vary by gender?; (3) To what extent does lacking interest in sex co-exist with other sexual function problems?

METHOD

For peer review only

Participants and Procedure

Natsal-3 is a probability sample survey of 15,162 men and women aged 16-74 years in Britain, interviewed between September 2010 and August 2012. A multi-stage, clustered, and stratified probability sample design was used and participants were interviewed in their homes by professional interviewers using a combination of computer-assisted personal interviews (CAPI), and computer-assisted self-interviews (CASI) for the more sensitive questions (including, of relevance to this paper, those on sexual function), Interviewers were present in the room while participants completed the CASI, but did not view responses (20). After weighting to adjust for unequal probabilities of selection and to match the British population in terms of age, gender and geographical region, the Natsal-3 sample was broadly representative, on key variables, of the British population as described by the 2011 Census (21).

The estimated response rate was 57.7%, and the estimated cooperation rate (the number of interviews completed from eligible addresses for which contact was made) was 65.8% (of all eligible addressed contacted) (22). More extensive details of the survey methodology and sample characteristics are published elsewhere (21,22) and for demographic characteristics of the sample, see (22). Participants provided oral informed consent for interviews and the survey was approved by the NRES Committee South-Central – Oxford A (Ref.: 10/H0604/27).

For the current analyses, only respondents who reported >=1 sexual partner (opposite-sex or same-sex) in the past year were included (4,839 men and 6,669 women).

Outcome Measures

For peer review only

Items were drawn from the Natsal-SF, a measure of sexual function, designed and validated for population surveys (Comparative Fit Index = 0.963 Tucker Lewis Index = 0.951; Root Mean Square Error of Approximation = 0.064). The measure comprised items on problems with sexual response, relational aspects of sexual function, and self-appraisal of sex life (23,24). Participants who reported at least one sexual partner in the past year (hereon 'sexually active participants') were asked: *'In the last year, have you experienced any of the following for a period of 3 months or longer?'* and were given a list of difficulties and asked to indicate which they had experienced. The list included *'Lacked interest in having sex'*. Those indicating this difficulty were defined as lacking interest in having sex for a period of three months or more in the past year (the outcome for this analysis). Individuals reporting lacking interest in sex for at least 3 months were then asked *'And how do you feel about this?'* with response options: *not at all distressed, a little distressed, fairly distressed; very distressed*. Those answering a little, fairly or very distressed were defined as lacking interest in sex and having distress about this symptom (outcome for sensitivity analysis, see below).

Statistical Analysis

All analyses were done using the complex survey functions of STATA (version 14; StataCorp LP, College Station, Texas) to account for the weighting, clustering, and stratification of the data. We used multivariable logistic regression to calculate age-adjusted odds ratios (AOR) to examine the associations between reports of lacking interest in sex lasting three months or longer in the past year, and sociodemographic, health, relationship, sexual behaviour, and sexual attitude variables. For each variable, we also tested the interaction with gender, to see if the magnitude of the associations

For peer review only

between the above factors and reports of lacking interest in sex was the same for men and women. We conducted a sensitivity analysis for the outcome variable reporting lack of interest in sex lasting three months or longer *and* distress about this symptom to assess whether similar associations were found. We also examined the association between reporting lacking interest in sex and the other sexual function problems asked about in Natsal-3, using AORs.

RESULTS

Overall, 15.0 (95% CI 13.9-16.2) of sexually active men and 34.2% (95% CI 32.8-35.5) of sexually active women reported lacking interest in sex for three months or longer in the year prior to interview. Table 1 presents the associations between lacking interest in sex and sociodemographic, health, relationship, sexual behaviour, and sexual attitudinal variables for men and women.

Age was significantly associated with lacking interest in sex. Prevalence increased with age, being lowest among younger participants (16-24 years; M: 11.5%; W: 24.8%) and peaking in men aged 35-44 years (17.2%) and in women aged 55-64 years (38.8%). Regarding demographic variables, after adjusting for age, lack of interest was associated with leaving school at 16 (men only; AORS: 1.31), being unemployed (men only AORs: M: 1.44), and less frequent religious practice (women only; AOR: 0.79). In women, after adjusting for age, those who were students or retired were less likely to lack desire.

After adjusting for age, there were associations between all physical and mental health variables assessed and lacking interest in sex. Individuals in poorer health (AORs: M: 3.29; W: 1.93), those who had much difficulty walking upstairs (AORs: M: 2.68; W: 1.55), those with a longstanding medical condition (AORs: M 1.76;

For peer review only

W: 1.35), and those who had screened positive for current depression (AORs: M: 2.95; W: 2.79) or who had been treated for depression in the past year (AORs: M: 2.82; W: 2.32) were more likely to report lacking interest in sex. The magnitude of these associations was similar for men and women. A greater number of comorbid health conditions was significantly associated with lacking interest in sex among both men and women. Menopausal status in women and circumcision in men were not associated with the likelihood of lacking sexual interest.

Regarding sexual behaviour, among both men and women, lack of interest was associated with frequency of sexual activity (defined as vaginal, oral or anal intercourse) in the four weeks prior to interview; 12.4% of men, and 33.8% of women who reported having engaged in 3-4 sexual acts reported lack of interest, vs. 20.7% of men and 42.9% of women who reported no sexual activity. Associations with recent masturbation differed by gender; lack of interest in sex was slightly *more* common among men who reported having recently masturbated but *less* common among women who did so. Women with three or more partners in the past year were less likely to report low sexual interest than those with only one partner (AOR: 0.70) but there was no association between partner numbers and lacking interest in sex in men. Among men only, those who reported ever having taken drugs to assist sexual performance were more likely to report lacking interest in sex (AOR: 1.36). A similar magnitude association was seen for women (AOR: 1.39) however, fewer women reported ever having taken drugs and the 95%CI therefore crosses 1.

Associations were found between lacking interest in sex and several relationship contextual variables and for many of these variables associations were stronger for women than for men. For both men and women, lack of interest was

For peer review only

associated with relationship status; women living with a partner were more likely to lack interest in sex than those in other relationship categories (see Table 1). For women, all relationship categories had lower AORs than living with partner. Duration of most recent sexual relationship was significantly associated with lacking interest in sex only among women, being more common among those in longer relationships.

Among both men and women, there was an association between ease of communication and lacking interest in sex. Those who found it “always easy to talk about sex” with their partner were less likely to report low interest. Lack of interest was more likely among those whose partner had sexual difficulties in the last year, and those who reported a lower assessment of happiness with the relationship, and not feeling emotionally close to partner during sex. Among women but not men, not sharing the same level of sexual interest with a partner, and not sharing the same sexual likes and dislikes, were also associated.

Having been pregnant in the last year was associated with lacking sexual interest as was having one or more young child(ren) (women only). Lack of interest in sex was significantly associated with sexual health indicators, including previous STI diagnosis and ever having experienced non-volitional sex. The strength and direction of associations was similar for men and women, except for reporting another sexual function problem, which was significant for two or more problems in men, but one or more problems in women. Sexual competence at first sex was significantly associated with lack of interest in sex only among women.

Regarding attitudinal variables, both men and women who endorsed statements that “people are under pressure to have sex” and “people want less sex as they age” were more likely to report lacking interest in sex over the past year. The

For peer review only

only attitudinal variable that showed a significant interaction with gender was that which related to men having a “naturally higher sex drive than women.” Men who agreed with this statement were *less* likely than those who disagreed to lack interest in sex, while the reverse was true among women.

Table 2 presents the associations between lacking interest in sex and being distressed about this (as a measure/marker of severity), and the above sociodemographic, health, and sexual relationship/behaviour variables. While prevalence was lower, the associations and the interactions with gender were generally similar; however, some of the previous gender-specific associations with variables (e.g., masturbation, and pregnancy in women, and education in men) were no longer significant when the outcome variable was reported low sexual interest *and* associated distress. In addition, some associations became stronger when considering only those who reported a distressing lack of interest in sex (vs. lack of interest without any reported distress). For example, the association between depressive symptoms and having been treated for depression in the past year, was stronger in men than in women.

Regarding the association between reporting lacking interest in sex and the other sexual function problems asked about in Natsal-3, the strongest (positive) associations were for lacking enjoyment in sex (AORs=9.78 and 8.95 for men and women, respectively), followed by feeling no excitement or arousal during sex (AORs=9.21 and 9.16 for men and women, respectively) (see Table 3).

DISCUSSION

We identified a broad range of factors, including some that have not been explored in previous large-scale surveys, that were associated with men’s and

For peer review only

women's reports of lacking interest in sex in a representative British population-based survey. Our findings, discussed below, revealed some gender similarities as well as some interesting gender differences. The strongest evidence for gender differences was for the relationship context variables, where associations with lacking interest in sex were much stronger for women than for men.

Interpretation of findings in context of previous research

Our finding relating to differences by age is consistent with some, but not all, results from previous research which has yielded generally inconsistent findings. Some studies have, like ours, shown a higher prevalence of sexual interest problems in older than in younger women (25-27). Others have found no association between age and low sexual interest complaints (14, 28) and yet more have shown lack of sexual interest to be more common among younger women (18). Whereas we found a marginal relationship with age in men, some studies (though not all e.g., 29) have found a stronger relationship (12, 30). It is possible that the varied findings might in part be a result of varied definitions of low sexual interest or differences in sampling.

The finding in this analysis that having young children appears to increase the likelihood of reporting lack of sexual interest for women, but not for men, remains unchanged since the previous Natsal-2 survey (31). This may be due to fatigue associated with a primary caring role (32), the fact that daily stress appears to affect sexual functioning in women more than men (33), or possibly a shift in focus of attention attendant on bringing up small children.

The finding of a link between lacking interest in sex and lacking enjoyment in sex and/or feeling no excitement or arousal during sex is not surprising and has been shown in previous studies (3). The strong associations between lack of interest in sex

For peer review only

and physical and mental health indicators, which we observed for both men and women, is not entirely consistent with findings from other studies. While this link has been persuasively shown for women (13, 18, 19), in men, the evidence is more equivocal. In a study of men attending an outpatient clinic for sexual problems, psychological symptoms such as anxiety and depression were more predictive of low sexual desire than hormonal or other physical markers (11). In contrast, DeRogatis et al. (9), in their study of men with erectile dysfunction, observed no differences in depressive symptoms, concurrent illness, or medication use between men with and without symptoms of low sexual desire.

The gender differences in associations between masturbation and a lack of sexual interest are interesting and have been explored in few previous population-based studies. Our observation that lack of interest was *more* commonly reported by men who had recently masturbated, but *less* commonly reported by women who had done so may reflect a tendency among women for self-pleasuring to be, not a substitute for partnered sex but instead a part of a broader repertoire of sexual fulfilment; this possibility is worthy of further exploration. In contrast, for men frequency of masturbation reflects reduced frequency of partnered sex (34). However, it is worth noting that in the U.S. National Health and Social Life Survey, lifetime number of sexual partners and masturbation practices were unrelated to the likelihood of sexual desire difficulties for either men or women (35).

Our observation that duration of most recent sexual relationship showed a strong association with lacking interest in sex in women is consistent with previous studies (15, 17). There has been little comparable research on men with which to corroborate the absence of such an association among men in our analysis.

For peer review only

Our data confirm the importance of the relational context in individuals' level of sexual interest. The strong associations between relationship and partner factors and sexual interest are consistent with those shown in many previous studies relating to women (13-17) and with a much smaller literature in men (36,37). In particular, sexual dysfunction in a male partner has previously been associated with women's levels of sexual desire (15, 38, 39), and sexual desire discrepancy in couples has been linked to lower reported relationship satisfaction and more couple conflict (40).

The strong links found between several key sexual health outcomes and lack of interest in sex are interesting; among both men and women, reporting an STI diagnosis and non-volitional sex were associated with reporting lack of interest in sex. Our finding that lacking "sexual competence" at first sexual intercourse was linked with subsequent lack of interest in sex among women but not men, may reflect a greater salience of contextual aspects of first sex for women. More women than men report being pressured by a partner on the first occasion of heterosexual intercourse, and to have subsequently experienced regret about first sexual experiences (41). These findings suggest that for women, early sexual experiences may shape future sexual encounters/relationships to a greater extent than for men.

To our knowledge no previous studies have assessed the association between attitudes toward sexual matters and lack of interest in sex. Endorsing the assumption that "people want less sex as they age" was associated with lack of interest in both genders. It might be that this belief contributes to a decline in interest, or – equally plausible – that those who lack interest adopt this attitude to avoid viewing their experience as problematic. Interestingly, men who endorsed the view that "men have a higher sex drive than women" were significantly *less* likely to report lacking interest

For peer review only

in sex, whereas women who agreed with this statement were *more* likely to do so. If people responded to this statement with reference to their own relationship, these findings may be seen as making intuitive sense. The results suggest that endorsing stereotypical gender-norms related to sex may adversely affect women more than men.

Strengths and Limitations

Strengths of our study include the use of national probability sample survey data involving both men and women across a wide age range (21, 22). With a few exceptions (e.g., 12, 14, 29, 42), most surveys on sexual desire problems have sampled either men *or* women, precluding direct comparisons within the same sample. Another strength was the detailed and holistic examination of relationship context and attitudinal variables, which few previous studies have reported. Response rates for Natsal-3 were also similar to those of other major social surveys in Britain (43) and higher than many previous surveys of sexual problems (35, 44).

Limitations include the cross-sectional nature of the data, which mean that we are unable to infer temporality and causality. We included only respondents who reported >=1 sexual partner (opposite-sex or same-sex) in the past year, excluding those who had not had sex because of lack of interest. We only used a single item to assess lacking interest in sex, although we additionally took account of whether those who reported this also reported that it caused them distress, as a way of trying to capture more problematic lack of interest. This sensitivity analysis enabled us to demonstrate that for most variables, similar associations exist regardless of whether or not distress was reported. It is important to acknowledge, however, that these data do not necessarily correspond to clinical diagnoses. Finally, we have tested many

For peer review only

associations within this study and some will have been significant by chance. We did not formally correct our p-values and therefore where $0.01<p<0.05$ we advise caution in concluding association.

Implications for Research and Practice

The findings indicate that lack of interest in sex is associated with a broad range of factors across sociodemographic, relationship, sexual behaviour, and sexual attitudinal domains. There are both research and clinical applications of our results.

Firstly, our findings underscore the importance of the relational context in understanding low sexual interest in both men and women. For women in particular, the experience of sexual interest appears strongly linked with their perceptions of the quality of their relationships, their communication with partners, and their expectations/attitudes about sex. Our findings support the view that transient (and often adaptive) reductions in sexual desire are not evidence of "dysfunction" (45). In the context of the recent FDA approval of flibanserin, the first drug to treat low sexual desire in women (46), these findings are relevant to the current debate about whether striving for a pharmaceutical solution to women's sexual desire problems is an appropriate and feasible goal (45, 47). Some authors have suggested that women with complaints of low sexual interest might benefit most from integrative approaches that accord with a biopsychosocial model (48).

Secondly, our findings on the strong association between open sexual communication (i.e., "finding it always easy to talk about sex") and a reduced likelihood of reporting lack of interest in sex, particularly for women, emphasise the importance of providing a broad sexual and relationships education, rather than limiting attention only to adverse consequences of sex and how to prevent them.

For peer review only

Similarly, the important role of early sexual experiences, and sexual “competence,” especially for women, in shaping later experiences of sexual desire supports the need for comprehensive sex education.

In a clinical context, our findings emphasise the importance of health care professionals assessing psychological and interpersonal variables in individuals presenting with complaints of low sexual interest (49). In couple therapy, it is important that therapists have an awareness of the differences between men and women in the factors associated with low sexual interest. For example, among the subgroup of participants reporting both lack of interest in sex and related distress, we found a stronger association between depressive symptoms and treatment for depression in the last year among men compared with women. Lastly, our findings support previous research on the critical role of physical and mental health in understanding low sexual interest problems experienced by men and women (11, 18).

Conclusions

This study extends our understanding of the factors associated with lack of interest in sex in men and women, the gender similarities and differences, and highlights the need to assess and – if necessary – treat sexual desire problems in a holistic and relationship- as well as gender-specific way.

For peer review only

Contribution to authorship

The paper was conceived by CAG, CHM, AMJ, KW, and KRM. CAG wrote the first draft, with further contributions from all authors. Statistical analyses were undertaken by CHM, CT, and KGJ. CHM, AMJ (Principal Investigator) and KW, initial applicants on Natsal-3, wrote the study protocol and obtained funding. Natsal-3 questionnaire design, ethics applications, and piloting were undertaken by CHM, CT, AMJ, KW, and KRM. Data management was undertaken by NatCen Social Research, UCL and LSHTM. All authors contributed to data interpretation, reviewed successive drafts and approved the final version of the manuscript.

Competing interests statement

AMJ has been a Governor of the Wellcome Trust since 2011. The remaining authors have nothing to disclose. Completed disclosure of interests form available to view online as supporting information.

Funding Statement

Natsal-3 was supported by grants from the U.K. Medical Research Council (G0701757) and the Wellcome Trust (084840), with support from the Economic and Social Research Council and the Department of Health. Since September 2015, Kirstin Mitchell has been supported by the United Kingdom Medical Research Council grant MC_UU_12017/11, and Scottish Government Chief Scientist Office grant SPHSU11.

Details of ethics approval

Natsal-3 was approved by the NRES Committee South Central-Oxford A (Ref: 10/H0604/27) on 12 July 2010. Participants provided oral informed consent for interviews.

Data sharing statement

The Natsal-3 dataset is publicly available from the UK Data Service: https://discover.ukdataservice.ac.uk/; SN: 7799; persistent identifier: 10.5255/UKDA-SN-77991-1.

For peer review only

REFERENCES

1. Mitchell KR, Mercer CH, Ploubidis GB, et al. Sexual function in Britain: Findings from the third National Survey of Sexual Attitudes and Lifestyles (Natsal-3). *Lancet* 2013;382:1817-1829.

2. Mitchell KR, Jones KG, Wellings K, et al. Estimating the prevalence of sexual function problems: The impact of morbidity criteria. *J Sex Res* 2016;53:55-967.

3. Brotto LA. The DSM diagnostic criteria for hypoactive sexual desire disorder in women. *Arch Sex Behav* 2010a; 39:221-239.

4. Carvalho J, Nobre P. Biopsychosocial determinants of men's sexual desire: testing an integrative model. *J Sex Med* 2011;8:754-763.

5. Štulhofer A, Carvalheira AA, Træen B. Is responsive sexual desire for partnered sex problematic among men? Insights from a two-country study. *Sex Relation Ther* 2013;28:246-258.

6. American Psychiatric Association. *Diagnostic and statistical manual of mental disorders*, 5th. ed. Arlington, VA: Author 2013.

7. Brotto LA. The DSM diagnostic criteria for hypoactive sexual desire disorder in men. *J Sex Med* 2010b;7:2015-2030.

8. Corona G, Lee DM, Forti G, et al. Age-related changes in general and sexual health in middle-aged and older men: Results from the European Male Ageing Study (EMAS). *J Sex Med* 2010;7:1362-1380.

9. DeRogatis L, Rosen RC, Goldstein I, et al. Characterization of hypoactive sexual desire disorder (HSDD) in men. *J Sex Med* 2012;9:812-820.

10. Carvalheira A, Træen B, Štulhofer A. Correlates of men's sexual interest: a cross-cultural study. *J Sex Med* 2014;11:154-164.

For peer review only

11. Corona G, Petrone L, Mannucci E, et al. The impotent couple: low desire. *Int J Androl* 2005;28:46-52.

12. DeLamater JD, Sill M. Sexual desire in later life. *J Sex Res* 2005;42:138-149.

13. Dennerstein L, Koochaki P, Barton I, et al. Hypoactive sexual desire disorder in menopausal women: a survey of Western European women. *J Sex Med* 2006;3:212-222.

14. Hayes RD, Dennerstein L, Bennett CM, et al. Risk factors for female sexual dysfunction in the general population: Exploring factors associated with low sexual function and sexual distress. *J Sex Med* 2008;5:1681-1693.

15. McCabe MP, Goldhammer DL. Demographic and psychological factors related to sexual desire among heterosexual women in a relationship. *J Sex Res* 2012;49:78-87.

16. Öberg K, Sjögren Fugl-Meyer K. On Swedish women's distressing sexual dysfunctions: some concomitant conditions and life satisfaction. *J Sex Med* 2005;2:169-180.

17. Witting K, Santtila P, Varjonen M, et al. Female sexual dysfunction, sexual distress, and compatibility with partner. *J Sex Med* 2008;5:2587-2599.

18. Shifren JL, Monz BU, Russo PA, et al. Sexual problems and distress in United States women. *Obstet Gynecol* 2008;112:970-978.

19. Johannes CB, Clayton AH, Odom DM, et al. Distressing sexual problems in United States women revisited: prevalence after accounting for depression. *J Clin Psychiatry* 2009;70:1698-1706.

For peer review only

20. Mitchell KR, Mercer CH, Wellings K, Johnson AM. Prevalence of low sexual desire among women in Britain: Associated factors. *J Sex Med*. 2009; 1;6(9):2434-2444.

21. Erens B, Phelps A, Clifton S, et al. Methodology of the third British National Survey of Sexual Attitudes and Lifestyles (Natsal-3). *Sex Transm Infect* 2014;90:84-89.

22. Mercer CH, Tanton C, Prah P, et al. Changes in sexual attitudes and lifestyles in Britain through the life course and over time: findings from the National Surveys of Sexual Attitudes and Lifestyles (Natsal). *Lancet* 2013;382:1781-1794.

23. Jones KG, Mitchell KR, Ploubidis GB, et al. The Natsal-SF measure of sexual function: Comparison of three scoring methods. *J Sex Res* 2015;52:640-646.

24. Mitchell KR, Ploubidis GB, Datta J, et al. The Natsal-SF: a validated measure of sexual function for use in community surveys. *Eur J Epidemiol* 2012;27:409-18.

25. Abdo CHN, Oliveira WM, Moreira ED, et al. Prevalence of sexual dysfunctions and correlated conditions in a sample of Brazilian women—results of the Brazilian study on sexual behavior (BSSB). *Int J Impot Res* 2004;16:160-166.

26. Hayes RD, Dennerstein L, Bennett CM, et al. Relationship between hypoactive sexual desire disorder and aging. *Fertil Steril* 2007;87:107-112.

27. Peixoto MM, Nobre P. Prevalence and sociodemographic predictors of sexual problems in Portugal: A population-based study with women aged 18 to 79 years. *J Sex Marital Ther* 2015;41:169-180.

28. Rosen RC, Shifren JL, Monz BU, et al. Correlates of sexually-related personal distress in women with low sexual desire. *J Sex Med* 2009;6:1549-1560.

29. Laumann EO, Glasser DB, Neves RCS, et al. A population-based survey of sexual activity, sexual problems and associated help-seeking behavior patterns in

For peer review only

mature adults in the United States of America. *Int J Impot Res* 2009;21:171-178.

30. Eplov L, Giraldi A, Davidsen M, et al. Sexual desire in a nationally representative Danish population. *J Sex Med* 2007;4:47-56.

31. Mercer CH, Fenton KA, Johnson AM, et al. Who reports sexual function problems? Empirical evidence from Britain's 2000 National Survey of Sexual Attitudes and Lifestyles. *Sex Transm Infect* 2005;81:394-399.

32. Park A, Bryson C, Clery E, et al. *British social attitudes 30*. London: NatCen 2013.

33. Bodenmann G, Ledermann T, Blattner D, et al. Associations among everyday stress, critical life events, and sexual problems. *J of Nerv Ment Dis* 2006;194:494-501.

34. Gerressu M, Mercer CH, Graham CA, et al. Prevalence of masturbation of associated factors in a British national probability survey. *Arch Sex Behav* 2008;37:266-278.

35. Laumann EO, Paik A, Rosen RC. Sexual dysfunctions in the United States: Prevalence and predictors. *JAMA* 1999;281:537-544.

36. Janssen E, McBride KR, Yarber W, et al. Factors that influence sexual arousal in men: A focus group study. *Arch Sex Behav* 2008;37:252-265.

37. Murray S, Milhausen RR, Graham CA, et al. A qualitative exploration of factors that affect sexual desire among men aged 30 to 65 in long-term relationships. *J Sex Res* 2016;doi 10.1080/00224499.2016.1168352

38. Çayan S, Bozlu M, Canpolat B, et al. The assessment of sexual functions in women with male partners complaining of erectile dysfunction: does treatment of

For peer review only

male sexual dysfunction improve female partner's sexual functions? *J Sex Marital Ther* 2004;30:333-341.

39. Fisher WA, Rosen RC, Eardley I, et al. Experience of female partners of men with erectile dysfunction: The female experience of men's attitudes to life events and sexuality (FEMALES) study. *J Sex Med* 2005;2:675-668.

40. Willoughby BJ, Farero AM, Busby DM. Exploring the effects of sexual desire discrepancy among married couples. *Arch Sex Behav* 2014;43:551-562.

41. Hawes ZC, Wellings K, Stephenson J. First heterosexual intercourse in the United Kingdom: A review of the literature. *J Sex Res* 2010;47:137-152.

42. Laumann EO, Nicolosi A, Glasser DB, et al. Sexual problems among women and men aged 40–80 years: Prevalence and correlates identified in the Global Study of Sexual Attitudes and Behaviors. *Int J Impot Res* 2005;17:39-57.

43. Park A, Clery E, Curtice J, et al. *British social attitudes: The 28th report*. London, England: NatCen 2012.

44. Christensen BS, Grønbæk M, Osler M, et al. Sexual dysfunctions and difficulties in Denmark: Prevalence and associated sociodemographic factors. *Arch Sex Behav* 2011;40:121-132.

45. Graham CA, Boynton PM, Gould K. Women's sexual desire: Challenging narratives of dysfunction. *Eur Psychol*, in press.

46. Woloshin S, Schwartz LM. US Food and Drug Administration approval of flibanserin: Even the Score does not add up. *JAMA Intern Med* 2016; doi: 10.1001/jamainternmed.2016.0073

47. Hart G, Wellings K. Sexual behaviour and its medicalisation: In sickness and in health. BMJ 2002;324:896-900.

For peer review only

48. Frühauf S, Gerger H, Schmidt HM, et al. Efficacy of psychological interventions for sexual dysfunction: a systematic review and meta-analysis. *Arch Sex Behav* 2013;42:915-933.

49. Brotto LA, Atallah CS, Johnson-Agbakwu C, et al. Psychological and interpersonal dimensions of sexual function and dysfunction. *J Sex Med* 2016;13:538-571.

50. Payne RA, Abel GA. UK indices of multiple deprivation-a way to make comparisons across constituent countries easier. *Health Stat Q* 2012;53:22-37.

For peer review only

Table 1: Factors associated with lacking interest in having sex for at least 3 months in the past year in sexually active men and women

	Men						Women						
	Denom. (unwt, wt)	%	(95%CI)	Age-adjusted OR	(95%CI)	p-value	*Denom. (unwt, wt)*	%	(95%CI)	Age-adjusted OR	(95%CI)	p-value	*p-value for interaction with sex[a]*
**All**	*4839, 5973*	**15.0%**	**(13.9-16.2)**				*6669, 5755*	**34.2%**	**(32.8-35.5)**				
**Socio-demographics**													
**Age group**						0.0471						<0.0001	*0.6733*
16-24	*1279, 936*	11.5%	(9.4-14.0)	1	-		*1662, 923*	24.8%	(22.5-27.1)	1	-		
25-34	*1376, 1238*	14.6%	(12.7-16.6)	1.32	(1.00-1.73)		*2236, 1246*	31.9%	(29.8-34.1)	1.42	(1.22-1.66)		
35-44	*719, 1298*	17.2%	(14.5-20.4)	1.61	(1.19-2.18)		*1050, 1290*	36.8%	(33.7-40.1)	1.77	(1.48-2.13)		
45-54	*630, 1186*	15.3%	(12.5-18.7)	1.40	(1.01-1.95)		*871, 1186*	37.9%	(34.5-41.5)	1.86	(1.53-2.25)		
55-64	*512, 849*	16.5%	(13.4-20.2)	1.53	(1.10-2.13)		*569, 755*	38.8%	(34.5-43.2)	1.92	(1.55-2.39)		
65-74	*323, 467*	13.9%	(10.4-18.3)	1.22	(0.81-1.82)		*281, 355*	34.2%	(28.4-40.5)	1.58	(1.18-2.12)		
**Index of Multiple Deprivation (quintiles)[b]**						0.093						0.0316	*0.0111*
1 (least deprived)	*977, 1279*	13.9%	(11.6-16.6)	1	-		*1248, 1208*	35.7%	(32.6-38.9)	1	-		
2	*962, 1264*	13.0%	(10.8-15.6)	0.93	(0.69-1.25)		*1290, 1208*	33.6%	(30.6-36.7)	0.92	(0.76-1.13)		
3	*942, 1169*	18.0%	(15.2-21.2)	1.38	(1.04-1.85)		*1299, 1116*	30.1%	(27.2-33.2)	0.81	(0.66-0.99)		
4	*967, 1184*	15.3%	(12.8-18.3)	1.15	(0.86-1.55)		*1384, 1137*	35.9%	(33.0-39.0)	1.08	(0.89-1.30)		
5 (most deprived)	*991, 1077*	15.1%	(12.7-17.8)	1.14	(0.85-1.52)		*1448, 1086*	35.3%	(32.4-38.3)	1.06	(0.87-1.28)		
**Education level[c]**						0.0083						0.2453	0.2914
Left school aged 17+	*2862, 3464*	13.5%	(12.1-15.1)	1	-		*4150, 3406*	32.7%	(31.0-34.5)	1	-		
Left school at 16	*1873, 2437*	17.2%	(15.3-19.4)	1.31	(1.07-1.60)		*2409, 2287*	36.6%	(34.4-38.9)	1.08	(0.95-1.23)		
**Employment status**						0.0086						0.0003	*0.0766*
Employed	*3211, 4254*	14.7%	(13.3-16.1)	1	-		*3871, 3517*	34.6%	(32.9-36.4)	1	-		
Full-time education	*542, 431*	12.6%	(8.8-17.5)	0.98	(0.64-1.51)		*693, 423*	22.5%	(19.0-26.4)	0.70	(0.55-0.89)		
Unemployed	*707, 723*	19.6%	(16.3-23.4)	1.44	(1.12-1.86)		*1681, 1282*	36.1%	(33.4-39.0)	1.11	(0.96-1.28)		
Retired	*375, 562*	13.6%	(10.4-17.7)	0.75	(0.52-1.09)		*415, 524*	35.8%	(31.0-40.9)	0.75	(0.57-0.99)		
**Practises religion at least once a month**						0.1687						0.0082	*0.9966*
No	*4283, 5179*	15.3%	(14.1-16.6)	1	-		*5659, 4754*	34.8%	(33.3-36.3)	1	-		
Yes	*521, 748*	12.9%	(10.0-16.4)	0.81	(0.60-1.09)		*956, 945*	30.7%	(27.5-34.2)	0.79	(0.67-0.94)		

For peer review only

For peer review only - http://bmjopen.bmj.com/site/about/guidelines.xhtml

Table 1 cont.

	Men						Women						
	Denom. (unwt, wt)	%	(95%CI)	Age-adjusted OR	(95%CI)	p-value	*Denom. (unwt, wt)*	%	(95%CI)	Age-adjusted OR	(95%CI)	p-value	*p-value for interaction with sex[a]*
**Health**													
**Self-reported general health**						<0.0001						<0.0001	*0.1890*
Very good/Good	*4123, 5055*	13.4%	(12.2-14.6)	1	-		*5683, 4851*	32.3%	(30.9-33.8)	1	-		
Fair	*580, 745*	21.9%	(18.3-25.8)	1.8	(1.41-2.30)		*780, 709*	42.2%	(38.2-46.3)	1.45	(1.21-1.75)		
Bad/very bad	*135, 171*	33.9%	(25.3-43.6)	3.29	(2.14-5.06)		*206, 195*	49.9%	(42.2-57.7)	1.93	(1.40-2.67)		
**Difficulty walking up stairs because of a health problem**						<0.0001						0.0497	*0.1179*
No difficulty	*4475, 5460*	14.1%	(12.9-15.3)	1	-		*6062, 5107*	33.3%	(31.8-34.7)	1	-		
Some difficulty	*278, 393*	23.0%	(18.1-28.8)	1.8	(1.30-2.49)		*450, 482*	39.2%	(34.4-44.2)	1.15	(0.92-1.43)		
Much difficulty/unable to do this	*86, 120*	30.9%	(20.9-43.0)	2.68	(1.57-4.57)		*157, 166*	47.0%	(38.0-56.1)	1.55	(1.06-2.25)		
**Longstanding illness or disability**						<0.0001						<0.0001	*0.1348*
No	*3585, 4259*	12.8%	(11.6-14.2)	1	-		*4843, 4026*	31.6%	(30.0-33.2)	1	-		
Yes	*1253, 1713*	20.5%	(18.1-23.1)	1.76	(1.44-2.16)		*1825, 1729*	40.1%	(37.5-42.8)	1.35	(1.17-1.55)		
**Number of comorbid conditions[d]**						<0.0001						<0.0001	*0.7951*
0	*3453, 3994*	12.8%	(11.5-14.1)	1	-		*4357, 3536*	29.9%	(28.2-31.5)	1	-		
1	*939, 1329*	18.9%	(16.2-21.9)	1.64	(1.30-2.06)		*1555, 1416*	38.6%	(35.9-41.5)	1.42	(1.23-1.64)		
>=2	*446, 650*	21.0%	(17.0-25.6)	1.91	(1.41-2.60)		*755, 802*	45.1%	(41.2-49.1)	1.75	(1.45-2.13)		
**Depressive symptoms[e]**						<0.0001						<0.0001	*0.6249*
No	*4383, 5471*	13.5%	(12.4-14.8)	1	-		*5885, 5149*	31.7%	(30.2-33.1)	1	-		
Yes	*449, 495*	31.3%	(26.4-36.7)	2.95	(2.26-3.85)		*780, 602*	55.2%	(51.0-59.5)	2.79	(2.32-3.37)		
**Treated for depression, past year**						<0.0001						<0.0001	*0.2447*
No	*4524, 5630*	14.0%	(12.9-15.2)	1	-		*5770, 5040*	31.7%	(30.2-33.2)	1	-		
Yes	*313, 342*	31.5%	(25.7-38.0)	2.82	(2.08-3.83)		*897, 713*	51.4%	(47.6-55.2)	2.32	(1.96-2.75)		

For peer review only

Table 1 cont.

	Men						Women						
	Denom. (unwt, wt)	%	(95%CI)	Age-adjusted OR	(95%CI)	p-value	*Denom. (unwt, wt)*	%	(95%CI)	Age-adjusted OR	(95%CI)	p-value	*p-value for interaction with sex[a]*
**Menopausal status**												0.9326	
Not menopausal							*5485, 4187*	32.3%	(30.9-33.8)	1	-		
Menopausal							*1167, 1548*	38.9%	(36.0-41.9)	0.99	(0.79-1.24)		
**Circumcised**						0.5951							
No	*3909, 4728*	15.1%	(13.8-16.4)	1	-								
Yes	*857, 1166*	14.5%	(12.0-17.4)	0.94	(0.73-1.20)								
**<u>Sexual behaviour</u>**													
**Number of occasions of sex, past 4 weeks**						<0.0001						<0.0001	*0.4778*
0	*1013, 1163*	20.7%	(17.8-23.8)	1	-		*1408, 1245*	42.9%	(39.9-45.9)	1	-		
1-2	*1160, 1566*	18.7%	(16.2-21.5)	0.89	(0.69-1.14)		*1481, 1373*	39.6%	(36.7-42.5)	0.89	(0.75-1.05)		
3-4	*870, 1168*	12.4%	(10.1-15.1)	0.54	(0.41-0.73)		*1240, 1130*	33.8%	(30.7-37.0)	0.7	(0.58-0.85)		
5+	*1617, 1869*	9.2%	(7.8-11.0)	0.39	(0.30-0.51)		*2078, 1655*	22.6%	(20.5-24.8)	0.41	(0.34-0.49)		
**Masturbation, past 4 weeks**						0.0458						0.0038	*0.0005*
No	*1297, 1828*	13.7%	(11.8-15.8)	1	-		*4032, 3612*	36.0%	(34.3-37.7)	1	-		
Yes	*3531, 4132*	15.6%	(14.2-17.0)	1.24	(1.00-1.52)		*2615, 2114*	30.8%	(28.7-33.0)	0.83	(0.73-0.94)		
**No. of sexual partners, past year[f]**						0.5348						0.0038	*0.0183*
1	*3573, 4824*	15.0%	(13.7-16.3)	1	-		*5440, 5012*	35.3%	(33.8-36.8)	1	-		
2	*539, 513*	16.2%	(12.9-20.3)	1.14	(0.86-1.52)		*570, 364*	28.2%	(23.9-32.8)	0.80	(0.63-1.01)		
3+	*718, 627*	13.6%	(11.1-16.6)	0.94	(0.72-1.22)		*642, 366*	24.8%	(21.0-29.0)	0.70	(0.56-0.88)		
**Paid for sex, past year**						0.7167							
No	*4774, 5896*	15.0%	(13.9-16.2)	1	-								
Yes	*64, 75*	13.4%	(6.8-24.7)	0.87	(0.41-1.84)								
**Ever taken drugs to assist sexual performance**						0.0175						0.0666	*0.8967*
No	*4188, 5180*	14.4%	(13.2-15.7)	1	-		*6478, 5624*	34.0%	(32.6-35.4)	1	-		
Yes	*636, 776*	19.0%	(15.7-22.8)	1.36	(1.06-1.76)		*184, 124*	40.0%	(32.0-48.5)	1.39	(0.98-1.96)		

For peer review only

Table 1 cont.

	Men						Women						
	Denom. (unwt, wt)	%	(95%CI)	Age-adjusted OR	(95%CI)	p-value	Denom. (unwt, wt)	%	(95%CI)	Age-adjusted OR	(95%CI)	p-value	*p-value for interaction with sex*[a]
**Relationship context**													
**Relationship status**						0.0383						<0.0001	*0.0001*
Living with partner	*2708, 4266*	15.5%	(14.1-17.1)	1	-		*3967, 4168*	37.9%	(36.3-39.7)	1	-		
In a steady relationship, not living together	*947, 760*	12.0%	(9.6-14.8)	0.76	(0.57-1.00)		*1360, 790*	22.6%	(20.2-25.2)	0.51	(0.43-0.60)		
Not in a steady relationship, but previously cohabited	*446, 388*	18.2%	(14.6-22.5)	1.22	(0.91-1.62)		*752, 462*	28.9%	(25.4-32.8)	0.68	(0.56-0.83)		
Not in a steady relationship, never cohabited	*727, 551*	12.4%	(9.9-15.5)	0.8	(0.58-1.09)		*580, 330*	21.3%	(17.6-25.5)	0.49	(0.38-0.63)		
**Duration of most recent sexual relationship**						0.494						<0.0001	*<0.0001*
1 year or less	*1462, 1260*	13.0%	(11.0-15.3)	1	-		*1597, 998*	21.5%	(19.1-24.1)	1	-		
Between 1 and 5 years	*1247, 1227*	15.3%	(13.2-17.7)	1.21	(0.94-1.55)		*1758, 1148*	28.5%	(26.1-31.0)	1.45	(1.20-1.76)		
Between 5 and 15 years	*1065, 1484*	14.9%	(12.6-17.5)	1.14	(0.86-1.50)		*1774, 1458*	39.8%	(37.2-42.4)	2.37	(1.96-2.86)		
Over 15 years	*1004, 1904*	16.1%	(13.9-18.7)	1.19	(0.87-1.63)		*1445, 2036*	40.0%	(37.3-42.7)	2.31	(1.84-2.91)		
**Always easy to talk about sex with partners**[g]						0.0001						<0.0001	*0.0182*
Yes	*1695, 1899*	11.5%	(9.7-13.5)	1	-		*1746, 1451*	22.6%	(20.4-25.1)	1	-		
No/other	*3122, 4048*	16.7%	(15.3-18.2)	1.53	(1.23-1.90)		*4907, 4289*	38.0%	(36.4-39.6)	2.06	(1.77-2.39)		
**Happy with relationship**[h]						<0.0001						<0.0001	*0.8679*
Yes	*1951, 2791*	12.6%	(11.0-14.4)	1	-		*2736, 2601*	31.5%	(29.5-33.6)	1	-		
Other	*995, 1430*	21.0%	(18.4-23.9)	1.85	(1.47-2.32)		*1640, 1617*	45.4%	(42.7-48.1)	1.79	(1.55-2.08)		
**Participant does not share same level of interest in sex as partner**						0.2339						<0.0001	*<0.0001*
No/other	*2270, 3233*	15.0%	(13.4-16.7)	1	-		*3211, 3064*	27.2%	(25.4-29.0)	1	-		
Yes	*676, 988*	17.1%	(14.2-20.4)	1.17	(0.90-1.51)		*1166, 1155*	62.5%	(59.2-65.7)	4.57	(3.87-5.38)		

For peer review

Table 1 cont.

	Men						Women						
	Denom. (unwt, wt)	%	(95%CI)	Age-adjusted OR	(95%CI)	p-value	*Denom. (unwt, wt)*	%	(95%CI)	Age-adjusted OR	(95%CI)	p-value	*p-value for interaction with sex*[a]
**Participant does not share same sexual likes & dislikes as partner**						0.4188						<0.0001	*<0.0001*
No/other	*2650, 3803*	15.3%	(13.8-16.9)	1	-		*4079, 3908*	34.9%	(33.3-36.6)	1	-		
Yes	*296, 418*	17.3%	(13.0-22.5)	1.16	(0.81-1.66)		*297, 310*	61.0%	(54.6-67.2)	2.91	(2.22-3.83)		
**Partner experienced sexual difficulties in past year**						0.0136						<0.0001	*0.4140*
No/other	*2431, 3454*	14.6%	(13.1-16.2)	1	-		*3726, 3498*	34.8%	(33.1-36.6)	1	-		
Yes	*513, 763*	19.4%	(15.8-23.6)	1.41	(1.07-1.86)		*649, 719*	46.8%	(42.5-51.1)	1.60	(1.32-1.94)		
**Does not feel emotionally close to partner when have sex**						0.0006						<0.0001	*0.5972*
No/other	*2904, 4165*	15.1%	(13.7-16.6)	1	-		*4263, 4108*	35.9%	(34.3-37.6)	1	-		
Yes	*42, 56*	39.9%	(23.6-58.8)	3.74	(1.76-7.93)		*112, 109*	73.0%	(62.8-81.3)	4.80	(2.99-7.69)		
**<u>Lifestyle</u>**													
**1+ child(ren) aged <5 in household**						0.9088						<0.0001	*0.0216*
No, none	*4100, 5015*	15.2%	(13.9-16.5)	1	-		*4997, 4671*	33.1%	(31.6-34.6)	1	-		
Yes, 1+	*727, 941*	14.5%	(11.9-17.6)	0.98	(0.76-1.28)		*1664, 1074*	38.6%	(36.0-41.4)	1.55	(1.34-1.79)		
**Pregnant in the last year**												0.0114	
No							*4227, 4122*	36.2%	(34.6-37.9)	1	-		
Yes							*437, 273*	41.7%	(36.6-47.1)	1.36	(1.07-1.72)		
**Used hormonal contraceptive, past year**												0.05	
No							*3759, 3838*	34.8%	(33.1-36.5)	1	-		
Yes							*2806, 1831*	33.0%	(30.9-35.1)	1.15	(1.00-1.33)		
**<u>Sexual health indicators</u>**													
**Ever diagnosed with a sexually transmitted infection**						<0.0001						0.0004	*0.0651*
No (or only thrush)	*4147, 5127*	14.0%	(12.8-15.3)	1	-		*5455, 4861*	33.4%	(31.9-34.9)	1	-		
Yes (excluding thrush)	*677, 830*	21.4%	(18.1-25.0)	1.67	(1.33-2.10)		*1206, 888*	38.2%	(35.1-41.5)	1.32	(1.13-1.54)		

For peer review only

For peer review only - http://bmjopen.bmj.com/site/about/guidelines.xhtml

Table 1 cont.

	Men						Women						
	Denom. (unwt, wt)	%	(95%CI)	Age-adjusted OR	(95%CI)	p-value	*Denom. (unwt, wt)*	%	(95%CI)	Age-adjusted OR	(95%CI)	p-value	*p-value for interaction with sex[a]*
**Ever experienced non-volitional sex**						0.0010						<0.0001	*0.3164*
No	*4705, 5824*	14.7%	(13.6-16.0)	1	-		*5815, 5055*	32.8%	(31.4-34.2)	1	-		
Yes/Don't know	*133, 148*	26.1%	(18.9-34.9)	2.07	(1.34-3.18)		*848, 695*	44.3%	(40.5-48.3)	1.66	(1.40-1.97)		
**Sexual competence at first sex[i]**						0.0706						<0.0001	*0.1797*
Not competent	*2407, 3037*	16.2%	(14.6-17.9)	1	-		*3438, 2927*	37.6%	(35.7-39.5)	1	-		
Competent	*2302, 2784*	13.7%	(12.1-15.4)	0.84	(0.69-1.01)		*3097, 2716*	30.3%	(28.4-32.3)	0.73	(0.65-0.83)		
**Number of other sexual response problems experienced[j]**						<0.0001						<0.0001	*0.0015*
0	*3208, 3945*	11.7%	(10.5-13.1)	1	-		*4377, 3759*	25.3%	(23.8-26.9)	1	-		
1	*1061, 1350*	10.9%	(9.0-13.2)	0.91	(0.71-1.17)		*1217, 1087*	34.8%	(31.7-38.0)	1.55	(1.32-1.82)		
2+	*570, 678*	42.5%	(37.9-47.2)	5.58	(4.41-7.04)		*1075, 909*	69.8%	(66.5-72.9)	6.91	(5.82-8.21)		
**<u>Attitudes</u>**													
**People are under pressure to have sex**						0.0115						0.0001	*0.7970*
Else	*1799, 2264*	13.1%	(11.4-15.0)	1	-		*1851, 1570*	29.3%	(26.8-31.9)	1	-		
Strongly agree/agree	*3038, 3707*	16.2%	(14.7-17.8)	1.29	(1.06-1.57)		*4817, 4185*	36.0%	(34.4-37.6)	1.34	(1.16-1.54)		
**People want less sex as they age**						<0.0001						<0.0001	*0.9443*
Else	*2943, 3472*	11.4%	(10.2-12.8)	1	-		*4044, 3278*	27.8%	(26.2-29.4)	1	-		
Strongly agree/agree	*1894, 2499*	20.0%	(18.0-22.2)	1.93	(1.61-2.32)		*2624, 2477*	42.6%	(40.4-44.8)	1.85	(1.63-2.10)		
**Men have a naturally higher sex drive than women**						<0.0001						<0.0001	*<0.0001*
Else	*2788, 3441*	18.0%	(16.4-19.7)	1	-		*3351, 2830*	26.0%	(24.3-27.8)	1	-		
Strongly agree/agree	*2049, 2530*	10.9%	(9.4-12.6)	0.56	(0.46-0.68)		*3317, 2925*	42.0%	(40.0-44.1)	2.04	(1.80-2.31)		
**Too much sex in the media**						0.7069						0.1807	*0.4835*
Else	*1986, 2296*	14.6%	(12.8-16.6)	1	-		*2091, 1618*	31.7%	(29.3-34.2)	1	-		
Strongly agree/agree	*2851, 3675*	15.3%	(13.8-16.9)	1.04	(0.85-1.26)		*4577, 4137*	35.1%	(33.5-36.8)	1.10	(0.96-1.26)		

For peer review only

For peer review only - http://bmjopen.bmj.com/site/about/guidelines.xhtml

Denominator is those aged 16-74 years with at least one partner in the past year. Unwt, unweighted; wt, weighted

[a] P-value for interaction to determine whether the magnitude of association between each variable and lack of interest in sex differs between men and women

[b] IMD is a multidimensional measure of area (neighbourhood)-level deprivation based on the participant's postcode. IMD scores for England, Scotland and Wales were adjusted before being combined and assigned to quintiles, using a method by Payne and Abel (50).

[c] Participants aged ≥17 years.

[d] Includes arthritis, heart attack, coronary heart disease, angina, other forms of heart disease, hypertension, stroke, diabetes, broken hip or pelvis, bone or hip replacement ever, backache lasting longer than 3 months, any other muscle or bone disease lasting longer than 3 months, depression, cancer and any thyroid condition treated in the past year.

[e] Participants were asked whether they had often been bothered by feeling down, depressed, or hopeless in the past 2 weeks, and whether they had often been bothered by little interest or pleasure in doing things in the past 2 weeks, using a validated two-question patient health questionnaire (PHQ-2).

[f] Opposite and/or same-sex partners

[g] Other means easy with a husband or wife or regular partner, but difficult with a new partner; easy with a new partner, but difficult with a husband or wife or regular partner; difficult with any partner, it depends, sometimes easy, and sometimes difficult.

[h] Participants were asked to rate how happy they were in their relationship from 1 (very happy) to 7 (very unhappy); responses of 1 or 2 were regarded as denoting participants who were happy with their relationship.

[i] A constructed variable to measure readiness, combining consensuality, autonomy of decision making, timing and use of effective contraception

[j] Sexual response problems (for at least 3 months in past year): lacked enjoyment in sex, felt anxious during sex, felt physical pain as a result of sex, felt no excitement or arousal during sex, difficulty in reaching climax, reached a climax more quickly than you would like, trouble getting or keep an erection (men), uncomfortably dry vagina (women)

For peer review only

Table 2: Factors associated with lacking interest in having sex for at least 3 months in the past year and being distressed about it in sexually active men and women

	Men						Women						
	Denom. (unwt, wt)	%	(95%CI)	Age-adjusted OR	(95%CI)	p-value	*Denom. (unwt, wt)*	%	(95%CI)	Age-adjusted OR	(95%CI)	p-value	*p-value for interaction with sex[a]*
**All**	*4839, 5973*	**8.2%**	**(7.4-9.1)**				*6669, 5755*	**20.8%**	**(19.6-22.0)**				
**Socio-demographics**													
**Age group**						0.0011						<0.0001	*0.8971*
16-24	*1279, 936*	4.8%	(3.7-6.4)	1	-		*1662, 923*	15.2%	(13.4-17.3)	1	-		
25-34	*1376, 1238*	8.0%	(6.7-9.5)	1.7	(1.19-2.41)		*2236, 1246*	20.9%	(19.0-22.8)	1.47	(1.22-1.76)		
35-44	*719, 1298*	9.6%	(7.5-12.3)	2.09	(1.40-3.13)		*1050, 1290*	22.9%	(20.3-25.7)	1.65	(1.34-2.04)		
45-54	*630, 1186*	9.7%	(7.4-12.6)	2.11	(1.38-3.22)		*871, 1186*	23.3%	(20.4-26.6)	1.69	(1.35-2.13)		
55-64	*512, 849*	9.4%	(7.0-12.6)	2.04	(1.30-3.21)		*569, 755*	21.8%	(18.3-25.8)	1.55	(1.20-2.01)		
65-74	*323, 467*	5.5%	(3.4-8.6)	1.13	(0.65-1.99)		*281, 355*	16.5%	(12.4-21.7)	1.10	(0.76-1.59)		
**Index of Multiple Deprivation (quintiles)[b]**						0.8339						0.0938	*0.4592*
1 (least deprived)	*977, 1279*	8.1%	(6.2-10.4)	1	-		*1248, 1208*	23.3%	(20.7-26.1)	1	-		
2	*962, 1264*	7.4%	(5.7-9.6)	0.92	(0.62-1.36)		*1290, 1208*	20.8%	(18.2-23.5)	0.87	(0.69-1.09)		
3	*942, 1169*	8.3%	(6.4-10.6)	1.05	(0.71-1.55)		*1299, 1116*	19.6%	(17.1-22.4)	0.82	(0.65-1.03)		
4	*967, 1184*	8.8%	(6.9-11.1)	1.14	(0.78-1.66)		*1384, 1137*	21.9%	(19.3-24.7)	0.95	(0.76-1.18)		
5 (most deprived)	*991, 1077*	8.6%	(6.7-10.9)	1.12	(0.75-1.65)		*1448, 1086*	18.2%	(15.9-20.6)	0.75	(0.60-0.94)		
**Education level[c]**						0.4958						0.7324	0.4496
Left school aged 17+	*2862, 3464*	7.9%	(6.8-9.2)	1	-		*4150, 3406*	20.8%	(19.4-22.4)	1	-		
Left school at 16	*1873, 2437*	8.8%	(7.5-10.4)	1.09	(0.85-1.41)		*2409, 2287*	21.1%	(19.3-23.1)	0.97	(0.83-1.14)		
**Employment status**						0.0001						0.0003	*0.1244*
Employed	*3211, 4254*	8.3%	(7.3-9.5)	1	-		*3871, 3517*	21.6%	(20.1-23.2)	1	-		
Full-time education	*542, 431*	4.9%	(2.8-8.6)	0.74	(0.38-1.44)		*693, 423*	14.8%	(11.8-18.4)	0.75	(0.56-1.01)		
Unemployed	*707, 723*	12.1%	(9.5-15.3)	1.56	(1.14-2.13)		*1681, 1282*	22.3%	(19.9-24.9)	1.07	(0.90-1.27)		
Retired	*375, 562*	4.9%	(3.1-7.6)	0.41	(0.23-0.71)		*415, 524*	16.8%	(13.4-20.8)	0.57	(0.41-0.79)		
**Practises religion at least once a month**						0.1638						0.0167	*0.8143*
No	*4283, 5179*	8.5%	(7.5-9.5)	1	-		*5659, 4754*	21.5%	(20.2-22.9)	1	-		
Yes	*521, 748*	6.4%	(4.4-9.4)	0.73	(0.48-1.13)		*956, 945*	18.0%	(15.3-20.9)	0.78	(0.63-0.96)		

For peer review only

Table 2 cont.

	Men						Women						
	Denom. (unwt, wt)	%	(95%CI)	Age-adjusted OR	(95%CI)	p-value	*Denom. (unwt, wt)*	%	(95%CI)	Age-adjusted OR	(95%CI)	p-value	*p-value for interaction with sex[a]*
**Health**													
**Self-reported general health**						<0.0001						<0.0001	*0.0969*
Very good/Good	*4123, 5055*	7.0%	(6.1-7.9)	1	-		*5683, 4851*	19.2%	(18.0-20.5)	1	-		
Fair	*580, 745*	13.3%	(10.5-16.8)	2.04	(1.50-2.78)		*780, 709*	27.9%	(24.3-31.9)	1.60	(1.30-1.97)		
Bad/very bad	*135, 171*	22.6%	(15.3-32.1)	3.85	(2.31-6.40)		*206, 195*	33.4%	(26.3-41.4)	2.05	(1.45-2.91)		
**Difficulty walking up stairs because of a health problem**						0.0001						0.0085	*0.1553*
No difficulty	*4475, 5460*	7.6%	(6.7-8.6)	1	-		*6062, 5107*	20.1%	(18.9-21.4)	1	-		
Some difficulty	*278, 393*	12.3%	(8.8-17.0)	1.67	(1.11-2.52)		*450, 482*	24.1%	(20.0-28.7)	1.21	(0.93-1.59)		
Much difficulty/unable to do this	*86, 120*	22.2%	(13.5-34.2)	3.36	(1.79-6.32)		*157, 166*	32.3%	(24.3-41.5)	1.81	(1.21-2.70)		
**Longstanding illness or disability**						<0.0001						<0.0001	*0.0345*
No	*3585, 4259*	6.5%	(5.6-7.5)	1	-		*4843, 4026*	18.7%	(17.4-20.0)	1	-		
Yes	*1253, 1713*	12.5%	(10.6-14.8)	2.09	(1.60-2.74)		*1825, 1729*	25.7%	(23.4-28.2)	1.48	(1.27-1.74)		
**Number of comorbid conditions[d]**						<0.0001						<0.0001	*0.5779*
0	*3453, 3994*	6.4%	(5.5-7.5)	1	-		*4357, 3536*	17.3%	(15.9-18.7)	1	-		
1	*939, 1329*	11.0%	(9.0-13.4)	1.88	(1.37-2.57)		*1555, 1416*	24.1%	(21.7-26.7)	1.54	(1.30-1.83)		
>=2	*446, 650*	13.3%	(10.1-17.4)	2.40	(1.61-3.59)		*755, 802*	30.5%	(26.8-34.4)	2.16	(1.74-2.69)		
**Depressive symptoms[e]**						<0.0001						<0.0001	*0.0370*
No	*4383, 5471*	6.8%	(6.0-7.7)	1	-		*5885, 5149*	18.6%	(17.4-19.8)	1	-		
Yes	*449, 495*	23.7%	(19.3-28.9)	4.36	(3.20-5.94)		*780, 602*	39.6%	(35.4-44.0)	2.94	(2.41-3.59)		
**Treated for depression, past year**						<0.0001						<0.0001	*0.0371*
No	*4524, 5630*	7.3%	(6.5-8.2)	1	-		*5770, 5040*	18.5%	(17.3-19.8)	1	-		
Yes	*313, 342*	23.0%	(17.9-29.1)	3.81	(2.71-5.36)		*897, 713*	36.4%	(32.9-40.2)	2.54	(2.12-3.03)		

For peer review only

Table 2 cont.

	Men						Women						
	Denom. (unwt, wt)	%	(95%CI)	Age-adjusted OR	(95%CI)	p-value	Denom. (unwt, wt)	%	(95%CI)	Age-adjusted OR	(95%CI)	p-value	*p-value for interaction with sex*[a]
**Menopausal status**												0.9656	
Not menopausal							*5485, 4187*	20.2%	(18.9-21.5)	1	-		
Menopausal							*1167, 1548*	22.5%	(20.0-25.2)	1.01	(0.76-1.32)		
**Circumcised**						0.4097							
No	*3909, 4728*	8.3%	(7.4-9.4)	1	-								
Yes	*857, 1166*	7.5%	(5.7-9.9)	0.87	(0.62-1.22)								
**Sexual behaviour**													
**Number of occasions of sex, past 4 weeks**						<0.0001						<0.0001	*0.5496*
0	*1013, 1163*	10.3%	(8.3-12.7)	1	-		*1408, 1245*	23.2%	(20.7-26.0)	1	-		
1-2	*1160, 1566*	10.5%	(8.6-12.8)	1.02	(0.74-1.42)		*1481, 1373*	24.2%	(21.8-26.9)	1.06	(0.87-1.30)		
3-4	*870, 1168*	7.4%	(5.6-9.8)	0.71	(0.48-1.04)		*1240, 1130*	21.3%	(18.7-24.2)	0.91	(0.73-1.13)		
5+	*1617, 1869*	5.0%	(3.9-6.4)	0.46	(0.33-0.66)		*2078, 1655*	14.7%	(12.9-16.7)	0.58	(0.47-0.72)		
**Masturbation, past 4 weeks**						0.0164						0.7265	*0.0309*
No	*1297, 1828*	6.9%	(5.5-8.6)	1	-		*4032, 3612*	21.1%	(19.6-22.6)	1	-		
Yes	*3531, 4132*	8.8%	(7.7-9.9)	1.42	(1.07-1.88)		*2615, 2114*	20.3%	(18.4-22.2)	0.97	(0.84-1.13)		
**No. of sexual partners, past year**[f]						0.2466						0.0016	*0.4744*
1	*3573, 4824*	8.5%	(7.5-9.6)	1	-		*5440, 5012*	21.6%	(20.3-22.9)	1	-		
2	*539, 513*	6.3%	(4.3-9.1)	0.75	(0.49-1.14)		*570, 364*	16.7%	(13.3-20.6)	0.75	(0.57-0.99)		
3+	*718, 627*	6.8%	(5.1-9.0)	0.82	(0.59-1.15)		*642, 366*	14.1%	(11.0-17.7)	0.62	(0.46-0.83)		
**Paid for sex, past year**						0.4865							
No	*4774, 5896*	8.2%	(7.4-9.2)	1	-								
Yes	*64, 75*	5.6%	(1.8-16.4)	0.66	(0.20-2.15)								
**Ever taken drugs to assist sexual performance**						0.0022						0.1055	*0.5305*
No	*4188, 5180*	7.6%	(6.7-8.6)	1	-		*6478, 5624*	20.6%	(19.5-21.8)	1	-		
Yes	*636, 776*	12.1%	(9.5-15.4)	1.63	(1.19-2.23)		*184, 124*	25.9%	(19.2-33.9)	1.38	(0.93-2.05)		

For peer review only

Table 2 cont.

	Men						Women						
	Denom. (unwt, wt)	%	(95%CI)	Age-adjusted OR	(95%CI)	p-value	Denom. (unwt, wt)	%	(95%CI)	Age-adjusted OR	(95%CI)	p-value	*p-value for interaction with sex[a]*
**Relationship context**													
**Relationship status**						0.03						<0.0001	*0.0307*
Living with partner	*2708, 4266*	8.8%	(7.7-10.1)	1	-		*3967, 4168*	23.4%	(21.9-24.9)	1	-		
In a steady relationship, not living together	*947, 760*	6.9%	(5.3-9.0)	0.78	(0.56-1.09)		*1360, 790*	15.4%	(13.4-17.7)	0.59	(0.49-0.71)		
Not in a steady relationship, but previously cohabited	*446, 388*	8.8%	(6.2-12.2)	1.00	(0.67-1.48)		*752, 462*	13.6%	(11.1-16.6)	0.51	(0.40-0.66)		
Not in a steady relationship, never cohabited	*727, 551*	4.7%	(3.3-6.8)	0.52	(0.34-0.81)		*580, 330*	11.0%	(8.2-14.5)	0.39	(0.28-0.55)		
**Duration of most recent sexual relationship**						0.0143						<0.0001	*0.0719*
1 year or less	*1462, 1260*	5.5%	(4.3-7.1)	1	-		*1597, 998*	11.2%	(9.4-13.2)	1	-		
Between 1 and 5 years	*1247, 1227*	9.0%	(7.3-11.0)	1.67	(1.18-2.36)		*1758, 1148*	18.5%	(16.5-20.7)	1.81	(1.44-2.29)		
Between 5 and 15 years	*1065, 1484*	9.3%	(7.5-11.6)	1.68	(1.17-2.43)		*1774, 1458*	25.2%	(23.0-27.6)	2.81	(2.23-3.55)		
Over 15 years	*1004, 1904*	8.8%	(7.1-10.8)	1.47	(0.97-2.22)		*1445, 2036*	23.8%	(21.5-26.2)	2.83	(2.13-3.75)		
**Always easy to talk about sex with partners[g]**						0						<0.0001	*0.4854*
Yes	*1695, 1899*	4.8%	(3.8-6.0)	1	-		*1746, 1451*	11.4%	(9.7-13.2)	1	-		
No/other	*3122, 4048*	9.8%	(8.7-11.1)	2.15	(1.62-2.87)		*4907, 4289*	23.9%	(22.5-25.3)	2.43	(2.02-2.93)		
**Happy with relationship[h]**						<0.000 1						<0.0001	*0.9717*
Yes	*1951, 2791*	7.1%	(5.9-8.6)	1	-		*2736, 2601*	18.6%	(16.9-20.4)	1	-		
Other	*995, 1430*	13.3%	(11.2-15.8)	2.01	(1.51-2.66)		*1640, 1617*	31.4%	(28.8-34.0)	2.00	(1.69-2.37)		
**Participant does not share same level of interest in sex as partner**							0.0311						<0.0001
No/other	*2270, 3233*	8.5%	(7.2-10.0)	1	-		*3211, 3064*	15.0%	(13.6-16.4)	1	-		
Yes	*676, 988*	11.6%	(9.2-14.4)	1.41	(1.03-1.92)		*1166, 1155*	46.2%	(42.9-49.6)	4.91	(4.13-5.83)		

For peer review only

Table 2 cont.

	Men						Women						
	Denom. (unwt, wt)	%	(95%CI)	Age-adjusted OR	(95%CI)	p-value	*Denom. (unwt, wt)*	%	(95%CI)	Age-adjusted OR	(95%CI)	p-value	*p-value for interaction with sex[a]*
**Participant does not share same sexual likes & dislikes as partner**						0.0975						<0.0001	*0.0212*
No/other	*2650, 3803*	8.9%	(7.7-10.2)	1	-		*4079, 3908*	22.1%	(20.6-23.6)	1	-		
Yes	*296, 418*	12.2%	(8.6-17.0)	1.43	(0.94-2.18)		*297, 310*	41.9%	(35.6-48.6)	2.55	(1.93-3.37)		
**Partner experienced sexual difficulties in past year**						0.0027						<0.0001	*0.6889*
No/other	*2431, 3454*	8.3%	(7.2-9.6)	1	-		*3726, 3498*	22.1%	(20.6-23.7)	1	-		
Yes	*513, 763*	13.2%	(10.2-17.0)	1.68	(1.20-2.35)		*649, 719*	30.4%	(26.5-34.6)	1.58	(1.27-1.95)		
**Does not feel emotionally close to partner when have sex**						0.0225						<0.0001	*0.8228*
No/other	*2904, 4165*	9.1%	(7.9-10.3)	1	-		*4263, 4108*	22.9%	(21.5-24.4)	1	-		
Yes	*42, 56*	21.0%	(10.2-38.3)	2.69	(1.15-6.29)		*112, 109*	47.0%	(36.4-57.8)	2.98	(1.92-4.63)		
**<u>Lifestyle</u>**													
**1+ child(ren) aged <5 in household**						0.1047						0.0004	*0.0042*
No, none	*4100, 5015*	8.6%	(7.6-9.6)	1	-		*4997, 4671*	20.2%	(18.9-21.5)	1	-		
Yes, 1+	*727, 941*	6.3%	(4.6-8.5)	0.75	(0.52-1.06)		*1664, 1074*	23.5%	(21.2-25.9)	1.34	(1.14-1.58)		
**Pregnant in the last year**												0.5927	
No							*4227, 4122*	21.8%	(20.4-23.4)	1	-		
Yes							*437, 273*	20.7%	(16.6-25.6)	0.92	(0.69-1.24)		
**Used hormonal contraceptive, past year**												0.1141	
No							*3759, 3838*	20.7%	(19.2-22.3)	1	-		
Yes							*2806, 1831*	20.9%	(19.1-22.7)	1.14	(0.97-1.35)		
**<u>Sexual health indicators</u>**													
**Ever diagnosed with a sexually transmitted infection**						<0.0001						0.0002	*0.0291*
No (or only thrush)	*4148, 5128*	7.3%	(6.5-8.3)	1	-		*5455, 4861*	20.0%	(18.7-21.3)	1	-		
Yes (excluding thrush)	*677, 830*	13.7%	(11.0-17.0)	2.02	(1.51-2.70)		*1206, 888*	25.1%	(22.3-28.1)	1.39	(1.16-1.65)		

For peer review only

Table 2 cont.

	Men						Women						
	Denom. (unwt, wt)	%	(95%CI)	Age-adjusted OR	(95%CI)	p-value	*Denom. (unwt, wt)*	%	(95%CI)	Age-adjusted OR	(95%CI)	p-value	*p-value for interaction with sex[a]*
**Ever experienced non-volitional sex**						<0.0001						<0.0001	*0.1143*
No	*4706, 5825*	7.9%	(7.1-8.9)	1	-		*5815, 5055*	19.4%	(18.2-20.7)	1	-		
Yes/Don't know	*133, 148*	19.4%	(13.1-27.7)	2.83	(1.74-4.59)		*848, 695*	30.9%	(27.3-34.6)	1.86	(1.55-2.25)		
**Sexual competence at first sex[i]**						0.4876						<0.0001	*0.0787*
Not competent	*2408, 3039*	8.7%	(7.5-10.0)	1	-		*3438, 2927*	23.6%	(21.9-25.3)	1	-		
Competent	*2302, 2784*	7.8%	(6.6-9.2)	0.91	(0.71-1.18)		*3097, 2716*	17.7%	(16.1-19.3)	0.70	(0.61-0.81)		
**Number of other sexual response problems experienced[j]**						<0.0001						<0.0001	*0.0262*
0	*3209, 3947*	5.3%	(4.4-6.3)	1	-		*4377, 3759*	12.9%	(11.7-14.1)	1	-		
1	*1061, 1350*	6.1%	(4.7-7.8)	1.14	(0.81-1.59)		*1217, 1087*	21.7%	(19.0-24.6)	1.86	(1.53-2.26)		
2+	*570, 678*	29.7%	(25.4-34.4)	7.57	(5.68-10.10)		*1075, 909*	52.4%	(48.9-56.0)	7.48	(6.25-8.94)		
**Attitudes**													
**People are under pressure to have sex**						0.1437						<0.0001	*0.2192*
Else	*1799, 2264*	7.4%	(6.0-9.0)	1	-		*1851, 1570*	16.4%	(14.5-18.5)	1	-		
Strongly agree/agree	*3038, 3707*	8.7%	(7.6-9.9)	1.21	(0.94-1.57)		*4817, 4185*	22.4%	(21.0-23.9)	1.47	(1.24-1.74)		
**People want less sex as they age**						0.0005						<0.0001	*0.8045*
Else	*2943, 3472*	6.7%	(5.7-7.8)	1	-		*4044, 3278*	17.1%	(15.8-18.6)	1	-		
Strongly agree/agree	*1894, 2499*	10.3%	(8.8-12.1)	1.58	(1.22-2.04)		*2624, 2477*	25.6%	(23.7-27.6)	1.64	(1.43-1.90)		
**Men have a naturally higher sex drive than women**							<0.0001						<0.0001
Else	*2788, 3441*	10.2%	(8.9-11.5)	1	-		*3351, 2830*	15.9%	(14.4-17.4)	1	-		
Strongly agree/agree	*2049, 2530*	5.5%	(4.4-6.9)	0.52	(0.39-0.68)		*3317, 2925*	25.5%	(23.8-27.4)	1.81	(1.56-2.09)		
**Too much sex in the media**						0.3477						0.0693	*0.8856*
Else	*1986, 2296*	7.5%	(6.3-9.0)	1	-		*2091, 1618*	18.8%	(16.8-20.9)	1	-		
Strongly agree/agree	*2851, 3675*	8.6%	(7.5-9.9)	1.13	(0.88-1.46)		*4577, 4137*	21.6%	(20.2-23.0)	1.16	(0.99-1.36)		

Denominator is those aged 16-74 years with at least one partner in the past year. Unwt, unweighted; wt, weighted

[a] P-value for interaction to determine whether the magnitude of association between each variable and lack of interest in sex differs between men and women

For peer review only

[b] IMD is a multidimensional measure of area (neighbourhood)-level deprivation based on the participant's postcode. IMD scores for England, Scotland and Wales were adjusted before being combined and assigned to quintiles, using a method by Payne and Abel (50).

[c] Participants aged ≥17 years.

[d] Includes arthritis, heart attack, coronary heart disease, angina, other forms of heart disease, hypertension, stroke, diabetes, broken hip or pelvis, bone or hip replacement ever, backache lasting longer than 3 months, any other muscle or bone disease lasting longer than 3 months, depression, cancer and any thyroid condition treated in the past year.

[e] Participants were asked whether they had often been bothered by feeling down, depressed, or hopeless in the past 2 weeks, and whether they had often been bothered by little interest or pleasure in doing things in the past 2 weeks, using a validated two-question patient health questionnaire (PHQ-2).

[f] Opposite and/or same-sex partners

[g] Other means easy with a husband or wife or regular partner, but difficult with a new partner; easy with a new partner, but difficult with a husband or wife or regular partner; difficult with any partner, it depends, sometimes easy, and sometimes difficult.

[h] Participants were asked to rate how happy they were in their relationship from 1 (very happy) to 7 (very unhappy); responses of 1 or 2 were regarded as denoting participants who were happy with their relationship.

[i] A constructed variable to measure readiness, combining consensuality, autonomy of decision making, timing and use of effective contraception

[j] Sexual response problems (for at least 3 months in past year): lacked enjoyment in sex, felt anxious during sex, felt physical pain as a result of sex, felt no excitement or arousal during sex, difficulty in reaching climax, reached a climax more quickly than you would like, trouble getting or keep an erection (men), uncomfortably dry vagina (women)

For peer review only

Table 3: Associations between reporting lack of interest in having sex for at least 3 months in the past year and other sexual response problems lasting 3 months or more in the past year, by sex

		Men							Women						
		Did not report a lack interest in sex		Reported a lack of interest in sex			(95%CI)	p-value	Did not report a lack of interest in sex		Reported a lack of interest in sex			(95%CI)	p-value
Denominators (unwt, wt)		4126, 5077		713, 897		AOR[a]			4540, 3790		2129, 1965		AOR[a]		
**Lacked enjoyment in having sex**								<0.0001							<0.0001
	No	97.7%	(97.1-98.1)	81.5%	(78.2-84.4)	1	-		95.9%	(95.1-96.5)	72.5%	(70.2-74.7)	1	-	
	Yes	2.3%	(1.9-2.9)	18.5%	(15.6-21.8)	9.78	(7.11-13.46)		4.1%	(3.5-4.9)	27.5%	(25.3-29.8)	8.95	(7.28-11.01)	
**Felt anxious during sex**								<0.0001							<0.0001
	No	96.1%	(95.5-96.7)	85.8%	(82.6-88.5)	1	-		97.3%	(96.7-97.7)	89.9%	(88.4-91.3)	1	-	
	Yes	3.9%	(3.3-4.5)	14.2%	(11.5-17.4)	4.16	(3.08-5.62)		2.7%	(2.3-3.3)	10.1%	(8.7-11.6)	4.4	(3.43-5.65)	
**Felt physical pain as a result of sex**								0.0213							<0.0001
	No	98.4%	(97.9-98.8)	97.1%	(95.6-98.1)	1	-		95.7%	(95.0-96.3)	86.5%	(84.6-88.1)	1	-	
	Yes	1.6%	(1.2-2.1)	2.9%	(1.9-4.4)	1.87	(1.10-3.19)		4.3%	(3.7-5.0)	13.5%	(11.9-15.4)	3.55	(2.83-4.45)	
**Felt no excitement or arousal during sex**								<0.0001							<0.0001
	No	98.5%	(98.0-98.9)	87.7%	(85.0-90.0)	1	-		97.5%	(96.9-97.9)	80.9%	(79.0-82.7)	1	-	
	Yes	1.5%	(1.1-2.0)	12.3%	(10.0-15.0)	9.21	(6.33-13.40)		2.5%	(2.1-3.1)	19.1%	(17.3-21.0)	9.16	(7.16-11.70)	
**Difficulty in reaching climax**								<0.0001							<0.0001
	No	92.7%	(91.7-93.5)	80.5%	(76.6-83.8)	1	-		88.3%	(87.2-89.3)	74.9%	(72.7-76.9)	1	-	
	Yes	7.3%	(6.5-8.3)	19.5%	(16.2-23.4)	3.08	(2.37-3.99)		11.7%	(10.7-12.8)	25.1%	(23.1-27.3)	2.6	(2.23-3.03)	
**Reached climax more quickly than you would like**								0.0198							0.3658
	No	85.6%	(84.3-86.9)	82.0%	(78.7-85.0)	1	-		97.8%	(97.2-98.2)	97.5%	(96.7-98.1)	1	-	
	Yes	14.4%	(13.1-15.7)	18.0%	(15.0-21.3)	1.32	(1.05-1.68)		2.2%	(1.8-2.8)	2.5%	(1.9-3.3)	1.18	(0.82-1.69)	
**Trouble getting or keeping an erection**								<0.0001							
	No	88.5%	(87.3-89.6)	79.4%	(75.9-82.6)	1	-								
	Yes	11.5%	(10.4-12.7)	20.6%	(17.4-24.1)	1.97	(1.55-2.51)								
**Uncomfortably dry vagina**															<0.0001
	No								90.7%	(89.5-91.7)	80.1%	(77.9-82.1)	1	-	
	Yes								9.3%	(8.3-10.5)	19.9%	(17.9-22.1)	2.28	(1.89-2.76)	

Denominator is those aged 16-74 years with at least one partner in the past year. Unwt, unweighted; wt, weighted

[a]AOR comparing those reporting lacking interest to those who did not.

For peer review only

STROBE Statement—checklist of items that should be included in reports of observational studies

	Item No.	Recommendation	Page No.	Relevant text from manuscript
**Title and abstract**	1	(*a*) Indicate the study's design with a commonly used term in the title or the abstract	Title, Abstract	Survey; cross-sectional probability sample survey
		(*b*) Provide in the abstract an informative and balanced summary of what was done and what was found	Abstract	Complex survey analyses of data collected for a cross-sectional probability sample survey, undertaken 2010-12, specifically logistic regression to calculate age-adjusted odds ratios (AOR) to identify associated factors
**Introduction**				
Background/rationale	2	Explain the scientific background and rationale for the investigation being reported	4-5	In summary, the evidence on the factors associated with men's and women's reports of low sexual desire is drawn largely from non-representative samples, is somewhat equivocal and, in men, sparse. Understanding the correlates of lacking interest in sex would be useful to identify individuals most at risk of sexual problems and could also inform what types of therapeutic options for this group should be provided.
Objectives	3	State specific objectives, including any prespecified hypotheses	5	The research questions addressed in this paper are: (1)

For peer review only - http://bmjopen.bmj.com/site/about/guidelines.xhtml

For peer review only

				What sociodemographic, relationship, sexual behaviour, and sexual attitudinal factors are associated with lacking interest in sex in sexually active men and women?; (2) To what extent do these factors vary by gender?; (3) What are the associations between reporting lacking interest in sex and other sexual function problems?
**Methods**				
Study design	4	Present key elements of study design early in the paper	6	A multi-stage, clustered, and stratified probability sample design was used and participants were interviewed using a combination of computer-assisted personal interviews (CAPI), and computer-assisted self-interviews (CASI).
Setting	5	Describe the setting, locations, and relevant dates, including periods of recruitment, exposure, follow-up, and data collection	6	Natsal-3 is a probability sample survey of 15,162 men and women aged 16-74 years in Britain, interviewed between September 2010 and August 2012.
Participants	6	(*a*) *Cohort study*—Give the eligibility criteria, and the sources and methods of selection of participants. Describe methods of follow-up<br>*Case-control study*—Give the eligibility criteria, and the sources and methods of case	6	For the current analyses, only respondents who reported >=1 sexual partner (opposite-sex or

For peer review only - http://bmjopen.bmj.com/site/about/guidelines.xhtml

		ascertainment and control selection. Give the rationale for the choice of cases and controls<br>*Cross-sectional study*—Give the eligibility criteria, and the sources and methods of selection of participants		same-sex) in the past year were included (4,839 men and 6,669 women).
		(*b*) *Cohort study*—For matched studies, give matching criteria and number of exposed and unexposed<br>*Case-control study*—For matched studies, give matching criteria and the number of controls per case		
Variables	7	Clearly define all outcomes, exposures, predictors, potential confounders, and effect modifiers. Give diagnostic criteria, if applicable	6-7	We used selected items from the Natsal-SF, a newly developed and validated measure of sexual function comprising questions about problems with sexual response, relational aspects of sexual function, and self-appraisal of sex life
Data sources/ measurement	8*	For each variable of interest, give sources of data and details of methods of assessment (measurement). Describe comparability of assessment methods if there is more than one group	6-7	In the CASI, participants who reported at least one sexual partner in the past year (hereon 'sexually active participants') were asked: *'In the last year, have you experienced any of the following for a period of 3 months or longer?'* and were given a list of difficulties and asked to indicate which they had experienced. The list included *'Lacked interest in having sex'*. Those indicating this difficulty were defined as lacking interest in having sex for a period of

For peer review only - http://bmjopen.bmj.com/site/about/guidelines.xhtml

			three months or more in the past year (the outcome for this analysis). Individuals reporting lacking interest in sex for at least 3 months were then asked *'And how do you feel about this?'* with response options: *not at all distressed, a little distressed, fairly distressed; very distressed*. Those answering a little, fairly or very distressed were defined as lacking interest in sex and having distress about this symptom (outcome for sensitivity analysis, see below).
Bias	9	Describe any efforts to address potential sources of bias	-
Study size	10	Explain how the study size was arrived at	-

Continued on next page

For peer review only

Quantitative variables	11	Explain how quantitative variables were handled in the analyses. If applicable, describe which groupings were chosen and why	7	All analyses were done using the complex survey functions of STATA (version 14; StataCorp LP, College Station, Texas) to account for the weighting, clustering, and stratification of the data.
Statistical methods	12	(*a*) Describe all statistical methods, including those used to control for confounding	7	We used multivariable logistic regression to calculate age-adjusted odds ratios (AOR) to examine the associations between reports of lacking interest in sex lasting three months or longer in the past year, and sociodemographic, health, relationship, sexual behaviour, and sexual attitude variables. For each variable, we also tested the interaction between gender, to see if the magnitude of the associations between the above factors and reports of lacking interest in sex was the same for men and women.
		(*b*) Describe any methods used to examine subgroups and interactions	7	-
		(*c*) Explain how missing data were addressed	-	-
		(*d*) *Cohort study*—If applicable, explain how loss to follow-up was addressed<br>*Case-control study*—If applicable, explain how matching of cases and controls was addressed<br>*Cross-sectional study*—If applicable, describe analytical methods taking account of sampling strategy		
		(*e*) Describe any sensitivity analyses	7	We conducted a sensitivity analysis for the outcome variable reporting lack of interest in sex lasting three months or longer *and* distress about

For peer review only - http://bmjopen.bmj.com/site/about/guidelines.xhtml

				this symptom to assess whether similar associations were found.
**Results**				
Participants	13*	(a) Report numbers of individuals at each stage of study—eg numbers potentially eligible, examined for eligibility, confirmed eligible, included in the study, completing follow-up, and analysed	6	For the current analyses, only respondents who reported >=1 sexual partner (opposite-sex or same-sex) in the past year were included (4,839 men and 6,669 women).
		(b) Give reasons for non-participation at each stage	-	
		(c) Consider use of a flow diagram	-	
Descriptive data	14*	(a) Give characteristics of study participants (eg demographic, clinical, social) and information on exposures and potential confounders	6	More extensive details of the survey methodology and sample characteristics are published elsewhere (21,22) and for demographic characteristics of the sample, see (22).
		(b) Indicate number of participants with missing data for each variable of interest	-	
		(c) *Cohort study*—Summarise follow-up time (eg, average and total amount)		
Outcome data	15*	*Cohort study*—Report numbers of outcome events or summary measures over time		
		Case-control study—Report numbers in each exposure category, or summary measures of exposure		
		Cross-sectional study—Report numbers of outcome events or summary measures	Tables 1-2	
Main results	16	(*a*) Give unadjusted estimates and, if applicable, confounder-adjusted estimates and their precision (eg, 95% confidence interval). Make clear which confounders were adjusted for and why they were included	Tables 1-2	
		(*b*) Report category boundaries when continuous variables were categorized	-	
		(*c*) If relevant, consider translating estimates of relative risk into absolute risk for a meaningful time period	-	

Continued on next page

For peer review only - http://bmjopen.bmj.com/site/about/guidelines.xhtml

Other analyses	17	Report other analyses done—eg analyses of subgroups and interactions, and sensitivity analyses	11	Table 2 presents the associations between lacking interest in sex and being distressed about this (as a measure/marker of severity), and the above sociodemographic, health, and sexual relationship/behaviour variables.
**Discussion**				
Key results	18	Summarise key results with reference to study objectives	11-12	We identified a broad range of factors, including some that have not been explored in previous large-scale surveys, that were associated with men's and women's reports of lacking interest in sex in a representative British population-based survey. Our findings, discussed below, revealed some gender similarities as well as some interesting gender differences. The strongest evidence for gender differences was for the relationship context variables, where associations with lacking interest in sex were much stronger for women than for men.
Limitations	19	Discuss limitations of the study, taking into account sources of potential bias or imprecision. Discuss both direction and magnitude of any potential bias	15	Limitations include the cross-sectional nature of the data, which mean that we are unable to infer temporality and causality. We only used a single item to assess lacking

For peer review only - http://bmjopen.bmj.com/site/about/guidelines.xhtml

				interest in sex, although we additionally took account of whether those who reported this also reported that it caused them distress, as a way of trying to capture more problematic lack of interest. It is important to acknowledge, however, that these data do not necessarily correspond to clinical diagnoses. This sensitivity analysis enabled us to demonstrate that for most variables, similar associations exist regardless of whether or not distress was reported. Finally, we have tested many associations within this study and some will have been significant by chance.
Interpretation	20	Give a cautious overall interpretation of results considering objectives, limitations, multiplicity of analyses, results from similar studies, and other relevant evidence	16-17	The findings indicate that reporting lack of interest in sex is associated with a broad range of predictors across sociodemographic, relationship, sexual behaviour, and sexual attitudinal domains.
Generalisability	21	Discuss the generalisability (external validity) of the study results	15	Strengths of our study include the use of national probability sample survey data involving both men and women across a wide age range (21, 22).
**Other information**				
Funding	22	Give the source of funding and the role of the funders for the present study and, if applicable, for the	18	Natsal-3 was supported by grants

For peer review only - http://bmjopen.bmj.com/site/about/guidelines.xhtml

original study on which the present article is based	from the U.K. Medical Research Council (G0701757) and the Wellcome Trust (084840), with support from the Economic and Social Research Council and the Department of Health.

*Give information separately for cases and controls in case-control studies and, if applicable, for exposed and unexposed groups in cohort and cross-sectional studies.

Note: An Explanation and Elaboration article discusses each checklist item and gives methodological background and published examples of transparent reporting. The STROBE checklist is best used in conjunction with this article (freely available on the Web sites of PLoS Medicine at http://www.plosmedicine.org/, Annals of Internal Medicine at http://www.annals.org/, and Epidemiology at http://www.epidem.com/). Information on the STROBE Initiative is available at www.strobe-statement.org.

For peer review only

What factors are associated with reporting lacking interest in sex and how do these vary by gender?: Findings from the third British National Survey of Sexual Attitudes and Lifestyles

Journal:	*BMJ Open*
Manuscript ID	bmjopen-2017-016942.R2
Article Type:	Research
Date Submitted by the Author:	19-Jul-2017

Complete List of Authors:	Graham, Cynthia; University of Southampton, Psychology<br>Mercer, Catherine; University College London, Research Department of Infection and Population Health<br>Tanton, Clare; University College London, Research Department of Infection & Population Health<br>Jones, Kyle; University College London, Research Department of Infection and Population Health<br>Johnson, Anne; University College London, Research Department of Infection and Population Health<br>Wellings, Kaye; London School of Hygiene and Tropical Medicine,<br>Mitchell, Kirstin; London School of Hygiene and Tropical Medicine

**Primary Subject Heading**:	Sexual health
Secondary Subject Heading:	Epidemiology
Keywords:	SEXUAL MEDICINE, EPIDEMIOLOGY, Sexual and gender disorders < PSYCHIATRY

For peer review only

What factors are associated with reporting lacking interest in sex and how do these vary by gender?: Findings from the third British National Survey of Sexual Attitudes and Lifestyles

Graham, C. A.,[1] Mercer, C. H.,[2] Tanton, C.[2] Jones, K. G.,[2] Johnson, A. M.,[2] Wellings, K.,[3] Mitchell, K. R.[4]

[1]Centre for Sexual Health Research, Department of Psychology, University of Southampton, Southampton, U.K.
[2]Centre for Sexual Health and HIV Research, Research Department of Infection & Population Health, University College London, London, U.K.
[3]Centre for Sexual and Reproductive Health Research, Department of Social and Environmental Health Research, London School of Hygiene and Tropical Medicine, London, U.K.
[4]MRC/CSO Social and Public Health Sciences Unit, University of Glasgow, 200 Renfield Street, Glasgow G2 3QB, U.K.

Corresponding author:
Cynthia A. Graham
Department of Psychology
Faculty of Social, Human, and Mathematical Sciences
Shackleton Building (B44)
University of Southampton
Highfield, Southampton
SO17 1BJ UK
tel: 023 8059 3091
e-mail: C.A.Graham@soton.ac.uk

Word count: 3,900

For peer review only

ABSTRACT

Objectives: To investigate factors associated with reporting lacking interest in sex, and how these vary by gender.

Setting: British general population.

Design: Complex survey analyses of data collected for a cross-sectional probability sample survey, undertaken 2010-12, specifically logistic regression to calculate age-adjusted odds ratios (AOR) to identify associated factors.

Participants: 4,839 men and 6,669 women aged 16-74 years who reported >=1 sexual partner (opposite-sex or same-sex) in the past year for the third National Survey of Sexual Attitudes and Lifestyles [Natsal-3].

Main outcome measure: Lacking interest in sex for >=3 months in the past year.

Results: Overall, 15.0% (13.9-16.2) of men and 34.2% (32.8-35.5) of women reported lacking interest in sex. This was associated with age and physical and mental health for both men and women, including self-reported general health and current depression. Lacking interest in sex was more prevalent among men and women reporting STI diagnoses (ever), non-volitional sex (ever), and holding sexual attitudes related to normative expectations about sex. Some gender similarities in associated relationship and family-related factors were evident, including partner having had sexual difficulties in the last year (M: AOR:1.41 [1.07-1.86]; W: AOR:1.60 [1.32-1.94]), not feeling emotionally close to partner during sex (M: 3.74 [1.76-7.93]; W: 4.80 [2.99-7.69], and ease of talking about sex (M: 1.53 [1.23-1.90] W: 2.06 [1.77-2.39]). Among women only, lack of interest in sex was higher among those in a relationship of >1 year in duration and those not sharing the same level of interest (4.57 [3.87-5.38]) or preferences (2.91 [2.22-3.83]) with a partner.

Conclusions: Both gender similarities and differences were found in factors associated with lacking interest in sex, with the most marked differences in relation to some relationship variables. Findings highlight the need to assess, and if appropriate, treat lacking interest in sex in a holistic and relationship-specific way.

For peer review only

ARTICLE SUMMARY

Strengths and limitations of this study

- This study used nationally representative data to investigate factors associated with reporting lacking interest in sex, and how these vary by gender, in the British population.
- Few previous population-based studies have obtained data on low sexual interest from men and women and made direct comparisons between them.
- The study included detailed assessment of a range of relationship context and attitudinal variables seldom included in previous population-based surveys.
- Information about lacking interest in sex was assessed with a single item, asking participants whether they had lacked interest in having sex for a period of three months or more in the past year. Those who reported this were also asked whether they experienced associated distress.
- The cross-sectional data do not allow us to establish the causal direction of associations between lacking interest in sex and variables of interest.

For peer review only

In Britain's third National Survey of Sexual Attitudes and Lifestyles (Natsal-3) lacking interest in sex was the most common sexual difficulty reported by both men and women (1). Lacking interest in sex for 3 months or more in the past year was twice as common in women compared to men. When duration and symptom severity criteria are considered (i.e., that symptoms last six months or more and occur "very often" or "always") these prevalence estimates are much lower (2), but the gender difference is maintained.

Researchers have paid more attention to problems of low sexual interest in women than in men (3-5). Among men the predominant focus has been on erectile functioning and on physiological causes of lacking interest in sex such as hormonal status, rather than on psychosocial determinants. This lack of attention to male problems is reflected in recent revisions to the Diagnostic and Statistical Manual (DSM-5) classification of sexual disorders (6) which involved major changes to sexual arousal and desire disorder categories in women, but no substantive changes for male disorders.

Most but not all studies involving men have reported an association between low sexual interest and increasing age (for review, see 7). However, there are conflicting findings on the association with physical and mental health (8,9). Limited research suggests that psychosocial and relationship factors may also be associated with low sexual desire in men (8, 10-12).

Among women, factors that have been consistently associated with lacking interest in sex are relationship problems, relationship quality, and partner's sexual functioning (13-17), poor physical health (18), and negative mood states/depression (13, 18,19). There are inconsistent findings on the association between low sexual

For peer review only - http://bmjopen.bmj.com/site/about/guidelines.xhtml

interest and both age and menopausal status (14,18). Few large-scale surveys have examined possible links between lacking interest in sex and either sexual attitudes or sexual behaviour. In the second wave of the British National Survey of Sexual Attitudes and Lifestyles (Natsal-2), among women, lacking interest in sex was associated with lower frequency of sex and attitudes according sex low priority (20).

Studies have, for the most part, used small, clinical samples of patients seeking treatment for low sexual desire problems. The potential for bias in such studies is revealed in previously reported findings from Natsal-3 that only around a third of men and women with one or more sexual function problems meeting DSM 5 morbidity criteria had sought professional help in the last year. (2). The few large-scale probability-based surveys involving both men and women have focused on associations between low sexual desire and sociodemographic factors.

In summary, the evidence on the factors associated with men's and women's reports of low sexual desire is drawn largely from non-representative samples, is somewhat equivocal and, in men, sparse. Given that most previous research has involved non-representative samples, it is important to explore how correlates might differ in a population-based sample. Understanding the correlates of lacking interest in sex is key to informing therapeutic options for this group.

The research questions addressed in this paper are: (1) What sociodemographic, relationship, sexual behaviour, and sexual attitudinal factors are associated with lacking interest in sex in sexually active men and women?; (2) To what extent do these factors vary by gender?; (3) To what extent does lacking interest in sex co-exist with other sexual function problems?

METHOD

For peer review only

Participants and Procedure

Natsal-3 is a probability sample survey of 15,162 men and women aged 16-74 years in Britain, interviewed between September 2010 and August 2012. A multi-stage, clustered, and stratified probability sample design was used and participants were interviewed in their homes by professional interviewers using a combination of computer-assisted personal interviews (CAPI), and computer-assisted self-interviews (CASI) for the more sensitive questions (including, of relevance to this paper, those on sexual function), Interviewers were present in the room while participants completed the CASI, but did not view responses (20). After weighting to adjust for unequal probabilities of selection and to match the British population in terms of age, gender and geographical region, the Natsal-3 sample was broadly representative, on key variables, of the British population as described by the 2011 Census (21).

The estimated response rate was 57.7%, and the estimated cooperation rate (the number of interviews completed from eligible addresses for which contact was made) was 65.8% (of all eligible addressed contacted) (22). More extensive details of the survey methodology and sample characteristics are published elsewhere (21,22) and for demographic characteristics of the sample, see (22). Participants provided oral informed consent for interviews and the survey was approved by the NRES Committee South-Central – Oxford A (Ref.: 10/H0604/27).

Only respondents who reported >=1 sexual partner (opposite-sex or same-sex) in the past year (4,839 men and 6,669 women) were asked whether they had lacked interest in sex for a period of three month or longer in the past year (see below). These participants were the focus of the current analyses.

Outcome Measures

For peer review only

Items were drawn from the Natsal-SF, a measure of sexual function, designed and validated for population surveys. The measure comprises items on problems with sexual response, relational aspects of sexual function, and self-appraisal of sex life (23,24). Participants who reported at least one sexual partner in the past year (hereon 'sexually active participants') were asked: *'In the last year, have you experienced any of the following for a period of 3 months or longer?'* and were given a list of difficulties and asked to indicate which they had experienced. The list included *'Lacked interest in having sex'*. Those indicating this difficulty were defined as lacking interest in having sex for a period of three months or more in the past year (the outcome for this analysis). Individuals reporting lacking interest in sex for at least 3 months were then asked *'And how do you feel about this?'* with response options: *not at all distressed, a little distressed, fairly distressed; very distressed*. Those answering a little, fairly or very distressed were defined as lacking interest in sex and having distress about this symptom (outcome for sensitivity analysis, see below).

Statistical Analysis

All analyses were done using the complex survey functions of STATA (version 14; StataCorp LP, College Station, Texas) to account for the weighting, clustering, and stratification of the data. We used multivariable logistic regression to calculate age-adjusted odds ratios (AOR) to examine the associations between reports of lacking interest in sex lasting three months or longer in the past year, and sociodemographic, health, relationship, sexual behaviour, and sexual attitude variables. For each variable, we also tested the interaction with gender, to see if the magnitude of the associations between the above factors and reports of lacking interest in sex was the same for men and women. We conducted a sensitivity analysis for the outcome variable reporting

For peer review only

lack of interest in sex lasting three months or longer *and* distress about this symptom to assess whether similar associations were found. We also examined the association between reporting lacking interest in sex and the other sexual function problems asked about in Natsal-3, using AORs.

RESULTS

Overall, 15.0 (95% CI 13.9-16.2) of sexually active men and 34.2% (95% CI 32.8-35.5) of sexually active women reported lacking interest in sex for three months or longer in the year prior to interview. Table 1 presents the associations between lacking interest in sex and sociodemographic, health, relationship, sexual behaviour, and sexual attitudinal variables for men and women.

Age was significantly associated with lacking interest in sex. Prevalence increased with age, being lowest among younger participants (16-24 years; M: 11.5%; W: 24.8%) and peaking in men aged 35-44 years (17.2%) and in women aged 55-64 years (38.8%). Regarding demographic variables, after adjusting for age, lack of interest was associated with leaving school at 16 (men only; AORS: 1.31), being unemployed (men only AORs: M: 1.44), and less frequent religious practice (women only; AOR: 0.79). In women, after adjusting for age, those who were students or retired were less likely to lack desire.

After adjusting for age, there were associations between all physical and mental health variables assessed and lacking interest in sex. Individuals in poorer health (AORs: M: 3.29; W: 1.93), those who had much difficulty walking upstairs (AORs: M: 2.68; W: 1.55), those with a longstanding medical condition (AORs: M 1.76; W: 1.35), and those who had screened positive for current depression (AORs: M: 2.95; W: 2.79) or who had been treated for depression in the past year (AORs: M: 2.82; W:

For peer review only

2.32) were more likely to report lacking interest in sex. The magnitude of these associations was similar for men and women. A greater number of comorbid health conditions was significantly associated with lacking interest in sex among both men and women. Menopausal status in women and circumcision in men were not associated with the likelihood of lacking sexual interest.

Regarding sexual behaviour, among both men and women, lack of interest was associated with frequency of sexual activity (defined as vaginal, oral or anal intercourse) in the four weeks prior to interview; 12.4% of men, and 33.8% of women who reported having engaged in 3-4 sexual acts reported lack of interest, vs. 20.7% of men and 42.9% of women who reported no sexual activity. Associations with recent masturbation differed by gender; lack of interest in sex was slightly *more* common among men who reported having recently masturbated but *less* common among women who did so. Women with three or more partners in the past year were less likely to report low sexual interest than those with only one partner (AOR: 0.70) but there was no association between partner numbers and lacking interest in sex in men. Among men only, those who reported ever having taken drugs to assist sexual performance were more likely to report lacking interest in sex (AOR: 1.36). A similar magnitude association was seen for women (AOR: 1.39) however, fewer women reported ever having taken drugs and the 95%CI therefore crosses 1.

Associations were found between lacking interest in sex and several relationship contextual variables and for many of these variables associations were stronger for women than for men. For both men and women, lack of interest was associated with relationship status; women living with a partner were more likely to lack interest in sex than those in other relationship categories (see Table 1). For

For peer review only

women, all relationship categories had lower AORs than living with partner. Duration of most recent sexual relationship was significantly associated with lacking interest in sex only among women, being more common among those in longer relationships.

Among both men and women, there was an association between ease of communication and lacking interest in sex. Those who found it "always easy to talk about sex" with their partner were less likely to report low interest. Lack of interest was more likely among those whose partner had sexual difficulties in the last year, and those who reported a lower assessment of happiness with the relationship, and not feeling emotionally close to partner during sex. Among women but not men, not sharing the same level of sexual interest with a partner, and not sharing the same sexual likes and dislikes, were also associated.

Having been pregnant in the last year was associated with lacking sexual interest as was having one or more young child(ren) (women only). Lack of interest in sex was significantly associated with sexual health indicators, including previous STI diagnosis and ever having experienced non-volitional sex. The strength and direction of associations was similar for men and women, except for reporting another sexual function problem, which was significant for two or more problems in men, but one or more problems in women. Sexual competence at first sex was significantly associated with lack of interest in sex only among women.

Regarding attitudinal variables, both men and women who endorsed statements that "people are under pressure to have sex" and "people want less sex as they age" were more likely to report lacking interest in sex over the past year. The only attitudinal variable that showed a significant interaction with gender was that which related to men having a "naturally higher sex drive than women." Men who

For peer review only

agreed with this statement were *less* likely than those who disagreed to lack interest in sex, while the reverse was true among women.

Table 2 presents the associations between lacking interest in sex and being distressed about this (as a measure/marker of severity), and the above sociodemographic, health, and sexual relationship/behaviour variables. While prevalence was lower, the associations and the interactions with gender were generally similar; however, some of the previous gender-specific associations with variables (e.g., masturbation, and pregnancy in women, and education in men) were no longer significant when the outcome variable was reported low sexual interest *and* associated distress. In addition, some associations became stronger when considering only those who reported a distressing lack of interest in sex (vs. lack of interest without any reported distress). For example, the association between depressive symptoms and having been treated for depression in the past year, was stronger in men than in women.

Regarding the association between reporting lacking interest in sex and the other sexual function problems asked about in Natsal-3, the strongest (positive) associations were for lacking enjoyment in sex (AORs=9.78 and 8.95 for men and women, respectively), followed by feeling no excitement or arousal during sex (AORs=9.21 and 9.16 for men and women, respectively) (see Table 3).

DISCUSSION

We identified a broad range of factors, including some that have not been explored in previous large-scale surveys, that were associated with men's and women's reports of lacking interest in sex in a representative British population-based survey. Our findings, discussed below, revealed some gender similarities as well as

For peer review only

some interesting gender differences. The strongest evidence for gender differences was for the relationship context variables, where associations with lacking interest in sex were much stronger for women than for men.

Interpretation of findings in context of previous research

Our finding relating to differences by age is consistent with some, but not all, results from previous research which has yielded generally inconsistent findings. Some studies have, like ours, shown a higher prevalence of sexual interest problems in older than in younger women (25-27). Others have found no association between age and low sexual interest complaints (14, 28) and yet more have shown lack of sexual interest to be more common among younger women (18). Whereas we found a marginal relationship with age in men, some studies (though not all e.g., 29) have found a stronger relationship (12, 30). It is possible that the varied findings might in part be a result of varied definitions of low sexual interest or differences in sampling.

The finding in this analysis that having young children appears to increase the likelihood of reporting lack of sexual interest for women, but not for men, remains unchanged since the previous Natsal-2 survey (31). This may be due to fatigue associated with a primary caring role (32), the fact that daily stress appears to affect sexual functioning in women more than men (33), or possibly a shift in focus of attention attendant on bringing up small children.

The finding of a link between lacking interest in sex and lacking enjoyment in sex and/or feeling no excitement or arousal during sex is not surprising and has been shown in previous studies (3). The strong associations between lack of interest in sex and physical and mental health indicators, which we observed for both men and women, is not entirely consistent with findings from other studies. While this link has

For peer review only

been persuasively shown for women (13, 18, 19), in men, the evidence is more equivocal. In a study of men attending an outpatient clinic for sexual problems, psychological symptoms such as anxiety and depression were more predictive of low sexual desire than hormonal or other physical markers (11). In contrast, DeRogatis et al. (9), in their study of men with erectile dysfunction, observed no differences in depressive symptoms, concurrent illness, or medication use between men with and without symptoms of low sexual desire.

The gender differences in associations between masturbation and a lack of sexual interest are interesting and have been explored in few previous population-based studies. Our observation that lack of interest was *more* commonly reported by men who had recently masturbated, but *less* commonly reported by women who had done so may reflect a tendency among women for self-pleasuring to be, not a substitute for partnered sex but instead a part of a broader repertoire of sexual fulfilment; this possibility is worthy of further exploration. In contrast, for men frequency of masturbation reflects reduced frequency of partnered sex (34). However, it is worth noting that in the U.S. National Health and Social Life Survey, lifetime number of sexual partners and masturbation practices were unrelated to the likelihood of sexual desire difficulties for either men or women (35).

Our observation that duration of most recent sexual relationship showed a strong association with lacking interest in sex in women is consistent with previous studies (15, 17). There has been little comparable research on men with which to corroborate the absence of such an association among men in our analysis.

Our data confirm the importance of the relational context in individuals' level of sexual interest. The strong associations between relationship and partner factors

For peer review only

and sexual interest are consistent with those shown in many previous studies relating to women (13-17) and with a much smaller literature in men (36,37). In particular, sexual dysfunction in a male partner has previously been associated with women's levels of sexual desire (15, 38, 39), and sexual desire discrepancy in couples has been linked to lower reported relationship satisfaction and more couple conflict (40).

The strong links found between several key sexual health outcomes and lack of interest in sex are interesting; among both men and women, reporting an STI diagnosis and non-volitional sex were associated with reporting lack of interest in sex. Our finding that lacking "sexual competence" at first sexual intercourse was linked with subsequent lack of interest in sex among women but not men, may reflect a greater salience of contextual aspects of first sex for women. More women than men report being pressured by a partner on the first occasion of heterosexual intercourse, and to have subsequently experienced regret about first sexual experiences (41). These findings suggest that for women, early sexual experiences may shape future sexual encounters/relationships to a greater extent than for men.

To our knowledge no previous studies have assessed the association between attitudes toward sexual matters and lack of interest in sex. Endorsing the assumption that "people want less sex as they age" was associated with lack of interest in both genders. It might be that this belief contributes to a decline in interest, or – equally plausible – that those who lack interest adopt this attitude to avoid viewing their experience as problematic. Interestingly, men who endorsed the view that "men have a higher sex drive than women" were significantly *less* likely to report lacking interest in sex, whereas women who agreed with this statement were *more* likely to do so. If people responded to this statement with reference to their own relationship, these

For peer review only

findings may be seen as making intuitive sense. The results suggest that endorsing stereotypical gender-norms related to sex may adversely affect women more than men.

Strengths and Limitations

Strengths of our study include the use of national probability sample survey data involving both men and women across a wide age range (21, 22). With a few exceptions (e.g., 12, 14, 29, 42), most surveys on sexual desire problems have sampled either men *or* women, precluding direct comparisons within the same sample. Another strength was the detailed and holistic examination of relationship context and attitudinal variables, which few previous studies have reported. Response rates for Natsal-3 were also similar to those of other major social surveys in Britain (43) and higher than many previous surveys of sexual problems (35, 44).

Limitations include the cross-sectional nature of the data, which mean that we are unable to infer temporality and causality. The sample is representative of those resident in private households in Britain i.e., not those living in institutions. We included only respondents who reported >=1 sexual partner (opposite-sex or same-sex) in the past year, excluding those who had not had sex because of lack of interest. We only used a single item to assess lacking interest in sex, although we additionally took account of whether those who reported this also reported that it caused them distress, as a way of trying to capture more problematic lack of interest. This sensitivity analysis enabled us to demonstrate that for most variables, similar associations exist regardless of whether or not distress was reported. It is important to acknowledge, however, that these data do not necessarily correspond to clinical diagnoses. Finally, we have tested many associations within this study and some will

For peer review only

have been significant by chance. These were exploratory and descriptive analyses of zero-order relationships and therefore some of the smaller effect sizes may not replicate and may not hold in multivariable analyses.

Implications for Research and Practice

The findings indicate that lack of interest in sex is associated with a broad range of factors across sociodemographic, relationship, sexual behaviour, and sexual attitudinal domains. There are both research and clinical applications of our results.

Firstly, our findings underscore the importance of the relational context in understanding low sexual interest in both men and women. For women in particular, the experience of sexual interest appears strongly linked with their perceptions of the quality of their relationships, their communication with partners, and their expectations/attitudes about sex. Our findings support the view that transient (and often adaptive) reductions in sexual desire are not evidence of "dysfunction" (45). In the context of the recent FDA approval of flibanserin, the first drug to treat low sexual desire in women (46), these findings are relevant to the current debate about whether striving for a pharmaceutical solution to women's sexual desire problems is an appropriate and feasible goal (45, 47). Some authors have suggested that women with complaints of low sexual interest might benefit most from integrative approaches that accord with a biopsychosocial model (48).

Secondly, our findings on the strong association between open sexual communication (i.e., "finding it always easy to talk about sex") and a reduced likelihood of reporting lack of interest in sex, particularly for women, emphasise the importance of providing a broad sexual and relationships education, rather than limiting attention only to adverse consequences of sex and how to prevent them.

For peer review only - http://bmjopen.bmj.com/site/about/guidelines.xhtml

Similarly, the important role of early sexual experiences, and sexual "competence," especially for women, in shaping later experiences of sexual desire supports the need for comprehensive sex education.

In a clinical context, our findings emphasise the importance of health care professionals assessing psychological and interpersonal variables in individuals presenting with complaints of low sexual interest (49). In couple therapy, it is important that therapists have an awareness of the differences between men and women in the factors associated with low sexual interest. For example, among the subgroup of participants reporting both lack of interest in sex and related distress, we found a stronger association between depressive symptoms and treatment for depression in the last year among men compared with women. Lastly, our findings support previous research on the critical role of physical and mental health in understanding low sexual interest problems experienced by men and women (11, 18).

Conclusions

This study extends our understanding of the factors associated with lack of interest in sex in men and women, the gender similarities and differences, and highlights the need to assess and – if necessary – treat sexual desire problems in a holistic and relationship- as well as gender-specific way.

For peer review only

Contribution to authorship

The paper was conceived by CAG, CHM, AMJ, KW, and KRM. CAG wrote the first draft, with further contributions from all authors. Statistical analyses were undertaken by CHM, CT, and KGJ. CHM, AMJ (Principal Investigator) and KW, initial applicants on Natsal-3, wrote the study protocol and obtained funding. Natsal-3 questionnaire design, ethics applications, and piloting were undertaken by CHM, CT, AMJ, KW, and KRM. Data management was undertaken by NatCen Social Research, UCL and LSHTM. All authors contributed to data interpretation, reviewed successive drafts and approved the final version of the manuscript.

Competing interests statement

AMJ has been a Governor of the Wellcome Trust since 2011. The remaining authors have nothing to disclose. Completed disclosure of interests form available to view online as supporting information.

Funding Statement

Natsal-3 was supported by grants from the U.K. Medical Research Council (G0701757) and the Wellcome Trust (084840), with support from the Economic and Social Research Council and the Department of Health. Since September 2015, Kirstin Mitchell has been supported by the United Kingdom Medical Research Council grant MC_UU_12017/11, and Scottish Government Chief Scientist Office grant SPHSU11.

Details of ethics approval

Natsal-3 was approved by the NRES Committee South Central-Oxford A (Ref: 10/H0604/27) on 12 July 2010. Participants provided oral informed consent for interviews.

Data sharing statement

The Natsal-3 dataset is publicly available from the UK Data Service: https://discover.ukdataservice.ac.uk/; SN: 7799; persistent identifier: 10.5255/UKDA-SN-77991-1.

For peer review only

For peer review only

REFERENCES

1. Mitchell KR, Mercer CH, Ploubidis GB, et al. Sexual function in Britain: Findings from the third National Survey of Sexual Attitudes and Lifestyles (Natsal-3). *Lancet* 2013;382:1817-1829.

2. Mitchell KR, Jones KG, Wellings K, et al. Estimating the prevalence of sexual function problems: The impact of morbidity criteria. *J Sex Res* 2016;53:55-967.

3. Brotto LA. The DSM diagnostic criteria for hypoactive sexual desire disorder in women. *Arch Sex Behav* 2010a; 39:221-239.

4. Carvalho J, Nobre P. Biopsychosocial determinants of men's sexual desire: testing an integrative model. *J Sex Med* 2011;8:754-763.

5. Štulhofer A, Carvalheira AA, Træen B. Is responsive sexual desire for partnered sex problematic among men? Insights from a two-country study. *Sex Relation Ther* 2013;28:246-258.

6. American Psychiatric Association. *Diagnostic and statistical manual of mental disorders*, 5th. ed. Arlington, VA: Author 2013.

7. Brotto LA. The DSM diagnostic criteria for hypoactive sexual desire disorder in men. *J Sex Med* 2010b;7:2015-2030.

8. Corona G, Lee DM, Forti G, et al. Age-related changes in general and sexual health in middle-aged and older men: Results from the European Male Ageing Study (EMAS). *J Sex Med* 2010;7:1362-1380.

9. DeRogatis L, Rosen RC, Goldstein I, et al. Characterization of hypoactive sexual desire disorder (HSDD) in men. *J Sex Med* 2012;9:812-820.

10. Carvalheira A, Træen B, Štulhofer A. Correlates of men's sexual interest: a cross-cultural study. *J Sex Med* 2014;11:154-164.

11. Corona G, Petrone L, Mannucci E, et al. The impotent couple: low desire. *Int J Androl* 2005;28:46-52.

12. DeLamater JD, Sill M. Sexual desire in later life. *J Sex Res* 2005;42:138-149.

13. Dennerstein L, Koochaki P, Barton I, et al. Hypoactive sexual desire disorder in menopausal women: a survey of Western European women. *J Sex Med* 2006;3:212-222.

14. Hayes RD, Dennerstein L, Bennett CM, et al. Risk factors for female sexual dysfunction in the general population: Exploring factors associated with low sexual function and sexual distress. *J Sex Med* 2008;5:1681-1693.

15. McCabe MP, Goldhammer DL. Demographic and psychological factors related to sexual desire among heterosexual women in a relationship. *J Sex Res* 2012;49:78-87.

16. Öberg K, Sjögren Fugl-Meyer K. On Swedish women's distressing sexual dysfunctions: some concomitant conditions and life satisfaction. *J Sex Med* 2005;2:169-180.

17. Witting K, Santtila P, Varjonen M, et al. Female sexual dysfunction, sexual distress, and compatibility with partner. *J Sex Med* 2008;5:2587-2599.

18. Shifren JL, Monz BU, Russo PA, et al. Sexual problems and distress in United States women. *Obstet Gynecol* 2008;112:970-978.

19. Johannes CB, Clayton AH, Odom DM, et al. Distressing sexual problems in United States women revisited: prevalence after accounting for depression. *J Clin Psychiatry* 2009;70:1698-1706.

For peer review only

20. Mitchell KR, Mercer CH, Wellings K, Johnson AM. Prevalence of low sexual desire among women in Britain: Associated factors. *J Sex Med*. 2009; 1;6(9):2434-2444.

21. Erens B, Phelps A, Clifton S, et al. Methodology of the third British National Survey of Sexual Attitudes and Lifestyles (Natsal-3). *Sex Transm Infect* 2014;90:84-89.

22. Mercer CH, Tanton C, Prah P, et al. Changes in sexual attitudes and lifestyles in Britain through the life course and over time: findings from the National Surveys of Sexual Attitudes and Lifestyles (Natsal). *Lancet* 2013;382:1781-1794.

23. Jones KG, Mitchell KR, Ploubidis GB, et al. The Natsal-SF measure of sexual function: Comparison of three scoring methods. *J Sex Res* 2015;52:640-646.

24. Mitchell KR, Ploubidis GB, Datta J, et al. The Natsal-SF: a validated measure of sexual function for use in community surveys. *Eur J Epidemiol* 2012;27:409-18.

25. Abdo CHN, Oliveira WM, Moreira ED, et al. Prevalence of sexual dysfunctions and correlated conditions in a sample of Brazilian women—results of the Brazilian study on sexual behavior (BSSB). *Int J Impot Res* 2004;16:160-166.

26. Hayes RD, Dennerstein L, Bennett CM, et al. Relationship between hypoactive sexual desire disorder and aging. *Fertil Steril* 2007;87:107-112.

27. Peixoto MM, Nobre P. Prevalence and sociodemographic predictors of sexual problems in Portugal: A population-based study with women aged 18 to 79 years. *J Sex Marital Ther* 2015;41:169-180.

28. Rosen RC, Shifren JL, Monz BU, et al. Correlates of sexually-related personal distress in women with low sexual desire. *J Sex Med* 2009;6:1549-1560.

29. Laumann EO, Glasser DB, Neves RCS, et al. A population-based survey of sexual activity, sexual problems and associated help-seeking behavior patterns in

For peer review only

mature adults in the United States of America. *Int J Impot Res* 2009;21:171-178.

30. Eplov L, Giraldi A, Davidsen M, et al. Sexual desire in a nationally representative Danish population. *J Sex Med* 2007;4:47-56.

31. Mercer CH, Fenton KA, Johnson AM, et al. Who reports sexual function problems? Empirical evidence from Britain's 2000 National Survey of Sexual Attitudes and Lifestyles. *Sex Transm Infect* 2005;81:394-399.

32. Park A, Bryson C, Clery E, et al. *British social attitudes 30*. London: NatCen 2013.

33. Bodenmann G, Ledermann T, Blattner D, et al. Associations among everyday stress, critical life events, and sexual problems. *J of Nerv Ment Dis* 2006;194:494-501.

34. Gerressu M, Mercer CH, Graham CA, et al. Prevalence of masturbation of associated factors in a British national probability survey. *Arch Sex Behav* 2008;37:266-278.

35. Laumann EO, Paik A, Rosen RC. Sexual dysfunctions in the United States: Prevalence and predictors. *JAMA* 1999;281:537-544.

36. Janssen E, McBride KR, Yarber W, et al. Factors that influence sexual arousal in men: A focus group study. *Arch Sex Behav* 2008;37:252-265.

37. Murray S, Milhausen RR, Graham CA, et al. A qualitative exploration of factors that affect sexual desire among men aged 30 to 65 in long-term relationships. *J Sex Res* 2016;doi 10.1080/00224499.2016.1168352

38. Çayan S, Bozlu M, Canpolat B, et al. The assessment of sexual functions in women with male partners complaining of erectile dysfunction: does treatment of

For peer review only

male sexual dysfunction improve female partner's sexual functions? *J Sex Marital Ther* 2004;30:333-341.

39. Fisher WA, Rosen RC, Eardley I, et al. Experience of female partners of men with erectile dysfunction: The female experience of men's attitudes to life events and sexuality (FEMALES) study. *J Sex Med* 2005;2:675-668.

40. Willoughby BJ, Farero AM, Busby DM. Exploring the effects of sexual desire discrepancy among married couples. *Arch Sex Behav* 2014;43:551-562.

41. Hawes ZC, Wellings K, Stephenson J. First heterosexual intercourse in the United Kingdom: A review of the literature. *J Sex Res* 2010;47:137-152.

42. Laumann EO, Nicolosi A, Glasser DB, et al. Sexual problems among women and men aged 40–80 years: Prevalence and correlates identified in the Global Study of Sexual Attitudes and Behaviors. *Int J Impot Res* 2005;17:39-57.

43. Park A, Clery E, Curtice J, et al. *British social attitudes: The 28th report*. London, England: NatCen 2012.

44. Christensen BS, Grønbæk M, Osler M, et al. Sexual dysfunctions and difficulties in Denmark: Prevalence and associated sociodemographic factors. *Arch Sex Behav* 2011;40:121-132.

45. Graham CA, Boynton PM, Gould K. Women's sexual desire: Challenging narratives of dysfunction. *Eur Psychol*, in press.

46. Woloshin S, Schwartz LM. US Food and Drug Administration approval of flibanserin: Even the Score does not add up. *JAMA Intern Med* 2016; doi: 10.1001/jamainternmed.2016.0073

47. Hart G, Wellings K. Sexual behaviour and its medicalisation: In sickness and in health. BMJ 2002;324:896-900.

For peer review only

48. Frühauf S, Gerger H, Schmidt HM, et al. Efficacy of psychological interventions for sexual dysfunction: a systematic review and meta-analysis. *Arch Sex Behav* 2013;42:915-933.

49. Brotto LA, Atallah CS, Johnson-Agbakwu C, et al. Psychological and interpersonal dimensions of sexual function and dysfunction. *J Sex Med* 2016;13:538-571.

50. Payne RA, Abel GA. UK indices of multiple deprivation-a way to make comparisons across constituent countries easier. *Health Stat Q* 2012;53:22-37.

For peer review only

Table 1: Factors associated with lacking interest in having sex for at least 3 months in the past year in sexually active men and women

	Men						Women						
	Denom. (unwt, wt)	%	(95%CI)	Age-adjusted OR	(95%CI)	p-value	*Denom. (unwt, wt)*	%	(95%CI)	Age-adjusted OR	(95%CI)	p-value	*p-value for interaction with sex*[a]
**All**	*4839, 5973*	**15.0%**	**(13.9-16.2)**				*6669, 5755*	**34.2%**	**(32.8-35.5)**				
**Socio-demographics**													
**Age group**						0.0471						<0.0001	*0.6733*
16-24	*1279, 936*	11.5%	(9.4-14.0)	1	-		*1662, 923*	24.8%	(22.5-27.1)	1	-		
25-34	*1376, 1238*	14.6%	(12.7-16.6)	1.32	(1.00-1.73)		*2236, 1246*	31.9%	(29.8-34.1)	1.42	(1.22-1.66)		
35-44	*719, 1298*	17.2%	(14.5-20.4)	1.61	(1.19-2.18)		*1050, 1290*	36.8%	(33.7-40.1)	1.77	(1.48-2.13)		
45-54	*630, 1186*	15.3%	(12.5-18.7)	1.40	(1.01-1.95)		*871, 1186*	37.9%	(34.5-41.5)	1.86	(1.53-2.25)		
55-64	*512, 849*	16.5%	(13.4-20.2)	1.53	(1.10-2.13)		*569, 755*	38.8%	(34.5-43.2)	1.92	(1.55-2.39)		
65-74	*323, 467*	13.9%	(10.4-18.3)	1.22	(0.81-1.82)		*281, 355*	34.2%	(28.4-40.5)	1.58	(1.18-2.12)		
**Index of Multiple Deprivation (quintiles)**[b]						0.093						0.0316	*0.0111*
1 (least deprived)	*977, 1279*	13.9%	(11.6-16.6)	1	-		*1248, 1208*	35.7%	(32.6-38.9)	1	-		
2	*962, 1264*	13.0%	(10.8-15.6)	0.93	(0.69-1.25)		*1290, 1208*	33.6%	(30.6-36.7)	0.92	(0.76-1.13)		
3	*942, 1169*	18.0%	(15.2-21.2)	1.38	(1.04-1.85)		*1299, 1116*	30.1%	(27.2-33.2)	0.81	(0.66-0.99)		
4	*967, 1184*	15.3%	(12.8-18.3)	1.15	(0.86-1.55)		*1384, 1137*	35.9%	(33.0-39.0)	1.08	(0.89-1.30)		
5 (most deprived)	*991, 1077*	15.1%	(12.7-17.8)	1.14	(0.85-1.52)		*1448, 1086*	35.3%	(32.4-38.3)	1.06	(0.87-1.28)		
**Education level**[c]						0.0083						0.2453	0.2914
Left school aged 17+	*2862, 3464*	13.5%	(12.1-15.1)	1	-		*4150, 3406*	32.7%	(31.0-34.5)	1	-		
Left school at 16	*1873, 2437*	17.2%	(15.3-19.4)	1.31	(1.07-1.60)		*2409, 2287*	36.6%	(34.4-38.9)	1.08	(0.95-1.23)		
**Employment status**						0.0086						0.0003	*0.0766*
Employed	*3211, 4254*	14.7%	(13.3-16.1)	1	-		*3871, 3517*	34.6%	(32.9-36.4)	1	-		
Full-time education	*542, 431*	12.6%	(8.8-17.5)	0.98	(0.64-1.51)		*693, 423*	22.5%	(19.0-26.4)	0.70	(0.55-0.89)		
Unemployed	*707, 723*	19.6%	(16.3-23.4)	1.44	(1.12-1.86)		*1681, 1282*	36.1%	(33.4-39.0)	1.11	(0.96-1.28)		
Retired	*375, 562*	13.6%	(10.4-17.7)	0.75	(0.52-1.09)		*415, 524*	35.8%	(31.0-40.9)	0.75	(0.57-0.99)		
**Practises religion at least once a month**						0.1687						0.0082	*0.9966*
No	*4283, 5179*	15.3%	(14.1-16.6)	1	-		*5659, 4754*	34.8%	(33.3-36.3)	1	-		
Yes	*521, 748*	12.9%	(10.0-16.4)	0.81	(0.60-1.09)		*956, 945*	30.7%	(27.5-34.2)	0.79	(0.67-0.94)		

For peer review only

Table 1 cont.

	Men						Women						
	Denom. (unwt, wt)	%	(95%CI)	Age-adjusted OR	(95%CI)	p-value	Denom. (unwt, wt)	%	(95%CI)	Age-adjusted OR	(95%CI)	p-value	*p-value for interaction with sex*[a]
**Health**													
**Self-reported general health**						<0.0001						<0.0001	*0.1890*
Very good/Good	*4123, 5055*	13.4%	(12.2-14.6)	1	-		*5683, 4851*	32.3%	(30.9-33.8)	1	-		
Fair	*580, 745*	21.9%	(18.3-25.8)	1.8	(1.41-2.30)		*780, 709*	42.2%	(38.2-46.3)	1.45	(1.21-1.75)		
Bad/very bad	*135, 171*	33.9%	(25.3-43.6)	3.29	(2.14-5.06)		*206, 195*	49.9%	(42.2-57.7)	1.93	(1.40-2.67)		
**Difficulty walking up stairs because of a health problem**						<0.0001						0.0497	*0.1179*
No difficulty	*4475, 5460*	14.1%	(12.9-15.3)	1	-		*6062, 5107*	33.3%	(31.8-34.7)	1	-		
Some difficulty	*278, 393*	23.0%	(18.1-28.8)	1.8	(1.30-2.49)		*450, 482*	39.2%	(34.4-44.2)	1.15	(0.92-1.43)		
Much difficulty/unable to do this	*86, 120*	30.9%	(20.9-43.0)	2.68	(1.57-4.57)		*157, 166*	47.0%	(38.0-56.1)	1.55	(1.06-2.25)		
**Longstanding illness or disability**						<0.0001						<0.0001	*0.1348*
No	*3585, 4259*	12.8%	(11.6-14.2)	1	-		*4843, 4026*	31.6%	(30.0-33.2)	1	-		
Yes	*1253, 1713*	20.5%	(18.1-23.1)	1.76	(1.44-2.16)		*1825, 1729*	40.1%	(37.5-42.8)	1.35	(1.17-1.55)		
**Number of comorbid conditions**[d]						<0.0001						<0.0001	*0.7951*
0	*3453, 3994*	12.8%	(11.5-14.1)	1	-		*4357, 3536*	29.9%	(28.2-31.5)	1	-		
1	*939, 1329*	18.9%	(16.2-21.9)	1.64	(1.30-2.06)		*1555, 1416*	38.6%	(35.9-41.5)	1.42	(1.23-1.64)		
>=2	*446, 650*	21.0%	(17.0-25.6)	1.91	(1.41-2.60)		*755, 802*	45.1%	(41.2-49.1)	1.75	(1.45-2.13)		
**Depressive symptoms**[e]						<0.0001						<0.0001	*0.6249*
No	*4383, 5471*	13.5%	(12.4-14.8)	1	-		*5885, 5149*	31.7%	(30.2-33.1)	1	-		
Yes	*449, 495*	31.3%	(26.4-36.7)	2.95	(2.26-3.85)		*780, 602*	55.2%	(51.0-59.5)	2.79	(2.32-3.37)		
**Treated for depression, past year**						<0.0001						<0.0001	*0.2447*
No	*4524, 5630*	14.0%	(12.9-15.2)	1	-		*5770, 5040*	31.7%	(30.2-33.2)	1	-		
Yes	*313, 342*	31.5%	(25.7-38.0)	2.82	(2.08-3.83)		*897, 713*	51.4%	(47.6-55.2)	2.32	(1.96-2.75)		

For peer review only

Table 1 cont.

	Men						Women						
	Denom. (unwt, wt)	%	(95%CI)	Age-adjusted OR	(95%CI)	p-value	*Denom. (unwt, wt)*	%	(95%CI)	Age-adjusted OR	(95%CI)	p-value	*p-value for interaction with sex[a]*
**Menopausal status**												0.9326	
Not menopausal							*5485, 4187*	32.3%	(30.9-33.8)	1	-		
Menopausal							*1167, 1548*	38.9%	(36.0-41.9)	0.99	(0.79-1.24)		
**Circumcised**						0.5951							
No	*3909, 4728*	15.1%	(13.8-16.4)	1	-								
Yes	*857, 1166*	14.5%	(12.0-17.4)	0.94	(0.73-1.20)								
**<u>Sexual behaviour</u>**													
**Number of occasions of sex, past 4 weeks**						<0.0001						<0.0001	*0.4778*
0	*1013, 1163*	20.7%	(17.8-23.8)	1	-		*1408, 1245*	42.9%	(39.9-45.9)	1	-		
1-2	*1160, 1566*	18.7%	(16.2-21.5)	0.89	(0.69-1.14)		*1481, 1373*	39.6%	(36.7-42.5)	0.89	(0.75-1.05)		
3-4	*870, 1168*	12.4%	(10.1-15.1)	0.54	(0.41-0.73)		*1240, 1130*	33.8%	(30.7-37.0)	0.7	(0.58-0.85)		
5+	*1617, 1869*	9.2%	(7.8-11.0)	0.39	(0.30-0.51)		*2078, 1655*	22.6%	(20.5-24.8)	0.41	(0.34-0.49)		
**Masturbation, past 4 weeks**						0.0458						0.0038	*0.0005*
No	*1297, 1828*	13.7%	(11.8-15.8)	1	-		*4032, 3612*	36.0%	(34.3-37.7)	1	-		
Yes	*3531, 4132*	15.6%	(14.2-17.0)	1.24	(1.00-1.52)		*2615, 2114*	30.8%	(28.7-33.0)	0.83	(0.73-0.94)		
**No. of sexual partners, past year[f]**						0.5348						0.0038	*0.0183*
1	*3573, 4824*	15.0%	(13.7-16.3)	1	-		*5440, 5012*	35.3%	(33.8-36.8)	1	-		
2	*539, 513*	16.2%	(12.9-20.3)	1.14	(0.86-1.52)		*570, 364*	28.2%	(23.9-32.8)	0.80	(0.63-1.01)		
3+	*718, 627*	13.6%	(11.1-16.6)	0.94	(0.72-1.22)		*642, 366*	24.8%	(21.0-29.0)	0.70	(0.56-0.88)		
**Paid for sex, past year**						0.7167							
No	*4774, 5896*	15.0%	(13.9-16.2)	1	-								
Yes	*64, 75*	13.4%	(6.8-24.7)	0.87	(0.41-1.84)								
**Ever taken drugs to assist sexual performance**						0.0175						0.0666	*0.8967*
No	*4188, 5180*	14.4%	(13.2-15.7)	1	-		*6478, 5624*	34.0%	(32.6-35.4)	1	-		
Yes	*636, 776*	19.0%	(15.7-22.8)	1.36	(1.06-1.76)		*184, 124*	40.0%	(32.0-48.5)	1.39	(0.98-1.96)		

For peer review only

For peer review only - http://bmjopen.bmj.com/site/about/guidelines.xhtml

Table 1 cont.

	Men						Women						
	Denom. (unwt, wt)	%	(95%CI)	Age-adjusted OR	(95%CI)	p-value	*Denom. (unwt, wt)*	%	(95%CI)	Age-adjusted OR	(95%CI)	p-value	*p-value for interaction with sex[a]*
**Relationship context**													
**Relationship status**						0.0383						<0.0001	*0.0001*
Living with partner	*2708, 4266*	15.5%	(14.1-17.1)	1	-		*3967, 4168*	37.9%	(36.3-39.7)	1	-		
In a steady relationship, not living together	*947, 760*	12.0%	(9.6-14.8)	0.76	(0.57-1.00)		*1360, 790*	22.6%	(20.2-25.2)	0.51	(0.43-0.60)		
Not in a steady relationship, but previously cohabited	*446, 388*	18.2%	(14.6-22.5)	1.22	(0.91-1.62)		*752, 462*	28.9%	(25.4-32.8)	0.68	(0.56-0.83)		
Not in a steady relationship, never cohabited	*727, 551*	12.4%	(9.9-15.5)	0.8	(0.58-1.09)		*580, 330*	21.3%	(17.6-25.5)	0.49	(0.38-0.63)		
**Duration of most recent sexual relationship**						0.494						<0.0001	*<0.0001*
1 year or less	*1462, 1260*	13.0%	(11.0-15.3)	1	-		*1597, 998*	21.5%	(19.1-24.1)	1	-		
Between 1 and 5 years	*1247, 1227*	15.3%	(13.2-17.7)	1.21	(0.94-1.55)		*1758, 1148*	28.5%	(26.1-31.0)	1.45	(1.20-1.76)		
Between 5 and 15 years	*1065, 1484*	14.9%	(12.6-17.5)	1.14	(0.86-1.50)		*1774, 1458*	39.8%	(37.2-42.4)	2.37	(1.96-2.86)		
Over 15 years	*1004, 1904*	16.1%	(13.9-18.7)	1.19	(0.87-1.63)		*1445, 2036*	40.0%	(37.3-42.7)	2.31	(1.84-2.91)		
**Always easy to talk about sex with partners[g]**						0.0001						<0.0001	*0.0182*
Yes	*1695, 1899*	11.5%	(9.7-13.5)	1	-		*1746, 1451*	22.6%	(20.4-25.1)	1	-		
No/other	*3122, 4048*	16.7%	(15.3-18.2)	1.53	(1.23-1.90)		*4907, 4289*	38.0%	(36.4-39.6)	2.06	(1.77-2.39)		
**Happy with relationship[h]**						<0.0001						<0.0001	*0.8679*
Yes	*1951, 2791*	12.6%	(11.0-14.4)	1	-		*2736, 2601*	31.5%	(29.5-33.6)	1	-		
Other	*995, 1430*	21.0%	(18.4-23.9)	1.85	(1.47-2.32)		*1640, 1617*	45.4%	(42.7-48.1)	1.79	(1.55-2.08)		
**Participant does not share same level of interest in sex as partner**						0.2339						<0.0001	*<0.0001*
No/other	*2270, 3233*	15.0%	(13.4-16.7)	1	-		*3211, 3064*	27.2%	(25.4-29.0)	1	-		
Yes	*676, 988*	17.1%	(14.2-20.4)	1.17	(0.90-1.51)		*1166, 1155*	62.5%	(59.2-65.7)	4.57	(3.87-5.38)		

For peer review only

Table 1 cont.

	Men						Women						
	Denom. (unwt, wt)	%	(95%CI)	Age-adjusted OR	(95%CI)	p-value	*Denom. (unwt, wt)*	%	(95%CI)	Age-adjusted OR	(95%CI)	p-value	*p-value for interaction with sex[a]*
**Participant does not share same sexual likes & dislikes as partner**						0.4188						<0.0001	*<0.0001*
No/other	*2650, 3803*	15.3%	(13.8-16.9)	1	-		*4079, 3908*	34.9%	(33.3-36.6)	1	-		
Yes	*296, 418*	17.3%	(13.0-22.5)	1.16	(0.81-1.66)		*297, 310*	61.0%	(54.6-67.2)	2.91	(2.22-3.83)		
**Partner experienced sexual difficulties in past year**						0.0136						<0.0001	*0.4140*
No/other	*2431, 3454*	14.6%	(13.1-16.2)	1	-		*3726, 3498*	34.8%	(33.1-36.6)	1	-		
Yes	*513, 763*	19.4%	(15.8-23.6)	1.41	(1.07-1.86)		*649, 719*	46.8%	(42.5-51.1)	1.60	(1.32-1.94)		
**Does not feel emotionally close to partner when have sex**						0.0006						<0.0001	*0.5972*
No/other	*2904, 4165*	15.1%	(13.7-16.6)	1	-		*4263, 4108*	35.9%	(34.3-37.6)	1	-		
Yes	*42, 56*	39.9%	(23.6-58.8)	3.74	(1.76-7.93)		*112, 109*	73.0%	(62.8-81.3)	4.80	(2.99-7.69)		
**<u>Lifestyle</u>**													
**1+ child(ren) aged <5 in household**						0.9088						<0.0001	*0.0216*
No, none	*4100, 5015*	15.2%	(13.9-16.5)	1	-		*4997, 4671*	33.1%	(31.6-34.6)	1	-		
Yes, 1+	*727, 941*	14.5%	(11.9-17.6)	0.98	(0.76-1.28)		*1664, 1074*	38.6%	(36.0-41.4)	1.55	(1.34-1.79)		
**Pregnant in the last year**												0.0114	
No							*4227, 4122*	36.2%	(34.6-37.9)	1	-		
Yes							*437, 273*	41.7%	(36.6-47.1)	1.36	(1.07-1.72)		
**Used hormonal contraceptive, past year**												0.05	
No							*3759, 3838*	34.8%	(33.1-36.5)	1	-		
Yes							*2806, 1831*	33.0%	(30.9-35.1)	1.15	(1.00-1.33)		
**<u>Sexual health indicators</u>**													
**Ever diagnosed with a sexually transmitted infection**						<0.0001						0.0004	*0.0651*
No (or only thrush)	*4147, 5127*	14.0%	(12.8-15.3)	1	-		*5455, 4861*	33.4%	(31.9-34.9)	1	-		
Yes (excluding thrush)	*677, 830*	21.4%	(18.1-25.0)	1.67	(1.33-2.10)		*1206, 888*	38.2%	(35.1-41.5)	1.32	(1.13-1.54)		

For peer review only

Table 1 cont.

	Men						Women						
	Denom. (unwt, wt)	%	(95%CI)	Age-adjusted OR	(95%CI)	p-value	*Denom. (unwt, wt)*	%	(95%CI)	Age-adjusted OR	(95%CI)	p-value	*p-value for interaction with sex[a]*
**Ever experienced non-volitional sex**						0.0010						<0.0001	*0.3164*
No	*4705, 5824*	14.7%	(13.6-16.0)	1	-		*5815, 5055*	32.8%	(31.4-34.2)	1	-		
Yes/Don't know	*133, 148*	26.1%	(18.9-34.9)	2.07	(1.34-3.18)		*848, 695*	44.3%	(40.5-48.3)	1.66	(1.40-1.97)		
**Sexual competence at first sex[i]**						0.0706						<0.0001	*0.1797*
Not competent	*2407, 3037*	16.2%	(14.6-17.9)	1	-		*3438, 2927*	37.6%	(35.7-39.5)	1	-		
Competent	*2302, 2784*	13.7%	(12.1-15.4)	0.84	(0.69-1.01)		*3097, 2716*	30.3%	(28.4-32.3)	0.73	(0.65-0.83)		
**Number of other sexual response problems experienced[j]**						<0.0001						<0.0001	*0.0015*
0	*3208, 3945*	11.7%	(10.5-13.1)	1	-		*4377, 3759*	25.3%	(23.8-26.9)	1	-		
1	*1061, 1350*	10.9%	(9.0-13.2)	0.91	(0.71-1.17)		*1217, 1087*	34.8%	(31.7-38.0)	1.55	(1.32-1.82)		
2+	*570, 678*	42.5%	(37.9-47.2)	5.58	(4.41-7.04)		*1075, 909*	69.8%	(66.5-72.9)	6.91	(5.82-8.21)		
**<u>Attitudes</u>**													
**People are under pressure to have sex**						0.0115						0.0001	*0.7970*
Else	*1799, 2264*	13.1%	(11.4-15.0)	1	-		*1851, 1570*	29.3%	(26.8-31.9)	1	-		
Strongly agree/agree	*3038, 3707*	16.2%	(14.7-17.8)	1.29	(1.06-1.57)		*4817, 4185*	36.0%	(34.4-37.6)	1.34	(1.16-1.54)		
**People want less sex as they age**						<0.0001						<0.0001	*0.9443*
Else	*2943, 3472*	11.4%	(10.2-12.8)	1	-		*4044, 3278*	27.8%	(26.2-29.4)	1	-		
Strongly agree/agree	*1894, 2499*	20.0%	(18.0-22.2)	1.93	(1.61-2.32)		*2624, 2477*	42.6%	(40.4-44.8)	1.85	(1.63-2.10)		
**Men have a naturally higher sex drive than women**						<0.0001						<0.0001	*<0.0001*
Else	*2788, 3441*	18.0%	(16.4-19.7)	1	-		*3351, 2830*	26.0%	(24.3-27.8)	1	-		
Strongly agree/agree	*2049, 2530*	10.9%	(9.4-12.6)	0.56	(0.46-0.68)		*3317, 2925*	42.0%	(40.0-44.1)	2.04	(1.80-2.31)		
**Too much sex in the media**						0.7069						0.1807	*0.4835*
Else	*1986, 2296*	14.6%	(12.8-16.6)	1	-		*2091, 1618*	31.7%	(29.3-34.2)	1	-		
Strongly agree/agree	*2851, 3675*	15.3%	(13.8-16.9)	1.04	(0.85-1.26)		*4577, 4137*	35.1%	(33.5-36.8)	1.10	(0.96-1.26)		

For peer review only

Denominator is those aged 16-74 years with at least one partner in the past year. Unwt, unweighted; wt, weighted

[a] P-value for interaction to determine whether the magnitude of association between each variable and lack of interest in sex differs between men and women

[b] IMD is a multidimensional measure of area (neighbourhood)-level deprivation based on the participant's postcode. IMD scores for England, Scotland and Wales were adjusted before being combined and assigned to quintiles, using a method by Payne and Abel (50).

[c] Participants aged ≥17 years.

[d] Includes arthritis, heart attack, coronary heart disease, angina, other forms of heart disease, hypertension, stroke, diabetes, broken hip or pelvis, bone or hip replacement ever, backache lasting longer than 3 months, any other muscle or bone disease lasting longer than 3 months, depression, cancer and any thyroid condition treated in the past year.

[e] Participants were asked whether they had often been bothered by feeling down, depressed, or hopeless in the past 2 weeks, and whether they had often been bothered by little interest or pleasure in doing things in the past 2 weeks, using a validated two-question patient health questionnaire (PHQ-2).

[f] Opposite and/or same-sex partners

[g] Other means easy with a husband or wife or regular partner, but difficult with a new partner; easy with a new partner, but difficult with a husband or wife or regular partner; difficult with any partner, it depends, sometimes easy, and sometimes difficult.

[h] Participants were asked to rate how happy they were in their relationship from 1 (very happy) to 7 (very unhappy); responses of 1 or 2 were regarded as denoting participants who were happy with their relationship.

[i] A constructed variable to measure readiness, combining consensuality, autonomy of decision making, timing and use of effective contraception

[j] Sexual response problems (for at least 3 months in past year): lacked enjoyment in sex, felt anxious during sex, felt physical pain as a result of sex, felt no excitement or arousal during sex, difficulty in reaching climax, reached a climax more quickly than you would like, trouble getting or keep an erection (men), uncomfortably dry vagina (women)

For peer review only

Table 2: Factors associated with lacking interest in having sex for at least 3 months in the past year and being distressed about it in sexually active men and women

	Men						Women						
	Denom. (unwt, wt)	%	(95%CI)	Age-adjusted OR	(95%CI)	p-value	*Denom. (unwt, wt)*	%	(95%CI)	Age-adjusted OR	(95%CI)	p-value	*p-value for interaction with sex[a]*
**All**	*4839, 5973*	**8.2%**	**(7.4-9.1)**				*6669, 5755*	**20.8%**	**(19.6-22.0)**				
**Socio-demographics**													
**Age group**						0.0011						<0.0001	*0.8971*
16-24	*1279, 936*	4.8%	(3.7-6.4)	1	-		*1662, 923*	15.2%	(13.4-17.3)	1	-		
25-34	*1376, 1238*	8.0%	(6.7-9.5)	1.7	(1.19-2.41)		*2236, 1246*	20.9%	(19.0-22.8)	1.47	(1.22-1.76)		
35-44	*719, 1298*	9.6%	(7.5-12.3)	2.09	(1.40-3.13)		*1050, 1290*	22.9%	(20.3-25.7)	1.65	(1.34-2.04)		
45-54	*630, 1186*	9.7%	(7.4-12.6)	2.11	(1.38-3.22)		*871, 1186*	23.3%	(20.4-26.6)	1.69	(1.35-2.13)		
55-64	*512, 849*	9.4%	(7.0-12.6)	2.04	(1.30-3.21)		*569, 755*	21.8%	(18.3-25.8)	1.55	(1.20-2.01)		
65-74	*323, 467*	5.5%	(3.4-8.6)	1.13	(0.65-1.99)		*281, 355*	16.5%	(12.4-21.7)	1.10	(0.76-1.59)		
**Index of Multiple Deprivation (quintiles)[b]**						0.8339						0.0938	*0.4592*
1 (least deprived)	*977, 1279*	8.1%	(6.2-10.4)	1	-		*1248, 1208*	23.3%	(20.7-26.1)	1	-		
2	*962, 1264*	7.4%	(5.7-9.6)	0.92	(0.62-1.36)		*1290, 1208*	20.8%	(18.2-23.5)	0.87	(0.69-1.09)		
3	*942, 1169*	8.3%	(6.4-10.6)	1.05	(0.71-1.55)		*1299, 1116*	19.6%	(17.1-22.4)	0.82	(0.65-1.03)		
4	*967, 1184*	8.8%	(6.9-11.1)	1.14	(0.78-1.66)		*1384, 1137*	21.9%	(19.3-24.7)	0.95	(0.76-1.18)		
5 (most deprived)	*991, 1077*	8.6%	(6.7-10.9)	1.12	(0.75-1.65)		*1448, 1086*	18.2%	(15.9-20.6)	0.75	(0.60-0.94)		
**Education level[c]**						0.4958						0.7324	0.4496
Left school aged 17+	*2862, 3464*	7.9%	(6.8-9.2)	1	-		*4150, 3406*	20.8%	(19.4-22.4)	1	-		
Left school at 16	*1873, 2437*	8.8%	(7.5-10.4)	1.09	(0.85-1.41)		*2409, 2287*	21.1%	(19.3-23.1)	0.97	(0.83-1.14)		
**Employment status**						0.0001						0.0003	*0.1244*
Employed	*3211, 4254*	8.3%	(7.3-9.5)	1	-		*3871, 3517*	21.6%	(20.1-23.2)	1	-		
Full-time education	*542, 431*	4.9%	(2.8-8.6)	0.74	(0.38-1.44)		*693, 423*	14.8%	(11.8-18.4)	0.75	(0.56-1.01)		
Unemployed	*707, 723*	12.1%	(9.5-15.3)	1.56	(1.14-2.13)		*1681, 1282*	22.3%	(19.9-24.9)	1.07	(0.90-1.27)		
Retired	*375, 562*	4.9%	(3.1-7.6)	0.41	(0.23-0.71)		*415, 524*	16.8%	(13.4-20.8)	0.57	(0.41-0.79)		
**Practises religion at least once a month**						0.1638						0.0167	*0.8143*
No	*4283, 5179*	8.5%	(7.5-9.5)	1	-		*5659, 4754*	21.5%	(20.2-22.9)	1	-		
Yes	*521, 748*	6.4%	(4.4-9.4)	0.73	(0.48-1.13)		*956, 945*	18.0%	(15.3-20.9)	0.78	(0.63-0.96)		

For peer review only

Table 2 cont.

	Men						Women						
	Denom. (unwt, wt)	%	(95%CI)	Age-adjusted OR	(95%CI)	p-value	Denom. (unwt, wt)	%	(95%CI)	Age-adjusted OR	(95%CI)	p-value	*p-value for interaction with sex*[a]
**Health**													
**Self-reported general health**						<0.0001						<0.0001	*0.0969*
Very good/Good	*4123, 5055*	7.0%	(6.1-7.9)	1	-		*5683, 4851*	19.2%	(18.0-20.5)	1	-		
Fair	*580, 745*	13.3%	(10.5-16.8)	2.04	(1.50-2.78)		*780, 709*	27.9%	(24.3-31.9)	1.60	(1.30-1.97)		
Bad/very bad	*135, 171*	22.6%	(15.3-32.1)	3.85	(2.31-6.40)		*206, 195*	33.4%	(26.3-41.4)	2.05	(1.45-2.91)		
**Difficulty walking up stairs because of a health problem**						0.0001						0.0085	*0.1553*
No difficulty	*4475, 5460*	7.6%	(6.7-8.6)	1	-		*6062, 5107*	20.1%	(18.9-21.4)	1	-		
Some difficulty	*278, 393*	12.3%	(8.8-17.0)	1.67	(1.11-2.52)		*450, 482*	24.1%	(20.0-28.7)	1.21	(0.93-1.59)		
Much difficulty/unable to do this	*86, 120*	22.2%	(13.5-34.2)	3.36	(1.79-6.32)		*157, 166*	32.3%	(24.3-41.5)	1.81	(1.21-2.70)		
**Longstanding illness or disability**						<0.0001						<0.0001	*0.0345*
No	*3585, 4259*	6.5%	(5.6-7.5)	1	-		*4843, 4026*	18.7%	(17.4-20.0)	1	-		
Yes	*1253, 1713*	12.5%	(10.6-14.8)	2.09	(1.60-2.74)		*1825, 1729*	25.7%	(23.4-28.2)	1.48	(1.27-1.74)		
**Number of comorbid conditions**[d]						<0.0001						<0.0001	*0.5779*
0	*3453, 3994*	6.4%	(5.5-7.5)	1	-		*4357, 3536*	17.3%	(15.9-18.7)	1	-		
1	*939, 1329*	11.0%	(9.0-13.4)	1.88	(1.37-2.57)		*1555, 1416*	24.1%	(21.7-26.7)	1.54	(1.30-1.83)		
>=2	*446, 650*	13.3%	(10.1-17.4)	2.40	(1.61-3.59)		*755, 802*	30.5%	(26.8-34.4)	2.16	(1.74-2.69)		
**Depressive symptoms**[e]						<0.0001						<0.0001	*0.0370*
No	*4383, 5471*	6.8%	(6.0-7.7)	1	-		*5885, 5149*	18.6%	(17.4-19.8)	1	-		
Yes	*449, 495*	23.7%	(19.3-28.9)	4.36	(3.20-5.94)		*780, 602*	39.6%	(35.4-44.0)	2.94	(2.41-3.59)		
**Treated for depression, past year**						<0.0001						<0.0001	*0.0371*
No	*4524, 5630*	7.3%	(6.5-8.2)	1	-		*5770, 5040*	18.5%	(17.3-19.8)	1	-		
Yes	*313, 342*	23.0%	(17.9-29.1)	3.81	(2.71-5.36)		*897, 713*	36.4%	(32.9-40.2)	2.54	(2.12-3.03)		

For peer review only

Table 2 cont.

	Men						Women						
	Denom. (unwt, wt)	%	(95%CI)	Age-adjusted OR	(95%CI)	p-value	*Denom. (unwt, wt)*	%	(95%CI)	Age-adjusted OR	(95%CI)	p-value	*p-value for interaction with sex[a]*
**Menopausal status**												0.9656	
Not menopausal							*5485, 4187*	20.2%	(18.9-21.5)	1	-		
Menopausal							*1167, 1548*	22.5%	(20.0-25.2)	1.01	(0.76-1.32)		
**Circumcised**						0.4097							
No	*3909, 4728*	8.3%	(7.4-9.4)	1	-								
Yes	*857, 1166*	7.5%	(5.7-9.9)	0.87	(0.62-1.22)								
**<u>Sexual behaviour</u>**													
**Number of occasions of sex, past 4 weeks**						<0.0001						<0.0001	*0.5496*
0	*1013, 1163*	10.3%	(8.3-12.7)	1	-		*1408, 1245*	23.2%	(20.7-26.0)	1	-		
1-2	*1160, 1566*	10.5%	(8.6-12.8)	1.02	(0.74-1.42)		*1481, 1373*	24.2%	(21.8-26.9)	1.06	(0.87-1.30)		
3-4	*870, 1168*	7.4%	(5.6-9.8)	0.71	(0.48-1.04)		*1240, 1130*	21.3%	(18.7-24.2)	0.91	(0.73-1.13)		
5+	*1617, 1869*	5.0%	(3.9-6.4)	0.46	(0.33-0.66)		*2078, 1655*	14.7%	(12.9-16.7)	0.58	(0.47-0.72)		
**Masturbation, past 4 weeks**						0.0164						0.7265	*0.0309*
No	*1297, 1828*	6.9%	(5.5-8.6)	1	-		*4032, 3612*	21.1%	(19.6-22.6)	1	-		
Yes	*3531, 4132*	8.8%	(7.7-9.9)	1.42	(1.07-1.88)		*2615, 2114*	20.3%	(18.4-22.2)	0.97	(0.84-1.13)		
**No. of sexual partners, past year[f]**						0.2466						0.0016	*0.4744*
1	*3573, 4824*	8.5%	(7.5-9.6)	1	-		*5440, 5012*	21.6%	(20.3-22.9)	1	-		
2	*539, 513*	6.3%	(4.3-9.1)	0.75	(0.49-1.14)		*570, 364*	16.7%	(13.3-20.6)	0.75	(0.57-0.99)		
3+	*718, 627*	6.8%	(5.1-9.0)	0.82	(0.59-1.15)		*642, 366*	14.1%	(11.0-17.7)	0.62	(0.46-0.83)		
**Paid for sex, past year**						0.4865							
No	*4774, 5896*	8.2%	(7.4-9.2)	1	-								
Yes	*64, 75*	5.6%	(1.8-16.4)	0.66	(0.20-2.15)								
**Ever taken drugs to assist sexual performance**						0.0022						0.1055	*0.5305*
No	*4188, 5180*	7.6%	(6.7-8.6)	1	-		*6478, 5624*	20.6%	(19.5-21.8)	1	-		
Yes	*636, 776*	12.1%	(9.5-15.4)	1.63	(1.19-2.23)		*184, 124*	25.9%	(19.2-33.9)	1.38	(0.93-2.05)		

For peer review only

For peer review only - http://bmjopen.bmj.com/site/about/guidelines.xhtml

Table 2 cont.

	Men						Women						
	Denom. (unwt, wt)	%	(95%CI)	Age-adjusted OR	(95%CI)	p-value	*Denom. (unwt, wt)*	%	(95%CI)	Age-adjusted OR	(95%CI)	p-value	*p-value for interaction with sex[a]*
**Relationship context**													
**Relationship status**						0.03						<0.0001	*0.0307*
Living with partner	*2708, 4266*	8.8%	(7.7-10.1)	1	-		*3967, 4168*	23.4%	(21.9-24.9)	1	-		
In a steady relationship, not living together	*947, 760*	6.9%	(5.3-9.0)	0.78	(0.56-1.09)		*1360, 790*	15.4%	(13.4-17.7)	0.59	(0.49-0.71)		
Not in a steady relationship, but previously cohabited	*446, 388*	8.8%	(6.2-12.2)	1.00	(0.67-1.48)		*752, 462*	13.6%	(11.1-16.6)	0.51	(0.40-0.66)		
Not in a steady relationship, never cohabited	*727, 551*	4.7%	(3.3-6.8)	0.52	(0.34-0.81)		*580, 330*	11.0%	(8.2-14.5)	0.39	(0.28-0.55)		
**Duration of most recent sexual relationship**						0.0143						<0.0001	*0.0719*
1 year or less	*1462, 1260*	5.5%	(4.3-7.1)	1	-		*1597, 998*	11.2%	(9.4-13.2)	1	-		
Between 1 and 5 years	*1247, 1227*	9.0%	(7.3-11.0)	1.67	(1.18-2.36)		*1758, 1148*	18.5%	(16.5-20.7)	1.81	(1.44-2.29)		
Between 5 and 15 years	*1065, 1484*	9.3%	(7.5-11.6)	1.68	(1.17-2.43)		*1774, 1458*	25.2%	(23.0-27.6)	2.81	(2.23-3.55)		
Over 15 years	*1004, 1904*	8.8%	(7.1-10.8)	1.47	(0.97-2.22)		*1445, 2036*	23.8%	(21.5-26.2)	2.83	(2.13-3.75)		
**Always easy to talk about sex with partners[g]**						0						<0.0001	*0.4854*
Yes	*1695, 1899*	4.8%	(3.8-6.0)	1	-		*1746, 1451*	11.4%	(9.7-13.2)	1	-		
No/other	*3122, 4048*	9.8%	(8.7-11.1)	2.15	(1.62-2.87)		*4907, 4289*	23.9%	(22.5-25.3)	2.43	(2.02-2.93)		
**Happy with relationship[h]**						<0.0001						<0.0001	*0.9717*
Yes	*1951, 2791*	7.1%	(5.9-8.6)	1	-		*2736, 2601*	18.6%	(16.9-20.4)	1	-		
Other	*995, 1430*	13.3%	(11.2-15.8)	2.01	(1.51-2.66)		*1640, 1617*	31.4%	(28.8-34.0)	2.00	(1.69-2.37)		
**Participant does not share same level of interest in sex as partner**							0.0311						<0.0001
No/other	*2270, 3233*	8.5%	(7.2-10.0)	1	-		*3211, 3064*	15.0%	(13.6-16.4)	1	-		
Yes	*676, 988*	11.6%	(9.2-14.4)	1.41	(1.03-1.92)		*1166, 1155*	46.2%	(42.9-49.6)	4.91	(4.13-5.83)		

For peer review only

Table 2 cont.

	Men						Women						
	Denom. (unwt, wt)	%	(95%CI)	Age-adjusted OR	(95%CI)	p-value	Denom. (unwt, wt)	%	(95%CI)	Age-adjusted OR	(95%CI)	p-value	*p-value for interaction with sex*[a]
**Participant does not share same sexual likes & dislikes as partner**						0.0975						<0.0001	*0.0212*
No/other	*2650, 3803*	8.9%	(7.7-10.2)	1	-		*4079, 3908*	22.1%	(20.6-23.6)	1	-		
Yes	*296, 418*	12.2%	(8.6-17.0)	1.43	(0.94-2.18)		*297, 310*	41.9%	(35.6-48.6)	2.55	(1.93-3.37)		
**Partner experienced sexual difficulties in past year**						0.0027						<0.0001	*0.6889*
No/other	*2431, 3454*	8.3%	(7.2-9.6)	1	-		*3726, 3498*	22.1%	(20.6-23.7)	1	-		
Yes	*513, 763*	13.2%	(10.2-17.0)	1.68	(1.20-2.35)		*649, 719*	30.4%	(26.5-34.6)	1.58	(1.27-1.95)		
**Does not feel emotionally close to partner when have sex**						0.0225						<0.0001	*0.8228*
No/other	*2904, 4165*	9.1%	(7.9-10.3)	1	-		*4263, 4108*	22.9%	(21.5-24.4)	1	-		
Yes	*42, 56*	21.0%	(10.2-38.3)	2.69	(1.15-6.29)		*112, 109*	47.0%	(36.4-57.8)	2.98	(1.92-4.63)		
**<u>Lifestyle</u>**													
**1+ child(ren) aged <5 in household**						0.1047						0.0004	*0.0042*
No, none	*4100, 5015*	8.6%	(7.6-9.6)	1	-		*4997, 4671*	20.2%	(18.9-21.5)	1	-		
Yes, 1+	*727, 941*	6.3%	(4.6-8.5)	0.75	(0.52-1.06)		*1664, 1074*	23.5%	(21.2-25.9)	1.34	(1.14-1.58)		
**Pregnant in the last year**												0.5927	
No							*4227, 4122*	21.8%	(20.4-23.4)	1	-		
Yes							*437, 273*	20.7%	(16.6-25.6)	0.92	(0.69-1.24)		
**Used hormonal contraceptive, past year**												0.1141	
No							*3759, 3838*	20.7%	(19.2-22.3)	1	-		
Yes							*2806, 1831*	20.9%	(19.1-22.7)	1.14	(0.97-1.35)		
**<u>Sexual health indicators</u>**													
**Ever diagnosed with a sexually transmitted infection**						<0.0001						0.0002	*0.0291*
No (or only thrush)	*4148, 5128*	7.3%	(6.5-8.3)	1	-		*5455, 4861*	20.0%	(18.7-21.3)	1	-		
Yes (excluding thrush)	*677, 830*	13.7%	(11.0-17.0)	2.02	(1.51-2.70)		*1206, 888*	25.1%	(22.3-28.1)	1.39	(1.16-1.65)		

For peer review only

Table 2 cont.

	Men						Women						
	Denom. (unwt, wt)	%	(95%CI)	Age-adjusted OR	(95%CI)	p-value	*Denom. (unwt, wt)*	%	(95%CI)	Age-adjusted OR	(95%CI)	p-value	*p-value for interaction with sex*[a]
**Ever experienced non-volitional sex**						<0.0001						<0.0001	*0.1143*
No	*4706, 5825*	7.9%	(7.1-8.9)	1	-		*5815, 5055*	19.4%	(18.2-20.7)	1	-		
Yes/Don't know	*133, 148*	19.4%	(13.1-27.7)	2.83	(1.74-4.59)		*848, 695*	30.9%	(27.3-34.6)	1.86	(1.55-2.25)		
**Sexual competence at first sex**[i]						0.4876						<0.0001	*0.0787*
Not competent	*2408, 3039*	8.7%	(7.5-10.0)	1	-		*3438, 2927*	23.6%	(21.9-25.3)	1	-		
Competent	*2302, 2784*	7.8%	(6.6-9.2)	0.91	(0.71-1.18)		*3097, 2716*	17.7%	(16.1-19.3)	0.70	(0.61-0.81)		
**Number of other sexual response problems experienced**[j]						<0.0001						<0.0001	*0.0262*
0	*3209, 3947*	5.3%	(4.4-6.3)	1	-		*4377, 3759*	12.9%	(11.7-14.1)	1	-		
1	*1061, 1350*	6.1%	(4.7-7.8)	1.14	(0.81-1.59)		*1217, 1087*	21.7%	(19.0-24.6)	1.86	(1.53-2.26)		
2+	*570, 678*	29.7%	(25.4-34.4)	7.57	(5.68-10.10)		*1075, 909*	52.4%	(48.9-56.0)	7.48	(6.25-8.94)		
**Attitudes**													
**People are under pressure to have sex**						0.1437						<0.0001	*0.2192*
Else	*1799, 2264*	7.4%	(6.0-9.0)	1	-		*1851, 1570*	16.4%	(14.5-18.5)	1	-		
Strongly agree/agree	*3038, 3707*	8.7%	(7.6-9.9)	1.21	(0.94-1.57)		*4817, 4185*	22.4%	(21.0-23.9)	1.47	(1.24-1.74)		
**People want less sex as they age**						0.0005						<0.0001	*0.8045*
Else	*2943, 3472*	6.7%	(5.7-7.8)	1	-		*4044, 3278*	17.1%	(15.8-18.6)	1	-		
Strongly agree/agree	*1894, 2499*	10.3%	(8.8-12.1)	1.58	(1.22-2.04)		*2624, 2477*	25.6%	(23.7-27.6)	1.64	(1.43-1.90)		
**Men have a naturally higher sex drive than women**							<0.0001						<0.0001
Else	*2788, 3441*	10.2%	(8.9-11.5)	1	-		*3351, 2830*	15.9%	(14.4-17.4)	1	-		
Strongly agree/agree	*2049, 2530*	5.5%	(4.4-6.9)	0.52	(0.39-0.68)		*3317, 2925*	25.5%	(23.8-27.4)	1.81	(1.56-2.09)		
**Too much sex in the media**						0.3477						0.0693	*0.8856*
Else	*1986, 2296*	7.5%	(6.3-9.0)	1	-		*2091, 1618*	18.8%	(16.8-20.9)	1	-		
Strongly agree/agree	*2851, 3675*	8.6%	(7.5-9.9)	1.13	(0.88-1.46)		*4577, 4137*	21.6%	(20.2-23.0)	1.16	(0.99-1.36)		

Denominator is those aged 16-74 years with at least one partner in the past year. Unwt, unweighted; wt, weighted

[a] P-value for interaction to determine whether the magnitude of association between each variable and lack of interest in sex differs between men and women

For peer review only

[b] IMD is a multidimensional measure of area (neighbourhood)-level deprivation based on the participant's postcode. IMD scores for England, Scotland and Wales were adjusted before being combined and assigned to quintiles, using a method by Payne and Abel (50).

[c] Participants aged ≥17 years.

[d] Includes arthritis, heart attack, coronary heart disease, angina, other forms of heart disease, hypertension, stroke, diabetes, broken hip or pelvis, bone or hip replacement ever, backache lasting longer than 3 months, any other muscle or bone disease lasting longer than 3 months, depression, cancer and any thyroid condition treated in the past year.

[e] Participants were asked whether they had often been bothered by feeling down, depressed, or hopeless in the past 2 weeks, and whether they had often been bothered by little interest or pleasure in doing things in the past 2 weeks, using a validated two-question patient health questionnaire (PHQ-2).

[f] Opposite and/or same-sex partners

[g] Other means easy with a husband or wife or regular partner, but difficult with a new partner; easy with a new partner, but difficult with a husband or wife or regular partner; difficult with any partner, it depends, sometimes easy, and sometimes difficult.

[h] Participants were asked to rate how happy they were in their relationship from 1 (very happy) to 7 (very unhappy); responses of 1 or 2 were regarded as denoting participants who were happy with their relationship.

[i] A constructed variable to measure readiness, combining consensuality, autonomy of decision making, timing and use of effective contraception

[j] Sexual response problems (for at least 3 months in past year): lacked enjoyment in sex, felt anxious during sex, felt physical pain as a result of sex, felt no excitement or arousal during sex, difficulty in reaching climax, reached a climax more quickly than you would like, trouble getting or keep an erection (men), uncomfortably dry vagina (women)

For peer review only

For peer review only

Table 3: Associations between reporting lack of interest in having sex for at least 3 months in the past year and other sexual response problems lasting 3 months or more in the past year, by sex

		Men							Women						
		Did not report a lack interest in sex		Reported a lack of interest in sex			(95%CI)	p-value	Did not report a lack of interest in sex		Reported a lack of interest in sex			(95%CI)	p-value
Denominators (unwt, wt)		4126, 5077		713, 897		AOR[a]			4540, 3790		2129, 1965		AOR[a]		
**Lacked enjoyment in having sex**								<0.0001							<0.0001
	No	97.7%	(97.1-98.1)	81.5%	(78.2-84.4)	1	-		95.9%	(95.1-96.5)	72.5%	(70.2-74.7)	1	-	
	Yes	2.3%	(1.9-2.9)	18.5%	(15.6-21.8)	9.78	(7.11-13.46)		4.1%	(3.5-4.9)	27.5%	(25.3-29.8)	8.95	(7.28-11.01)	
**Felt anxious during sex**								<0.0001							<0.0001
	No	96.1%	(95.5-96.7)	85.8%	(82.6-88.5)	1	-		97.3%	(96.7-97.7)	89.9%	(88.4-91.3)	1	-	
	Yes	3.9%	(3.3-4.5)	14.2%	(11.5-17.4)	4.16	(3.08-5.62)		2.7%	(2.3-3.3)	10.1%	(8.7-11.6)	4.4	(3.43-5.65)	
**Felt physical pain as a result of sex**								0.0213							<0.0001
	No	98.4%	(97.9-98.8)	97.1%	(95.6-98.1)	1	-		95.7%	(95.0-96.3)	86.5%	(84.6-88.1)	1	-	
	Yes	1.6%	(1.2-2.1)	2.9%	(1.9-4.4)	1.87	(1.10-3.19)		4.3%	(3.7-5.0)	13.5%	(11.9-15.4)	3.55	(2.83-4.45)	
**Felt no excitement or arousal during sex**								<0.0001							<0.0001
	No	98.5%	(98.0-98.9)	87.7%	(85.0-90.0)	1	-		97.5%	(96.9-97.9)	80.9%	(79.0-82.7)	1	-	
	Yes	1.5%	(1.1-2.0)	12.3%	(10.0-15.0)	9.21	(6.33-13.40)		2.5%	(2.1-3.1)	19.1%	(17.3-21.0)	9.16	(7.16-11.70)	
**Difficulty in reaching climax**								<0.0001							<0.0001
	No	92.7%	(91.7-93.5)	80.5%	(76.6-83.8)	1	-		88.3%	(87.2-89.3)	74.9%	(72.7-76.9)	1	-	
	Yes	7.3%	(6.5-8.3)	19.5%	(16.2-23.4)	3.08	(2.37-3.99)		11.7%	(10.7-12.8)	25.1%	(23.1-27.3)	2.6	(2.23-3.03)	
**Reached climax more quickly than you would like**								0.0198							0.3658
	No	85.6%	(84.3-86.9)	82.0%	(78.7-85.0)	1	-		97.8%	(97.2-98.2)	97.5%	(96.7-98.1)	1	-	
	Yes	14.4%	(13.1-15.7)	18.0%	(15.0-21.3)	1.32	(1.05-1.68)		2.2%	(1.8-2.8)	2.5%	(1.9-3.3)	1.18	(0.82-1.69)	
**Trouble getting or keeping an erection**								<0.0001							
	No	88.5%	(87.3-89.6)	79.4%	(75.9-82.6)	1	-								
	Yes	11.5%	(10.4-12.7)	20.6%	(17.4-24.1)	1.97	(1.55-2.51)								
**Uncomfortably dry vagina**															<0.0001
	No								90.7%	(89.5-91.7)	80.1%	(77.9-82.1)	1	-	
	Yes								9.3%	(8.3-10.5)	19.9%	(17.9-22.1)	2.28	(1.89-2.76)	

Denominator is those aged 16-74 years with at least one partner in the past year. Unwt, unweighted; wt, weighted

[a]AOR comparing those reporting lacking interest to those who did not.

STROBE Statement—checklist of items that should be included in reports of observational studies

	Item No.	Recommendation	Page No.	Relevant text from manuscript
**Title and abstract**	1	(*a*) Indicate the study's design with a commonly used term in the title or the abstract	Title, Abstract	Survey; cross-sectional probability sample survey
		(*b*) Provide in the abstract an informative and balanced summary of what was done and what was found	Abstract	Complex survey analyses of data collected for a cross-sectional probability sample survey, undertaken 2010-12, specifically logistic regression to calculate age-adjusted odds ratios (AOR) to identify associated factors
**Introduction**				
Background/rationale	2	Explain the scientific background and rationale for the investigation being reported	4-5	In summary, the evidence on the factors associated with men's and women's reports of low sexual desire is drawn largely from non-representative samples, is somewhat equivocal and, in men, sparse. Understanding the correlates of lacking interest in sex would be useful to identify individuals most at risk of sexual problems and could also inform what types of therapeutic options for this group should be provided.
Objectives	3	State specific objectives, including any prespecified hypotheses	5	The research questions addressed in this paper are: (1)

For peer review only - http://bmjopen.bmj.com/site/about/guidelines.xhtml

				What sociodemographic, relationship, sexual behaviour, and sexual attitudinal factors are associated with lacking interest in sex in sexually active men and women?; (2) To what extent do these factors vary by gender?; (3) What are the associations between reporting lacking interest in sex and other sexual function problems?
**Methods**				
Study design	4	Present key elements of study design early in the paper	6	A multi-stage, clustered, and stratified probability sample design was used and participants were interviewed using a combination of computer-assisted personal interviews (CAPI), and computer-assisted self-interviews (CASI).
Setting	5	Describe the setting, locations, and relevant dates, including periods of recruitment, exposure, follow-up, and data collection	6	Natsal-3 is a probability sample survey of 15,162 men and women aged 16-74 years in Britain, interviewed between September 2010 and August 2012.
Participants	6	(*a*) *Cohort study*—Give the eligibility criteria, and the sources and methods of selection of participants. Describe methods of follow-up<br>*Case-control study*—Give the eligibility criteria, and the sources and methods of case	6	For the current analyses, only respondents who reported >=1 sexual partner (opposite-sex or

For peer review only

For peer review only - http://bmjopen.bmj.com/site/about/guidelines.xhtml

		ascertainment and control selection. Give the rationale for the choice of cases and controls<br>*Cross-sectional study*—Give the eligibility criteria, and the sources and methods of selection of participants		same-sex) in the past year were included (4,839 men and 6,669 women).
		(*b*) *Cohort study*—For matched studies, give matching criteria and number of exposed and unexposed<br>*Case-control study*—For matched studies, give matching criteria and the number of controls per case		
Variables	7	Clearly define all outcomes, exposures, predictors, potential confounders, and effect modifiers. Give diagnostic criteria, if applicable	6-7	We used selected items from the Natsal-SF, a newly developed and validated measure of sexual function comprising questions about problems with sexual response, relational aspects of sexual function, and self-appraisal of sex life
Data sources/ measurement	8*	For each variable of interest, give sources of data and details of methods of assessment (measurement). Describe comparability of assessment methods if there is more than one group	6-7	In the CASI, participants who reported at least one sexual partner in the past year (hereon 'sexually active participants') were asked: *'In the last year, have you experienced any of the following for a period of 3 months or longer?'* and were given a list of difficulties and asked to indicate which they had experienced. The list included *'Lacked interest in having sex'*. Those indicating this difficulty were defined as lacking interest in having sex for a period of

For peer review only - http://bmjopen.bmj.com/site/about/guidelines.xhtml

				three months or more in the past year (the outcome for this analysis). Individuals reporting lacking interest in sex for at least 3 months were then asked *'And how do you feel about this?'* with response options: *not at all distressed, a little distressed, fairly distressed; very distressed*. Those answering a little, fairly or very distressed were defined as lacking interest in sex and having distress about this symptom (outcome for sensitivity analysis, see below).
Bias	9	Describe any efforts to address potential sources of bias	6	After weighting to adjust for unequal probabilities of selection and to match the British population in terms of age, gender and geographical region, the Natsal-3 sample was broadly representative, on key variables, of the British population as described by the 2011 Census.
Study size	10	Explain how the study size was arrived at		-

Continued on next page

For peer review only

Quantitative variables	11	Explain how quantitative variables were handled in the analyses. If applicable, describe which groupings were chosen and why	7	All analyses were done using the complex survey functions of STATA (version 14; StataCorp LP, College Station, Texas) to account for the weighting, clustering, and stratification of the data.
Statistical methods	12	(*a*) Describe all statistical methods, including those used to control for confounding	7	We used multivariable logistic regression to calculate age-adjusted odds ratios (AOR) to examine the associations between reports of lacking interest in sex lasting three months or longer in the past year, and sociodemographic, health, relationship, sexual behaviour, and sexual attitude variables. For each variable, we also tested the interaction between gender, to see if the magnitude of the associations between the above factors and reports of lacking interest in sex was the same for men and women.
		(*b*) Describe any methods used to examine subgroups and interactions	7	-
		(*c*) Explain how missing data were addressed	-	-
		(*d*) *Cohort study*—If applicable, explain how loss to follow-up was addressed<br>*Case-control study*—If applicable, explain how matching of cases and controls was addressed<br>*Cross-sectional study*—If applicable, describe analytical methods taking account of sampling strategy		
		(*e*) Describe any sensitivity analyses	7	We conducted a sensitivity analysis for the outcome variable reporting lack of interest in sex lasting three months or longer *and* distress about

For peer review only

				this symptom to assess whether similar associations were found.
**Results**				
Participants	13*	(a) Report numbers of individuals at each stage of study—eg numbers potentially eligible, examined for eligibility, confirmed eligible, included in the study, completing follow-up, and analysed	6	For the current analyses, only respondents who reported >=1 sexual partner (opposite-sex or same-sex) in the past year were included (4,839 men and 6,669 women).
		(b) Give reasons for non-participation at each stage	-	
		(c) Consider use of a flow diagram	-	
Descriptive data	14*	(a) Give characteristics of study participants (eg demographic, clinical, social) and information on exposures and potential confounders	6	More extensive details of the survey methodology and sample characteristics are published elsewhere (21,22) and for demographic characteristics of the sample, see (22).
		(b) Indicate number of participants with missing data for each variable of interest	-	
		(c) *Cohort study*—Summarise follow-up time (eg, average and total amount)		
Outcome data	15*	*Cohort study*—Report numbers of outcome events or summary measures over time		
		Case-control study—Report numbers in each exposure category, or summary measures of exposure		
		Cross-sectional study—Report numbers of outcome events or summary measures	Tables 1-2	
Main results	16	(*a*) Give unadjusted estimates and, if applicable, confounder-adjusted estimates and their precision (eg, 95% confidence interval). Make clear which confounders were adjusted for and why they were included	Tables 1-2	
		(*b*) Report category boundaries when continuous variables were categorized	-	
		(*c*) If relevant, consider translating estimates of relative risk into absolute risk for a meaningful time period	-	

Continued on next page

For peer review only - http://bmjopen.bmj.com/site/about/guidelines.xhtml

Other analyses	17	Report other analyses done—eg analyses of subgroups and interactions, and sensitivity analyses	11	Table 2 presents the associations between lacking interest in sex and being distressed about this (as a measure/marker of severity), and the above sociodemographic, health, and sexual relationship/behaviour variables.
**Discussion**				
Key results	18	Summarise key results with reference to study objectives	11-12	We identified a broad range of factors, including some that have not been explored in previous large-scale surveys, that were associated with men's and women's reports of lacking interest in sex in a representative British population-based survey. Our findings, discussed below, revealed some gender similarities as well as some interesting gender differences. The strongest evidence for gender differences was for the relationship context variables, where associations with lacking interest in sex were much stronger for women than for men.
Limitations	19	Discuss limitations of the study, taking into account sources of potential bias or imprecision. Discuss both direction and magnitude of any potential bias	15	Limitations include the cross-sectional nature of the data, which mean that we are unable to infer temporality and causality. We only used a single item to assess lacking

Formatted Table

For peer review only

For peer review only - http://bmjopen.bmj.com/site/about/guidelines.xhtml

				interest in sex, although we additionally took account of whether those who reported this also reported that it caused them distress, as a way of trying to capture more problematic lack of interest. It is important to acknowledge, however, that these data do not necessarily correspond to clinical diagnoses. This sensitivity analysis enabled us to demonstrate that for most variables, similar associations exist regardless of whether or not distress was reported. Finally, we have tested many associations within this study and some will have been significant by chance.
Interpretation	20	Give a cautious overall interpretation of results considering objectives, limitations, multiplicity of analyses, results from similar studies, and other relevant evidence	16-17	The findings indicate that reporting lack of interest in sex is associated with a broad range of predictors across sociodemographic, relationship, sexual behaviour, and sexual attitudinal domains.
Generalisability	21	Discuss the generalisability (external validity) of the study results	~~15~~6	After weighting to adjust for unequal probabilities of selection and to match the British population in terms of age, gender and geographical region, the Natsal-3 sample was broadly representative, on key variables, of the British population as described by the 2011

For peer review only

For peer review only - http://bmjopen.bmj.com/site/about/guidelines.xhtml

			15	Census.<br>The sample is representative of those resident in private households in Britain i.e., not those living in institutions.Strengths of our study include the use of national probability sample survey data involving both men and women across a wide age range (21, 22).
**Other information**				
Funding	22	Give the source of funding and the role of the funders for the present study and, if applicable, for the original study on which the present article is based	18	Natsal-3 was supported by grants from the U.K. Medical Research Council (G0701757) and the Wellcome Trust (084840), with support from the Economic and Social Research Council and the Department of Health.

*Give information separately for cases and controls in case-control studies and, if applicable, for exposed and unexposed groups in cohort and cross-sectional studies.

Note: An Explanation and Elaboration article discusses each checklist item and gives methodological background and published examples of transparent reporting. The STROBE checklist is best used in conjunction with this article (freely available on the Web sites of PLoS Medicine at http://www.plosmedicine.org/, Annals of Internal Medicine at http://www.annals.org/, and Epidemiology at http://www.epidem.com/). Information on the STROBE Initiative is available at www.strobe-statement.org.

For peer review only

For peer review only - http://bmjopen.bmj.com/site/about/guidelines.xhtml
